# The ubiquitin-binding domain of DNA polymerase η directly binds to DNA clamp PCNA and regulates translesion DNA synthesis

**DOI:** 10.1016/j.jbc.2021.101506

**Published:** 2021-12-18

**Authors:** Kodavati Manohar, Prashant Khandagale, Shraddheya Kumar Patel, Jugal Kishor Sahu, Narottam Acharya

**Affiliations:** 1 Laboratory of Genomic Instability and Diseases, Department of Infectious Disease Biology, Institute of Life Sciences, Bhubaneswar, India; 2 Regional Centre for Biotechnology, Faridabad, India

**Keywords:** DNA replication, DNA polymerase, rad30, Polη, ubiquitin, PCNA, pip box motif, ubz, *Candida*, FBS, Fetal bovine serum, IDCL, Interdomain connecting loop, ITC, Isothermal calorimetry, PAD, Polymerase associated domain, pip, PCNA interacting motif, Pol, DNA polymerase, Rad30 (Polη), DNA polymerase eta, ubz, Ubiquitin zinc finger, UDB, Ubiquitin-binding domain, XPV, Xeroderma pigmentosum variant

## Abstract

DNA polymerase eta (Polη) is a unique translesion DNA synthesis (TLS) enzyme required for the error-free bypass of ultraviolet ray (UV)-induced cyclobutane pyrimidine dimers in DNA. Therefore, its deficiency confers cellular sensitivity to UV radiation and an increased rate of UV-induced mutagenesis. Polη possesses a ubiquitin-binding zinc finger (ubz) domain and a PCNA-interacting-protein (pip) motif in the carboxy-terminal region. The role of the Polη pip motif in PCNA interaction required for DNA polymerase recruitment to the stalled replication fork has been demonstrated in earlier studies; however, the function of the ubz domain remains divisive. As per the current notion, the ubz domain of Polη binds to the ubiquitin moiety of the ubiquitinated PCNA, but such interaction is found to be nonessential for Polη's function. In this study, through amino acid sequence alignments, we identify three classes of Polη among different species based on the presence or absence of pip motif or ubz domain and using comprehensive mutational analyses, we show that the ubz domain of Polη, which intrinsically lacks the pip motif directly binds to the interdomain connecting loop (IDCL) of PCNA and regulates Polη's TLS activity. We further propose two distinct modes of PCNA interaction mediated either by pip motif or ubz domain in various Polη homologs. When the pip motif or ubz domain of a given Polη binds to the IDCL of PCNA, such interaction becomes essential, whereas the binding of ubz domain to PCNA through ubiquitin is dispensable for Polη's function.

Y-family DNA polymerases (Pols) are a group of nonessential enzymes that play an imperative role during translesion DNA synthesis (TLS) (1, 2). They protect the stalled replication fork due to DNA lesion blockage from collapsing, prevent double-strand breaks and maintain an unperturbed cell cycle. The budding yeast possesses two Y family DNA pols—Polη and Rev1; whereas in humans, in addition to these, Polι and Polκ also replicate efficiently through distorting DNA lesions, albeit with low fidelity and low processivity (3). Timely recruitment of specific TLS pols to a specific lesion site and their regulated activity determine the stability of a cell’s genome. Deciphering underlying mechanisms by which TLS pols gain access to the template–primer junction and take over synthesis from the replicative pol is crucial to understand the dynamic behavior of the replication fork during translesion DNA synthesis (4).

In *Saccharomyces cerevisiae* and humans, genetic and biochemical studies have indicated that PCNA plays a pivotal role in the pol exchange process (5, 6). The TLS pols gain access to the replication fork by physically interacting with PCNA, which is mediated by 1 to 2 highly conserved PCNA interacting protein (pip) motif(s) present mostly in the noncatalytic region of the pols (7). Since TLS DNA polymerases are distributive in DNA synthesis, physical binding to PCNA increases their nucleotide incorporation efficiency on both undamaged and damaged DNA substrates without affecting their processivity (1). A pip motif consists of a consensus sequence of eight amino acids QxxhxxFF(or YF/FY/YY/FL), where x is any amino acid, and h is any hydrophobic residue. PCNA interaction motifs have been mapped in all the Y-family polymerases from *S. cerevisiae* and humans, except in Rev1 (4). While a single pip motif has been mapped in ScPolη and HsPolι, two pip motifs have been identified in human Pols-η and -κ (8, 9, 10, 11). Mutational inactivation of the ScPolη pip motif (_621_SKNILSFF_628_) abrogates its interaction with PCNA (11). Thereby, *S. cerevisiae* strains harboring F627A and F628A mutations in Polη exhibit enhanced UV sensitivity and UV-induced mutagenesis. In contrast to ScPolη, human Polη possesses two functional pips (_437_STDITSFL_444_ and _701_MQTLESFF_708_) at the C-terminal domain (8). Both the pips are functionally redundant and can substitute for one another. Only in the absence of both these motifs, HsPolη's interaction with PCNA on DNA, stimulation of its DNA synthetic activity, and colocalization with PCNA get abolished. In addition to the pip motif, several highly conserved ubiquitin (Ub)-binding domains (UBDs) have been identified in the C-terminal regions of Y-family pols, which regulate their TLS function (12). While Polη carries a single Zn^2+^-binding UBD, *i.e.*, ubz; Rev1, Pol-ι, and -κ possess two such motifs but without having Zn^2+^-binding ability (ubm). As PCNA gets monoubiquitinated *via* Rad6-Rad18 ubiquitination conjugating system during TLS (13, 14, 15, 16), it was proposed that the TLS polymerases bind to the ubiquitin moiety attached on PCNA through UBDs, and UBD-Ub-PCNA interaction is indispensable for the recruitment of TLS pols to PCNA. However, our subsequent mutational analysis in Sc- and Hs-Polη ruled out such a possibility, as mutations in the C_2_H_2_ motif or complete deletion of ubz domain had no perceivable effect on UV sensitivity or UV-induced mutagenesis (8, 17, 18). Thus, it was suggested that ubiquitin-binding on PCNA *via* its ubz domain is not a prerequisite for Polη to gain access to PCNA at the stalled replication site; rather, it may just function as a protein–protein interaction domain. Later studies reported that ubz binding to ub-PCNA increases Polη's retention time in the replication foci (19, 20, 21).

Since the pip motif of Polη is an essential structural component for TLS activity, all Polη homologs should possess such PCNA interaction motifs. To look for the conservation of the pip motif and its function in Polη across the kingdom, 77 Polη amino acid sequences from the different genera were aligned. Interestingly, the C-terminal amino acid sequence of Polηs showed maximum variations, and some of the members lacked either pip motif or ubz domain (Table 1 and Fig. S1). They were classified into three distinct categories based on the presence or absence of pip motif and ubz domain. Out of 77, 34 Polηs from species of fungi (11 genera), animals (22 genera), and a single plant genus belong to category-I that harbor both pip and ubz in their C-terminal region. The category-I Polηs from *Saccharomyces*, human, and *Xenopus* have been studied (22, 23). The Polηs from plant and protist sources possess pip motifs only in their C-terminal region and were grouped into category II (23 genera). It also implies that the role of the ubz domain of Polη is not evolutionarily conserved across the kingdom. *Arabidopsis thaliana* Polη has been studied, and its pip motif is essential for PCNA interaction and confers UV resistance in the *rad30Δ* mutant (24). To our surprise, we identified a group of Polηs only from fungi belonging to category III that seem to lack a pip motif at their usual location intrinsically. For example, Polηs from *Candida albicans*, *Neurospora*, *Schizosaccharomyces pombe*, and *Aspergillus* possess the highly conserved ubiquitin-binding C_2_H_2_ motif in their regulatory carboxyl tails, but not a pip motif. Since the pip motif is present in categories I and II Polηs and ubz is absent in category II, as demonstrated in *S. cerevisiae*, human, and *Arabidopsis* Polηs, the pip could be indispensable and ubz may be nonessential for the TLS function of Polηs in other organisms of these two categories as well. However, the recruitment of category III Polηs to the DNA lesion sites and their regulation by PCNA remain undetermined. Therefore in this study, we intended to decipher the underlying mechanism of the interaction of Polη from category III with PCNA. The *S. cerevisiae RAD30* homolog from the pathogenic yeast *C. albicans* has been characterized. The complementation analysis suggested that CaPolη suppresses UV sensitivity and UV-induced mutagenesis in a Polη-deficient *S. cerevisiae* strain (25, 26, 27, 28). By mutational analyses, we confirmed the absence of pip motif in CaPolη and provided the first conclusive evidence to suggest an indispensable role of the ubz domain of Polη in TLS function, which is also well supported by Polη from *S*. *pombe*. Further, considering previous reports and the current study, we suggest two modes of PCNA interaction by Polηs from various organisms.

**Table 1:** Classification of Polη from various organisms based on the presence or absence of pip motif and ubz domain

Sl. No.	Rad30 (Polη)	Organisms	No. of amino acids	Accession. No.	PIP	UBZ	Type of organisms
Category-I DNA polymerase eta (with both PIP motif (/s) and UBZ domain)							
1	ScRad30	*Saccharomyces cerevisisae*	632	Q04049.1	Yes	Yes	Fungi
2	KlRad30	*Kluyveromyces lactis*	645	CAH02669	Yes	Yes	Fungi
3	CgRad30	*Candida glabrata*	635	KTB22745.1	Yes	Yes	Fungi
4	CcRad30(1)	*Coprinopsis cinerea*	641	BAG68958	Yes	Yes	Fungi
5	CnRad30	*Cryptococcus neoformans*	715	XP_024512983	Yes	Yes	Fungi
6	ClRad30	*Crucibulum leave*	638	TFK40232	Yes	Yes	Fungi
7	CmRad30	*Coprinopsis marcescibilis*	648	TFK27153	Yes	Yes	Fungi
8	SsRad30(1)	*Suillus subaureus*	602	KAG1821793	Yes	Yes	Fungi
9	TtRad30	*Thelephora terrestris*	690	KAF9790376	Yes	Yes	Fungi
10	PpRad30(1)	*Panaeolus papilionaceus*	640	KAF9036173	Yes	Yes	Fungi
11	HsRad30(1)	*Homo sapiens*	713	Q9Y253.1	Yes	Yes	Animal
12	GgRad30(1)	*Gorilla gorilla*	713	XP_030867852.1	Yes	Yes	Animal
13	CdRad30	*Camelus dromedaries*	727	KAB1262039	Yes	Yes	Animal
14	AmRad30	*Alligator mississippiensis*	707	KYO23933	Yes	Yes	Animal
15	HmRad30	*Hymenolepis microstoma*	606	CDS33619	Yes	Yes	Animal
16	JjRad30	*Jaculus jaculus*	670	XP_004649790	Yes	Yes	Animal
17	EeRad30	*Elephantulus edwardii*	680	XP_006882015	Yes	Yes	Animal
18	LhRad30	*Labeo rohita*	658	RXN27300	Yes	Yes	Animal
19	EgRad30(1)	*Eechinococcus granulosus*	578	EUB55503	Yes	Yes	Animal
20	SvRad30	*Sturnus vulgaris*	699	XP_014731573.1	Yes	Yes	Animal
21	PbRad30	*Python bivittatus*	675	XP_007438340.1	Yes	Yes	Animal
22	PmRad30(1)	*Parus major*	701	XP_015476804.1	Yes	Yes	Animal
23	DmRad30	*Drossophila melanogaster*	885	NP_649371.2	Yes	Yes	Animal
24	RnRad30	*Rattus norvegicus*	689	NP_001101674.1	Yes	Yes	Animal
25	BtRad30	*Bos taurus*	711	NP_001029622.1	Yes	Yes	Animal
26	DrRad30	*Danio rerio*	749	NP_001035337.2	Yes	Yes	Animal
27	MmRad30(1)	*Mus musculus*	694	NP_109640.1	Yes	Yes	Animal
28	PtRad30	*Panthera tigris*	712	XP_015394402	Yes	Yes	Animal
29	GgRad30(2)	*Gallus gallus*	693	NP_001001304.2	Yes	Yes	Animal
30	XlRad30	*Xenopus leavis*	684	NP_001087074.1	Yes	Yes	Animal
31	SsRad30(2)	*Salmo salar*	782	XP_014010099.1	Yes	Yes	Animal
32	AoRad30	*Amphiprion ocellaris*	743	XP_023136504.1	Yes	Yes	Animal
33	PnRad30	*Phytophthora nicotianae*	583	KUF78308.1	Yes	Yes	Plant
Category-II DNA polymerase eta (with only PIP motif)							
1	TbRad30	*Trypanosoma brucei*	525	XP_011777388	Yes	No	Protist
2	BsRad30	*Bodo saltans*	541	CUG54578	Yes	No	Protist
3	LmRad30	*Leishmania major*	760	XP_001682996.1	Yes	No	Protist
4	FsRad30	*Fistulifera solaris*	601	GAX09505.1	Yes	No	Protist
5	CeRad30	*Caenorhabditis elegans*	584	BAE72703	Yes	No	Protist
6	GsRad30	*Galdieria sulphuraria*	546	EME29580.1	Yes	No	Protist
7	PpRad30(2)	*Porphyridium purpureum*	666	KAA8495432	Yes	No	Protist
8	AtRad30	*Arabidopsis thaliana*	672	Q8H2D5.1	Yes	No	Plant
9	BdRad30	*Brachypodium distachyon*	631	XP_010232348	Yes	No	Plant
10	HsRad30(2)	*Hibiscus syriacus*	705	XP_039070204	Yes	No	Plant
11	SlRad30	*Solanum lycopersicum*	726	XP_010318257	Yes	No	Plant
12	CaRad30(1)	*Capsicum annuum*	696	PHT94068.1	Yes	No	Plant
13	CsRad30(1)	*Cannabis sativa*	705	XP_030480064	Yes	No	Plant
14	OsRad30	*Oryza sativa*	653	XP_015649373.	Yes	No	Plant
15	ZjRad30	*Ziziphus jujube*	703	XP_015874760	Yes	No	Plant
16	CsRad30(2)	*Citrus sinensis*	756	XP_024951002	Yes	No	Plant
17	AhRad30	*Arachis hypogaea*	752	QHO60128	Yes	No	Plant
18	BnRad30	*Brassica napus*	674	XP_013681659	Yes	No	Plant
19	RsRad30	*Raphanus sativus*	675	XP_018477985	Yes	No	Plant
20	CcRad30(2)	*Cajanus cajan*	706	XP_029130107	Yes	No	Plant
21	PmRad30(2)	*Prunus mume*	724	XP_008225911	Yes	No	Plant
22	EgRad30	*Eucalyptus grandis*	724	XP_010051816	Yes	No	Plant
23	AoRad30	*Asparagus officinalis*	633	XP_020256660	Yes	No	Plant
Category-III DNA polymerase eta (with only UBZ domain)							
1	CaRad30(2)	*Candida alibicans*	640	KHC87745.1	No	Yes	Fungi
2	CaRad30(3)	*Cryptosporidium andersoni*	711	OII73304	No	Yes	Fungi
3	NcRad30	*Neurospora crassa*	672	EAA34884.1	No	Yes	Fungi
4	DhRad30(1)	*Debaryomyces hansenii*	770	CAG89102.2	No	Yes	Fungi
5	UmRad30	*Ustilago maydis*	865	XP_011386064.1	No	Yes	Fungi
6	YpRad30	*Yarrowia lipolytica*	640	KAE8171010.1	No	Yes	Fungi
7	BmRad30	*Blastomyces dermatitidis*	658	EGE81585	No	Yes	Fungi
8	StRad30	*Scheffersomyces stipites*	733	XP_001382310.2	No	Yes	Fungi
9	AnRad30	*Aspergillus nidulens*	674	CBF77016.1	No	Yes	Fungi
10	GcRad30	*Geotrichum candidum*	741	CDO56699.1	No	Yes	Fungi
11	OoRad30	*Orbilia oligospora*	780	TGJ67087	No	Yes	Fungi
12	FoRad30	*Fusarium oxysporum*	683	KNA99902.1	No	Yes	Fungi
13	PgRad30	*Penicillium griseofulvum*	647	KXG49715.1	No	Yes	Fungi
14	CsRad30(3)	*Colletotrichum salici*	581	KXH50573.1	No	Yes	Fungi
15	NgRad30	*Nannizzia gypsea*	646	EFR00972.1	No	Yes	Fungi
16	McRad30	*Microsporum canis*	646	XP_002849170.1	No	Yes	Fungi
17	MmRad30(2)	*Microthyrium microscopicum*	640	KAF2673775	No	Yes	Fungi
18	MrRad30	*Malassezia restricta*	641	AXA51208	No	Yes	Fungi
19	DhRad30(2)	*Diaporthe helianthi*	659	POS72209	No	Yes	Fungi
20	BgRad30	*Blumeria graminis*	664	EPQ66565	No	Yes	Fungi
21	AfRad30	*Alectoria fallacina*	694	CAF9922873.1	No	Yes	Fungi
22	SpRad30	*Schizosaccharomyces pombe*	872	CAA16862.1	No	Yes	Fungi

## Results

### C-terminal domain of CaPolη is indispensable for PCNA interaction and UV-lesion bypass

**Figure 1:**
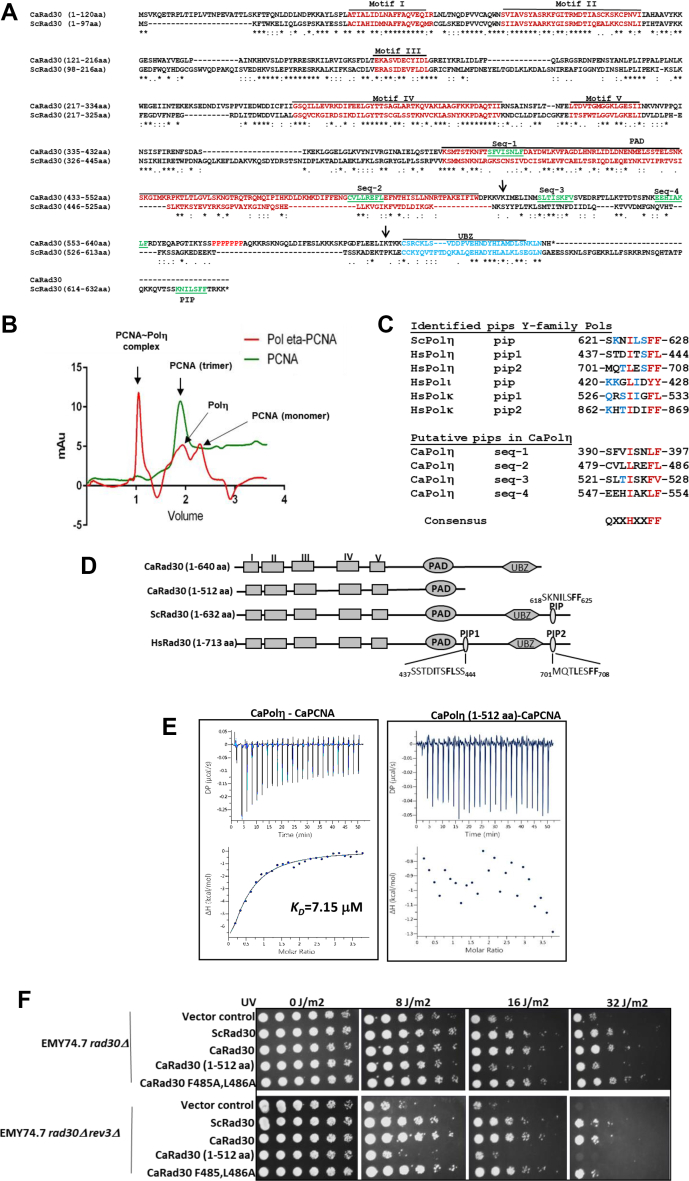
**Identification of PCNA interaction region in CaPolη.***A*, amino acid alignment between Ca- and Sc-Polη was carried out by EMBOSS Needle; identical amino acids are marked by ∗ and similar amino acids were marked by: *symbols*. All the important motifs and domains are underlined, including Motif I to V, PAD, ubz and pip. Putative pip sequences were labeled as Seq-1 through Seq-4 in the C-terminal domain. *B*, size-exclusion chromatograms of CaPCNA (*green*) and a mixture of CaPolƞ-CaPCNA (*red*); CaPCNA elutes with the peak fractionation at ∼1.7 ml and a complex of CaPol ƞ-PCNA displayed a peak at ∼1.2 ml. *C*, putative pip sequences of CaPolη were compared with the already identified pip motifs of other Y-family pols. *D*, line diagram showing various domains of Polηs and a C-terminal deletant CaPolη (1–512). *E*, determination of binding kinetics of wild type or truncated CaPolƞ (1–512) with CaPCNA by ITC. In each panel, the *upper half* shows the measured heat exchanges during each PCNA protein injection. The *lower half* of each panel shows the enthalpic changes as a function of the molar ratio of the proteins where PCNA was considered as a trimer. *F*, UV sensitivities of *S. cerevisiae* strains. Cells of genomic *rad30Δ* and *rad30Δrev3Δ* deletion *S. cerevisiae* strains harboring YEP-*ADH1*p or YEP-*ADH1*p-ScPolη or YEP-*ADH1*p-CaPolη or YEP-*ADH1*p-CaPolη (1–512) or YEP-*ADH1*p-CaPolη F485A,L486A plasmids from an overnight SD-Ura culture were serially diluted and spotted onto SD-Ura plates. The culture plates were irradiated with the indicated doses of UV radiation, covered with aluminum foil, incubated at 30 °C for 3 days, and then photographed. ITC, isothermal calorimetry.

The primary sequence alignment of CaPolη with ScPolη suggested that in contrast to ScPolη, CaPolη is naturally truncated immediately after the C_2_H_2_ ubz domain and lacks a canonical pip sequence at its extreme C-terminal tail (Fig. 1*A*). Since similar to CaPolη, other 22 Polηs of category III also lack the pip motif (Table 1 and Fig. S1), one would presume that these Polηs either do not bind directly to PCNA or may have a different mode of PCNA interaction. To establish a direct physical interaction of CaPolη with CaPCNA, molecular size-exclusion chromatography was performed (Fig. 1*B*). The purified wild-type CaPolη protein (1–640 aa, ∼70 kDa) was mixed with CaPCNA in a 1:1 M ratio and allowed to resolve in analytical size-exclusion chromatography. For comparison, CaPCNA alone was also passed through the column, and its elution profile was recorded. As reported earlier also, CaPCNA (∼90 kDa) eluted as a trimer with an elution volume of ∼1.7 ml (29). However, when the mixture of CaPolη-CaPCNA proteins was resolved, an early major elution peak at about ∼1.2 ml corresponding to a complex of Polη-PCNA and two smaller peaks at ∼1.7 ml and 2.4 ml elution volumes corresponding to free proteins were observed. The shifting of CaPCNA from 1.7 ml to 1.2 ml elution volume suggested that CaPCNA directly interacts and coelutes with CaPolη by forming a stable complex in solution and may regulate Polη's activity; hence warrants a detailed investigation.

The direct interaction of CaPolη with CaPCNA implicated the involvement of yet unidentified pip motif or domain of CaPolη in PCNA binding. Four putative noncanonical pip sequences: Seq-1 (390SFVISNLF397), Seq-2 (479CVLIREFL486), Seq-3 (521SLTISKFV528), and Seq-4(547EEHIAKLF554) in and around the PAD of CaPolη may bind to PCNA (Fig. 1*C*). In addition, a unique proline-rich sequence located in between Seq-4 and ubz domain of CaPolη is observed that might provide additional structural flexibility to the C-terminal portion to be engaged in protein–protein interaction. To identify a region of CaPolη involved in PCNA interaction, we generated a deletion construct by inserting a stop codon at amino acid position 513 (K513) of CaPolη. The truncated CaPolη spanning residues 1 to 512 encodes for the whole catalytic domain up to the PAD (Fig. 1*D*), and similarly truncated Polηs both from *S. cerevisiae* and human are known to retain DNA polymerase activity as good as the full-length enzymes (8, 30). The wild-type and truncated CaPolη were purified, and their interaction with CaPCNA was monitored by isothermal calorimetry (ITC) analysis. ITC allows estimating binding affinities between two proteins in the native environment. CaPCNA was injected into the sample cell of the calorimeter either containing wild-type or the catalytic domain of CaPolη 1 to 512 aa, and the binding between the two proteins was analyzed (Fig. 1*E* and Table 2). While the titration of CaPCNA against the full-length Polη resulted in an exothermic reaction, and the estimated values of ΔH, ΔG, and *K*_*D*_ were −22.1 kcal/mol, −7.02 kcal/mol, and 7.15 μM, respectively, no perceivable change in the heat was detected when PCNA was titrated against the truncated CaPolη 1 to 512 aa or the buffer alone (Fig. S2). It suggested that the amino-terminal catalytic domain of CaPolη is not involved in PCNA interaction. Since CaPolη functionally complements ScPolη and protects the fungal cells from UV-induced DNA damages (17, 25, 26), to further strengthen our finding, various Polη orfs were expressed under the constitutive *ADH1* promoter in *rad30Δ* and *rad30Δrev3Δ S. cerevisiae* strains, and susceptibility to UV irradiation was determined. Polη and Polζ (Rev3-Rev7) function in the error-free and error-prone branches of TLS of UV lesions, respectively; therefore, double deletant *S. cerevisiae* (*rad30Δrev3Δ*) strain becomes hypersensitive to UV radiation than the individual null strain (31). As depicted in Figure 1*F*, the truncated CaPolη 1 to 512 aa failed to complement the *rad30* null strains of *S. cerevisiae*. The transformants carrying truncated CaPolη 1 to 512 aa showed severe growth impairment when they were irradiated to UV at different doses (16–32 J/m^2^), and the sensitivity was similar to that of the vector control. Thus, the catalytic domain consisting of the first 512 amino acids of CaPolη is insufficient to carry out lesion bypass in the cell as well as inefficient in PCNA binding. Moreover, a similar complementation assay with CaPolη F485A and L486A mutations that supported the growth of cells upon UV exposure ruled out the involvement of Seq-2 of PAD in PCNA interaction (Fig. 1*F*). These results implicated the requirement of the C-terminal domain region spanning residues 513 to 640 aa of CaPolη in TLS and possibly in PCNA interaction.

**Table: 2:** Kinetic parameters determination of PCNA binding with Polη or its peptide sequence

Parameters	CaPolη *versus* CaPCNA	Ubz peptide *versus* CaPCNA	Ubz peptide *versus* CaPCNA-90	ScPolη *versus* ScPCNA	Ubz peptide *versus* ScPCNA
Cell concentration	10 μM	20 μM	20 μM	10 μM	20 μM
Syringe concentration	200 μM	200 μM	200 μM	200 μM	200 μM
Stoichiometry	0.315	0.355	0.316	0.31	0.34
*K*_*D*_	7.15 μM	19.8 μM	39 μM	15.1 μM	15. 6μM
*ΔH*	−22.1	−80	−79.9	−79.9	−24.2
*ΔG*	−7.02	−6.42	−6.02	−6.58	−8.56
*−TΔS*	15.1	73.5	73.9	73.3	17.6

### Ubz domain of CaPolη is essential for CaPolη's function in *S. cerevisiae* and interaction with PCNA

**Figure 2:**
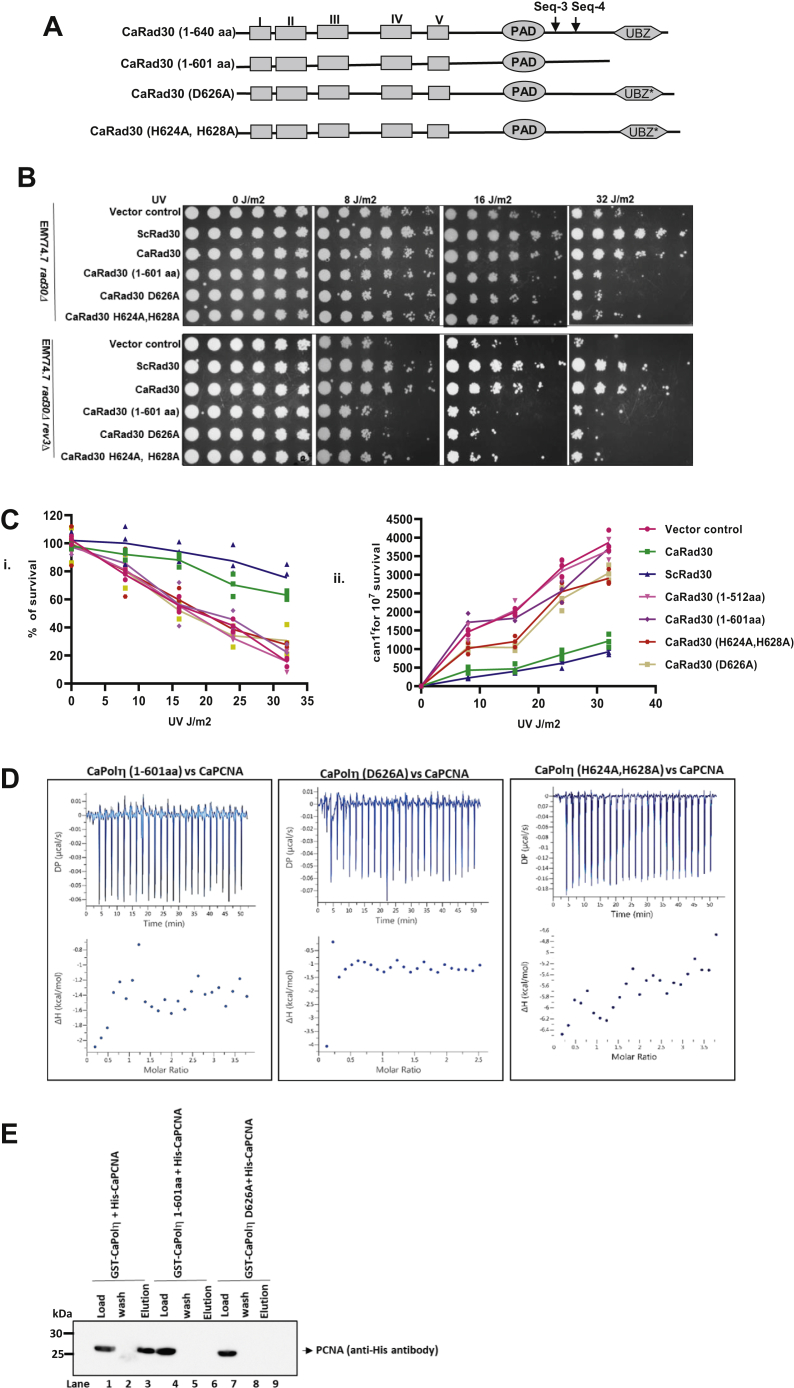
**Ubz domain of CaPolη is essential TLS activity.***A*, a line diagram showing the deletion and site-directed mutations in the ubz domain of CaPolη. *B*, *rad30Δ S. cerevisiae* cells harboring YEP-*ADH1*p or YEP-*ADH1*p-ScPolη or YEP-*ADH1*p-CaPolη or YEP-*ADH1*p-CaPolη (1–601) or YEP-*ADH1*p-CaPolη D626A or YEP-*ADH1*p-CaPolη (H624A,H628A) mutation plasmids from an overnight SD-Ura culture were serially diluted and spotted onto SD-Ura plates. The culture plates were irradiated with indicated doses of UV radiation, covered with aluminum foil, incubated at 30 °C for 3 days, and then photographed. *C*, appropriate dilutions of *rad30Δ* deletion *S. cerevisiae* strains harboring the above-mentioned mutations were plated onto SD medium for viability determinations (i) and onto SD lacking arginine but containing canavanine for mutagenesis assay (ii). UV irradiation was done as indicated. Following UV irradiation, plates were incubated in the dark, and colonies were counted after 3 to 5 days. Experiments were performed in triplicate, and the average was plotted. *D*, determination of binding kinetics of truncated CaPolƞ (1–601) (i) or D626A (ii) or H624A, H628A (iii) Polη mutants with PCNA by ITC. In each panel, the *upper half* shows the measured heat exchanges during each PCNA protein injection. The *lower half* of each panel shows the enthalpy changes as a function of the molar ratio of the two proteins where PCNA is considered as a trimer and Polη as a monomer. *E*, GST pull-down of His-PCNA by CaPolη. Beads of GST-CaPolη (lanes 1–3) or GST-CaPolη 1 to 601 (lanes 4–6) or GST-CaPolη D626A (lanes 7–9) were mixed with PCNA in equilibration buffer and after the incubation beads were washed and the bound PCNA was eluted by protein loading dye. The fractions were resolved in 12% SDS PAGE, blotted to the membrane, and developed by the anti-His antibody. Lanes 1, 4, and 7 are 10% of the load; Lanes 2, 5, and 8 are 10% of the third washings; Lanes 3, 6, and 9 are the total eluates. TLS, translesion DNA synthesis.

The above results indicated that the C-terminal portion of CaPolη containing two putative pip sequences: Seq-3 and Seq-4, and one ubz domain could be involved in PCNA interaction. To map the precise location of PCNA binding, another deletant CaPolη 1 to 601 aa was constructed that lacks the last 39 residues encompassing the ubz domain but retains Seq-3 and Seq-4 (Fig. 2*A*). Similar to CaPolη 1 to 512 aa, Polη 1 to 601 aa also conferred significant growth retardation at 16 to 32 J/m^2^ of UV irradiation in both *rad30Δ* and *rad30Δrev3Δ S. cerevisiae* strains implying that both Seq-3 and Seq-4 are not essential for CaPolη's TLS activity in the cell and are unlikely to be involved in PCNA interaction (Fig. 2*B*). Moreover, it suggested the critical role of the C-terminal 39 amino acids comprising the ubz domain of CaPolη in TLS activity. To ascertain the function of ubz domain, two D626A and H624A, H628A of CaPolη mutants were generated and their ability to suppress UV sensitivity of Polη-deficient *S. cerevisiae* strains was examined. Both the CaPolη mutants did not confer any cellular protection to UV radiation, and growth was inhibited drastically (Fig. 2*B*). The complementation analysis was reconfirmed by estimating the cell survivability upon UV exposure; where about 85 to 95% of cells expressing wild-type Polη conferred resistance, only ∼10 to 35% *rad30Δ* cells harboring CaPolη mutants survived after 32 J/m^2^ of UV treatment (Fig. 2*C*, i). Similarly, UV-induced mutation rate was estimated by counting the number of canavanine-resistant colonies of various *S. cerevisiae* strains harboring CaPolη constructs. The *rad30Δ* strain or strains harboring truncated or ubz mutants of CaPolη were highly mutagenic as more number colonies (∼4000 per 10^7^ cells) grew as canavanine-resistant, as opposed to the strains expressing wild-type Polη (∼750 per 10^7^ cells; Fig. 2*C*, ii). This result suggested that the ubz domain of CaPolη plays an essential role in the error-free bypass of UV-induced lesions and possibly in PCNA interaction. Subsequently, ITC and GST-pull-down assays were carried out to determine the role of ubz in PCNA binding. About 10 μM of each mutant CaPolη 1 to 601 aa, D626A, and H624A, H628A proteins were placed in the calorimetric cell, and the change in the heat was monitored after each CaPCNA injection (Fig. 2*D*). Our ITC assays revealed no significant net heat change when PCNA was titrated against the mutants of Polη suggesting no detectable interaction between the proteins. Similarly, the GST-pull-down assay authenticated the ITC results where wild-type GST-Polη pulled down PCNA from the mixture, whereas the mutant Polη proteins failed to pull down PCNA (Fig. 2*E* compare lane 3 with 6 and 9). From these observations, we concluded that the ubz domain of CaPolη directly binds to PCNA, and this interaction is essential for its *in vivo* activity.

### Ubz domain of Polη is sufficient for PCNA interaction

**Figure 3:**
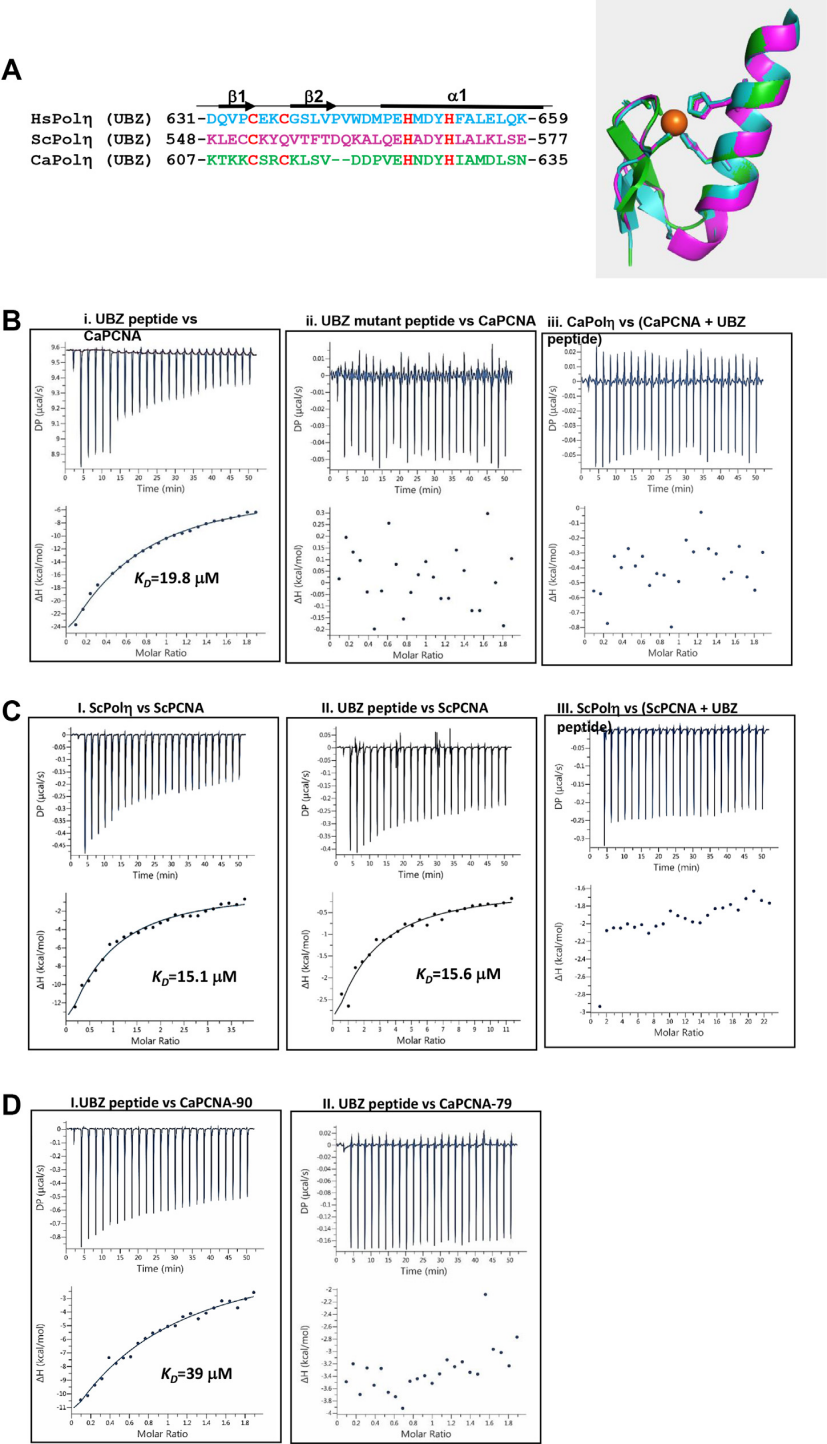
**Direct interaction of ubz of CaPolη with PCNA.***A*, sequence alignment and a superimposable model structures of ubz of Human (Hs, *cyan*), *S. cerevisiae* (Sc, *purple*) and *C. albicans* (Ca, *green*) Polƞs. Secondary structures are highlighted. Human Polη ubz structure 3WUP was used as a template for modeling. Zn^2+^ (*orange*) coordinated to the C_2_H_2_ motif is also shown. *B*, determination of the binding kinetics of ubz peptide (i) or ubz mutant peptide (ii) of CaPolη mutants with PCNA by ITC. An interaction of a mixture of CaPCNA and ubz peptide with CaPolη injection was also determined (iii). In each panel, the *upper half* shows the measured heat exchanges during each PCNA (i and ii) or CaPolη (iii) protein injection. The *lower half* of each panel shows the enthalpy changes as a function of the molar ratio of the two proteins where PCNA was considered as a trimer. *C*, determination of binding kinetics of ScPolη with ScPCNA (i) or ubz peptide with ScPCNA (ii) mixture of ScPCNA and ubz peptide with ScPolη (iii) by ITC. In each panel, the *upper half* shows the measured heat exchanges during each ligand injection. The *lower half* of each panel shows the enthalpy changes as a function of the molar ratio of the two proteins where PCNA was considered as a trimer. *D*, determination of the binding kinetics of ubz peptide with ScPCNA-90 (i) or ubz peptide with ScPCNA-79 (ii) by ITC. In each panel, the *upper half* shows the measured heat exchanges during each PCNA injection. The *lower half* of each panel shows the enthalpy changes as a function of the molar ratio of the two proteins where PCNA was considered as a trimer. ITC, isothermal calorimetry.

Unlike ScPolη and human Polη, where the ubz domain is dispensable, in CaPolη, ubz is essential for TLS activity. To understand whether any sequence or structural differences of the ubz domains in these Polηs have an impact on functional differences, we used the solution structure of the ubz domain of human Polη as a template and determined the structure of the ubz domain of both Sc- and Ca-Polηs by computational modeling (Fig. 3*A*). While human and *C. albicans* Polηs possess a canonical C_2_H_2_-Zn^2+^-binding domain, ScPolη ubz is noncanonical as the second cysteine residue is replaced by glutamine. Although there is hardly any similarity in the primary sequences among these ubz domains except for the positions of cysteines and histidines that coordinate the metal cofactor, they adopt a classical superimposable C_2_H_2_ zinc finger structure characterized by a β-fold comprised of two short antiparallel strands and a carboxyl-terminal α-helix. The two cysteines are located on the fingertip made by the two β-strands, and the two histidines are located on the α-helix. The α-helix shows the maximum sequence identity and is found to be involved in interaction with ubiquitin (32). Our structure predictions did not reveal any differences in the ubz structures; however, the *in vivo* TLS role of ubz at least in CaPolη is found to be distinct from the other two Polηs suggesting the role of ubz to be evolved differently in different species. Since the ubz domain of CaPolη is necessary for its TLS function and PCNA interaction, we wanted to determine whether the ubz domain is sufficient for PCNA binding. A 29-mer ubz peptide (607-KTKKCSRCKLSVDDPVEHNDYHIAMDLSN-635) and its corresponding mutant peptide (607-KTKKCSRCKLSVDDPVEANDYAIAMDLSN-635) were synthesized, and their interaction with CaPCNA was validated by ITC analysis. While PCNA titration against the wild-type ubz peptide taken in the calorimetric cell resulted in the release of heat, it did not show any sign of binding with the mutant peptide as no net energy change was detected (Fig. 3*B*, i and ii). The kinetic parameters upon the binding of wild-type ubz peptide with PCNA were determined as ΔH = −80 kcal/mol, ΔG = −6.42 kcal/mol, and *K*_*D*_ = 19.8 μM (Table 2). As the binding affinity of ubz peptide toward PCNA is about two-fold less than that of the full-length Polη, the contribution of neighboring residues of ubz domain for the stable interaction with PCNA appears to be substantial. Next we examined whether the ubz peptide can inhibit the binding of PCNA to Polη. An equimolar mixture of the ubz peptide and PCNA was injected into the calorimetric cell having CaPolη to examine the interference of peptide on the interaction between PCNA and Polη by ITC. As shown in Figure 3*B*, iii, PCNA∼ubz peptide mix did not bind to CaPolη as the profile showed no heat exchange. This result suggested that ubz peptide and CaPolη completely inhibit each other's binding to PCNA and most likely both the ligands bind to the same surface of PCNA. In a similar assay, we also found that ScPolη and ubz peptide derived from CaPolη make stable complexes individually with ScPCNA with about equal affinities (*K*_*D*_ = 15.6 μM), but a mixture of ScPCNA and ubz peptide excluded from binding to ScPolη (Fig. 3*C* and Table 1). These results suggested that irrespective of the sources of PCNA, ubz peptide interacts with them with equal affinities, and ubz peptide blocks the binding of Polη to PCNA, most probably by forming a ubz-PCNA complex that prevented PCNA from binding to Polη. It also indicates that the interaction of ubz peptide and Polη with PCNA is mutually exclusive.

### Ubz domain mimics pip motif in binding to the interdomain connecting loop (IDCL) of PCNA

The PCNA interaction motif of the ligand occupies the hydrophobic pocket formed by the IDCL that connects the two globular domains of a PCNA protomer (33, 34). Most of the structural characterization studies suggest that the pip motif forms a 3_10_ helix that snuggly fits into the hydrophobic pocket of PCNA. To map the binding site of ubz peptide in PCNA, we used two available CaPCNA mutant proteins CaPCNA-79 and CaPCNA-90. In PCNA-79, two key hydrophobic residues L126 and I128 of the interdomain connecting loop were mutated to alanine, whereas in PCNA-90, the extreme C-terminal tail possesses P253A and K254A mutations. Most of the interacting proteins bind to any of these two regions of a trimeric PCNA ring (4). Our ITC analyses revealed that ubz peptide binds to PCNA-90 but not to PCNA-79. Kinetic parameters suggested the interaction between ubz and PCNA-90 to be an exothermic reaction, and the equilibrium dissociation constant was determined to be in the range of 39 μM which is about two-fold higher than that of the ubz-CaPCNA complex (Fig. 3*D*). To further strengthen our finding, a GST pull-down assay was carried out to pull down PCNA from an equimolar mixture of GST-CaPolη with wild-type or mutants of CaPCNA proteins. While GST-CaPolη was able to pull down most of the wild-type PCNA and PCNA-90 proteins from the solution (compare lane 1 with 3, and 7 with 9), it failed to bind PCNA-79 (compare lane 4 with 6) (Fig. S3*A*). Both the assays suggested that the hydrophobic cavity formed by the IDCL of PCNA is the interaction site of CaPolη, mediated by its ubz domain.

### Species-specific role of pip motif and ubz domain of Polη in TLS

**Figure 4:**
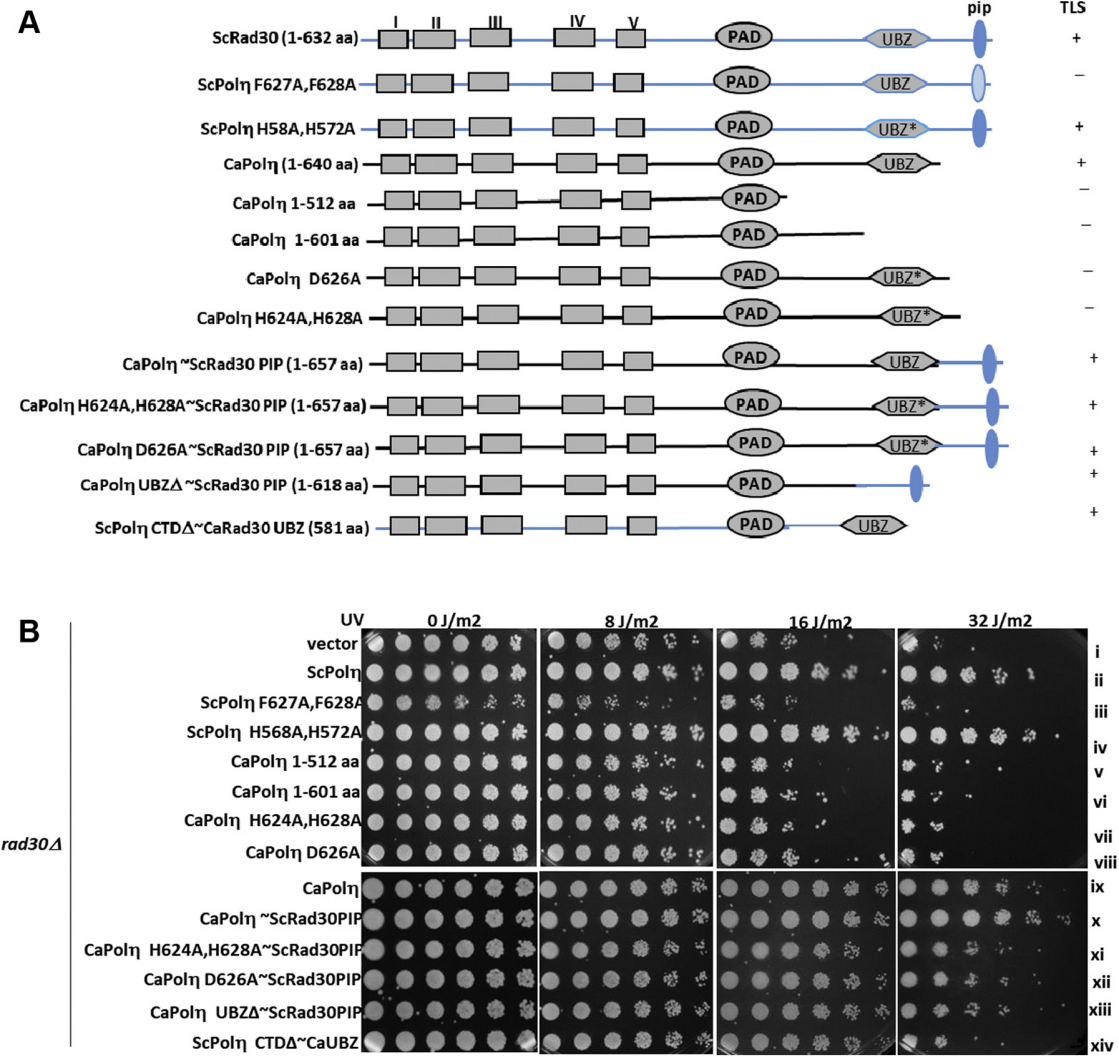
**Differential UV sensitivity conferred by various chimeric Polηs.***A*, a line diagram showing the various fusion constructs of CaPolη. As CaPolη intrinsically lacks a pip motif, pip sequence from ScPolη was fused to wild-type and ubz mutants of CaPolη. Similarly, the C-terminal domain of ScPolη was replaced with the C-terminal domain of CaPolη in ScPolηΔC∼CaRad30ubz. TLS efficiency of these transformants is highlighted. *B*, cells of genomic *rad30Δ S. cerevisiae* strains harboring vector alone (YEP-*ADH1*p) or ScPolη or ScPolη pip (F627A,F628A) mutant or ScPolη H568A,H572A or CaPolη 1 to 512 aa or CaPolη 1 to 601 aa or CaPolη D626A or CaPolη H624A,H628A or CaPolη or CaPolη∼ScRad30pip or CaPolη H624A,H628A∼ScRad3 pip or CaPolη D627A∼ScRad30pip or CaPolη UBZΔ∼ScRad30pip or ScPolη CTDΔ∼CaRad30ubz plasmids from an overnight SD-Ura culture were serially diluted and spotted onto SD-Ura plates. The culture plates were irradiated with the indicated doses of UV radiation, covered with aluminum foil, incubated at 30 °C for 3 days, and then photographed.

Since ubz of CaPolη functionally mimics the pip motif of ScPolη, to understand the species-specific roles of these two critical motifs during TLS, an array of hybrid Polη constructs were generated and analyzed (Fig. 4). The pip box sequence of ScPolη (KQVTSSKNILSFFTRKKstop) was fused just before the termination codon in wild-type and mutant CaPolη orfs to generate CaPolη∼ScRad30pip, CaPolη D626A∼ScRad30pip, and CaPolη H624A, H628A∼ScRad30pip. The chimeric CaPolη UBZΔ∼ScRad30pip (1–618 aa) was also generated by fusing the ScPolη pip sequence immediately after the catalytic domain; therefore, it does not retain its ubz domain but possesses the pip motif from ScPolη. Similarly, a C-terminal fragment containing ubz amino acid sequences from CaPolη (PKLECSRCKLSVDDPVEHNDYHIAMDLSNKLNNHstop) was fused to the catalytic domain of ScPolη to generate a chimeric ScPolη CTDΔ∼CaRad30ubz that mimics with the C-terminal domain of CaPolη, lacking the pip sequence. As shown in Figure 4, *A* and *B*; F627A, F628A mutations in the pip box of ScPolη abrogated its TLS function and displayed equal UV sensitivity with *rad30Δ* strain but H568A, H572A mutations in ubz of ScPolη did not affect survival and restored wild-type level of UV sensitivity to the *rad30Δ* strain (compare sectors i and iii with ii and iv). Thus, while the pip motif of ScPolη plays a critical role in TLS, the ubz of ScPolη is nonessential, and this result further authenticates our earlier reports (15, 17). However, as shown here and in Figure 3*B*, the ubz mutants of CaPolη are defective in conferring UV resistance (sectors v–vii). Interestingly, the carboxyl-terminal fusion of ScRad30-pip sequence to these mutants rescued the UV sensitive phenotype in the *rad30Δ* strain. At 16 to 32 J/m^2^ of UV, a significant level of growth was observed for *rad30Δ* strain expressing CaPolη D625A∼ScRad30pip or CaPolη H624A, H628A∼ScRad30pip or CaPolη ubzΔ∼ScRad30pip chimeras than the vector control alone suggesting that presence of ScRad30 pip motif alone is sufficient to suppress UV susceptibility, and ubz domain is dispensable (compare sectors xi–xiii with i). Interestingly, a similar fusion of ScRad30-pip to wild-type CaPolη conferred slightly better resistance to UV irradiation than the *rad30Δ* strain expressing CaRad30 (compare sectors x with ix). Although the presence of ubz of CaPolη is essential for its TLS function, the fusion of this domain alone to the catalytic domain of ScPolη (ScPolη CTDΔ∼CaRad30 ubz) partially rescued the UV-sensitive phenotype of *rad30* deletion strain (sector xiv, up to 16 J/m^2^). At a higher dose of UV radiation (32 J/m^2^), ubz fused ScPolη failed to carry out efficient TLS. We obtained almost similar results when we tested these hybrid Polηs in *rad30Δrev3Δ*, yet another Polη-deficient *S. cerevisiae* strain (Fig. S3*B*). Overall, these results suggest that a ubz domain can substitute for a pip motif in CaPolη for TLS activity but not the other way in ScPolη. Probably, not so conserved flanking sequences in Polηs might be playing a role in deciding whether ubz can function as a pip motif or not.

### Functional analyses of ubz domain of CaPolη in *C. albicans*

**Figure 5:**
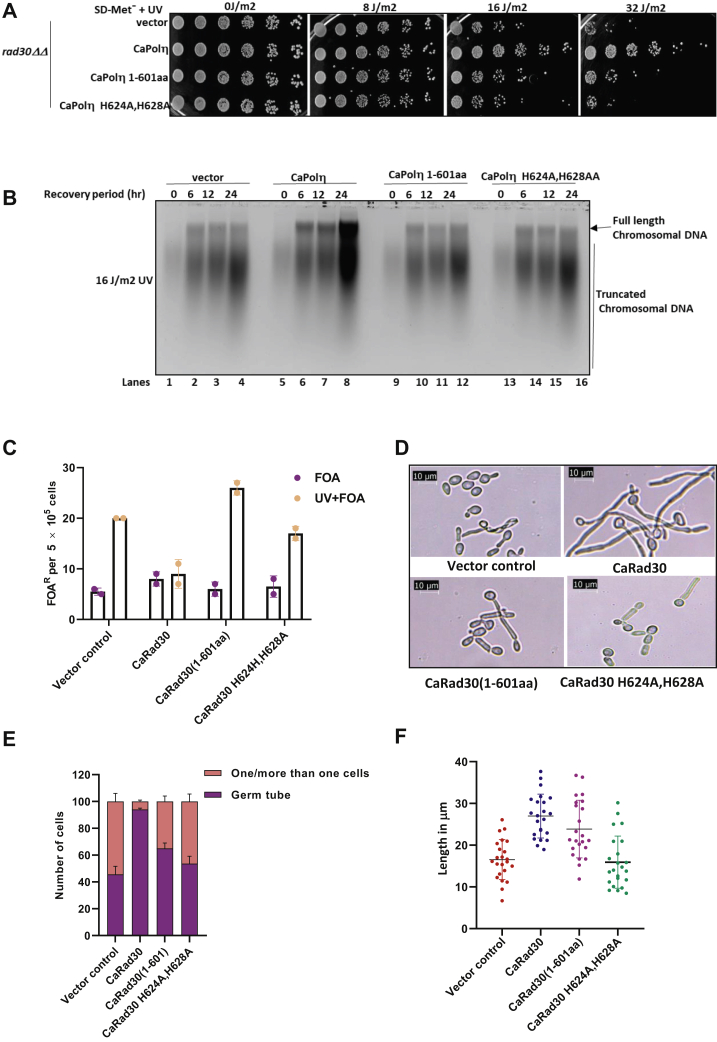
**Functional analyses of ubz domain of CaPolη in*****C. albicans.**A*, cells of *rad30*ΔΔ *C. albicans* strain expressing wild-type or mutants of CaPolη (1–601 aa and H624A, H628A) were grown overnight, serially diluted and spotted on SD without Methionine and Uracil plates, and irradiated with indicated UV dosages, incubated at 30 °C for 3 days, and imaged. *B*, various strains of *C. albicans* were exposed to 16 J/cm^2^ UV, followed by recovery, and the cells were harvested at different time points. Genomic DNA was isolated and allowed to resolve in an alkaline agarose gel. After neutralization, DNA fragments were visualized by ethidium bromide staining. Vector control (lanes 1–4), CaPolη (lanes 5–8), CaPolη 1 to 601 aa (lanes 9–12) and CaPolη H624A, H628A (lanes 13–16). *C*, FOA-resistant colonies were estimated for various strains of *C. albicans* as indicated as a measure of LOH upon UV exposure. *D*, microscopic images of serum-induced germ tube formation by Polη deficient cells containing WT and ubz mutants of CaRad30 constructs. *E*, cells with or without germ tube and chained cells were counted. *F*, length of germ tubes by various cells was measured.

In our earlier studies, we reported that the roles of CaPolη in genome stability, genotoxins-induced filamentation and azole drug tolerance by *C. albicans* are due to its translesion DNA synthesis activity, while its TLS-independent functions play a pivotal role in serum-induced morphogenesis and amphotericin B resistance. *C. albicans* cells harboring CaPolη with catalytically inactive mutations (D168A, E169A) exhibited similar phenotypes as the *rad30ΔΔ* strain (26, 27). To get insights into the cellular role of ubz in Polη, various *C. albicans rad30ΔΔ* strains possessing either wild-type or ubz deletion or point mutations of CaPolη expressed under the methionine regulated *MET3* promoter were subjected to UV sensitivity and *in vivo* TLS-mediated genomic stability assay (Fig. 5). The *C. albicans* strains possessing truncated CaPolη 1 to 601 aa or H624A, H628A mutations exhibited similar sensitivity as the vector control (Fig. 5*A*). Only the knockout strain expressing wild-type CaPolη survived to a higher dosage of UV irradiation (16–32 J/m^2^). Thus, like in *S. cerevisiae*, to carry out translesion DNA synthesis even in *C. albicans*, CaPolη requires both the catalytic and the ubz domains. Further these strains were exposed to sublethal dose of UV radiation and allowed to repair the damage by growing them on fresh media. Since TLS activity of Polη is required to bypass DNA lesions to prevent replication fork collapse and accumulation of DNA breaks, any strains failing to conduct TLS will accumulate fragmented smaller sized chromosomal DNA, while the ability of Polη to bypass UV-induced lesions will lead to the accumulation of longer chromosomal DNA. Alkali agarose gel electrophoresis of total genomic DNA isolated at various time points of recovery revealed that an equal amount of genomic DNA degradation was found in each cell type prior to recovery (Fig. 5*B*, lanes 1, 5, 9, and 13). The longer the duration of the recovery period, the more was the accumulation of larger DNA fragments, and a lesser amount of smaller chromosomal DNA fragments were accumulated in the cells expressing wild-type than expressing ubz domain deletion or its site-directed CaPolη mutants (lanes 2–4, 6–8, and 14–16). This result suggested that *C. albicans* expressing CaPolη was proficient in carrying out UV-inflicted DNA lesion bypass than the cells possessing Polη ubz mutants, and this could be because of the inability of the CaPolη ubz mutants to interact with CaPCNA for efficient lesion bypass. To further strengthen our result, the requirement of ubz domain of CaPolη in maintaining genomic stability was determined by estimating the loss of functionality of *URA3* gene upon 5-fluoroorotic acid (5-FOA) treatment with and without UV irradiation. First, we generated a heterozygous *URA3/ura3*-in Polη-deficient *C. albicans* strain, and the loss of heterozygosity (LOH) was estimated through a 5-FOA resistance assay (Fig. 5*C*). We found that without UV irradiation, the rate of LOH in various *rad30ΔΔ C. albicans* strains remained unaltered (5–8 per 5 × 10^5^ cells), even it was the same in wild-type Polη expressing cells when exposed to UV. However, the number of FOA-resistant colonies increased by ∼three-fold in the cells expressing ubz deletion and H624A, H628A mutants of Polη (16–25 per 5 × 10^5^ cells) upon UV exposure.

Morphogenesis plays an important role in *C. albicans* pathogenesis. Certain gene knockout strains locked in either round or hyphal structures are avirulent in developing systemic fungal infections in animal models. Filamentation in *C. albicans* is induced by elevated temperatures, serum, spider media, DNA damaging agents, and other specific nutrients (27). *C. albicans rad30Δ* or its catalytically inactive mutant strains exhibited reduced serum-induced germ tube formation (26, 27). To decipher the associated role of ubz domain in morphogenesis, germ tube development assay was carried out (Fig. 5, *D*–*F*). The auxotrophic BWP17 strain of *C. albicans* does not undergo filamentation due to the absence of functional *URA3* gene; however, an ectopic expression of *URA3* by its integration into the genome results in germ tube formation induced by serum (35). First, we treated *C. albicans rad30ΔΔ* strains harboring either wild-type or mutations in the ubz domain of CaPolη to 10% fetal bovine serum (FBS) for 1 h before checking their cellular morphology. Except for the strain expressing wild-type Polη, where 97% of *C. albicans* cells developed germ tubes, only ∼50% of cells from other ubz defective strains developed germ tubes; that also with substantially reduced lengths (Fig. 5, *E* and *F*). Consequently, more round cells were counted in *C. albicans* cells without the functional ubz domain. These results suggest that CaPolη without ubz domain exacerbates morphological defects induced upon serum addition. Taking it all together, we conclude that the ubz domain of CaPolη plays a pivotal role in TLS-mediated genome stability and morphogenesis in *C. albicans*.

### Two modes of PCNA interaction in three categories of Polη

**Figure 6:**
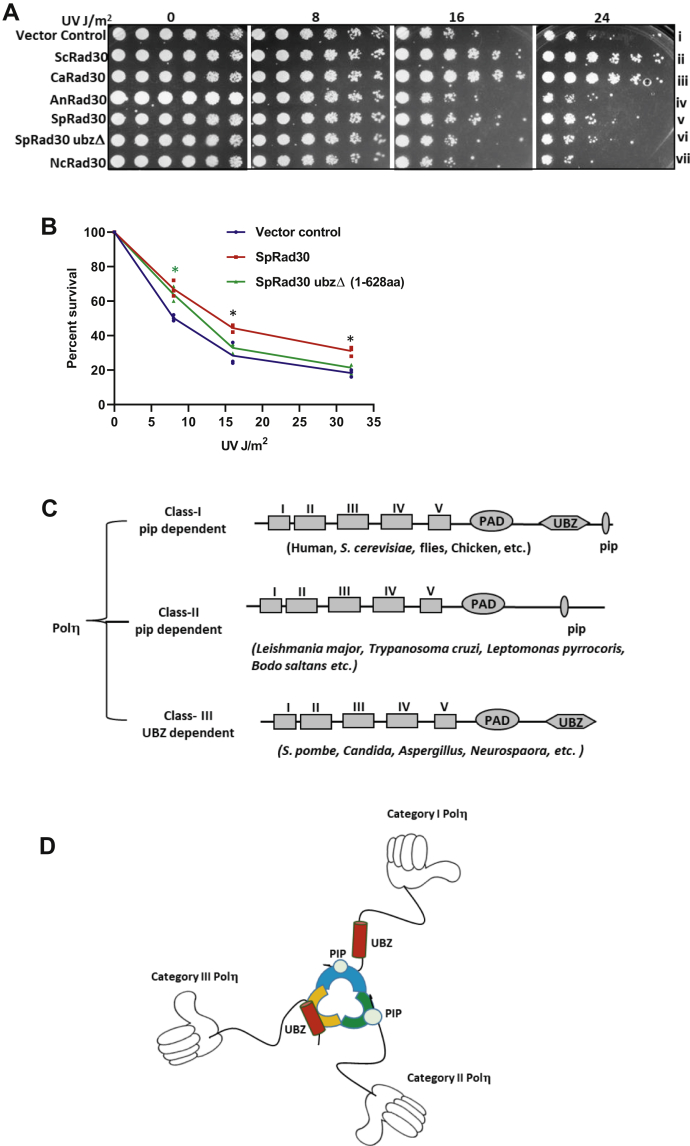
**Evolutionarily conserved role of ubz domain.***A*, the *rad30Δ S. cerevisiae* strains harboring vector alone or YEP-*ADH1*p-ScPolη or YEP-*ADH1*p-CaPolη or YEP-*ADH1*p-AnPolη or YEP-*ADH1*p-SpPolη or YEP-*ADH1*p-SpPolη ubzΔ (1–628) or YEP-*ADH*1p-NcPolη plasmids from an overnight SD-Ura culture were serially diluted and spotted onto SD-Ura plates. The culture plates were irradiated with indicated doses of UV radiation, covered with aluminum foil, incubated at 30 °C for 3 days, and then photographed. *B*, the colony formation units of Polη deficient cells expressing either full-length SpPolη or SpPolη ubzΔ (1–628 aa) when exposed to UV radiation. ∗Student *t* test *p* value, *p* ≥ 0.05 when compared between wild-type SpPolη and SpPolη ubzΔ (1–628 aa) or the vector control. ∗ Student *t* test *p* value, *p* ≥ 0.05 when compared between wild-type SpPolη and the vector control. *C*, the two proposed modes of PCNA interaction by the three categories of Polη. If pip motif is present in a given Polη, it becomes the primary site of PCNA interaction. If pip is absent, ubz of Polη becomes the primary site. *D*, a cartoon diagram of two modes of PCNA interaction mediated by either pip box or ubz domain of three categories of Polη. A trimeric ring of PCNA and each monomer has been shown in *orange*, *blue*, and *green colors*. The catalytic domain of Polη has been shown as “hand shape,” pip as a *circle*, and ubz as a *cylinder* located in the C-terminal tail.

From the above results and our earlier observations, it is evident that while *S. cerevisiae* and human Polη gain access to PCNA *via* a pip motif, CaPolη that intrinsically lacks a pip motif interacts with PCNA through its ubz domain for the TLS activity. Thus, for *S. cerevisiae* and human Polη where a pip motif is present, ubz becomes dispensable; and for *C. albicans* Polη where pip is naturally absent, the role of ubz becomes imperative. To understand the evolutionarily conserved role of the ubz domain of Polη, we selected a few other members of Polη from category III that lack canonical PCNA interaction motif at the carboxyl-terminal tail. We expressed wild-type Polηs from *S. pombe*, *Neurospora crassa*, and *Aspergillus nidulans* in *rad30Δ S. cerevisiae* strain for complementation analysis (Fig. 6*A*). Unlike CaPolη, Nc- and An-Polη failed to complement the function of ScPolη, whereas SpPolη (872 aa) partially complemented and supported the growth even at a higher dosage of UV. However, the deletion of the ubz domain of SpPolη (1–682 aa) completely abolished resistance to UV; thus, similar to CaPolη, the ubz domain of SpPolη is essential for its TLS activity (compare sectors v and vi with i). To strengthen the spot assay, we estimated the colony formation units when cells expressing either SpPolη or SpPolη ubzΔ were exposed to UV radiation. The *S. cerevisiae* cells expressing wild-type SpPolη formed more colonies upon treatment with UV than the cells expressing ubz deletion mutant of SpPolη, which is similar to a Polη-deficient strain (vector control, Fig. 6*B*). Although we could not find complementation by Nc- and An-Polηs in *rad30Δ S. cerevisiae* strain, the results obtained from ubz mutational analyses of Ca- and Sp-Polηs clearly supported the indispensable conserved role of ubz of category III Polη in TLS. Considering earlier studies and this study, we propose two modes of Polη recruitment to PCNA, mediated either by pip box or ubz domain in three categories of Polηs (Fig. 6, *C* and *D*). For category I and II Polηs, pip motif plays an essential role, and ubz/ubm, if present becomes dispensable, whereas in category III Polηs, as they lack pip motif intrinsically, ubz domain interaction with the PCNA becomes critical. While in category I, ubz may interact indirectly with PCNA mediated by the ubiquitin moiety, in category III Polηs, ubz directly binds to the IDCl of PCNA similar to the pip motif of category I and II Polηs.

## Discussion

To gain access to the replication fork, whether, during replication or translesion DNA synthesis, DNA polymerase needs to interact with PCNA, a step that is considered to be essential (1, 3). Therefore, cells harboring a DNA polymerase that is either catalytically inactive or defective in PCNA binding or completely deficient in that specific DNA polymerase functions exhibit exactly similar phenotypes. In that context, all the DNA polymerases must possess a dedicated PCNA interaction motif or domain, which is usually found at their regulatory carboxyl-terminal portions. Interestingly, this study serendipitously identified a group of Polηs, mostly belonging to fungal species that differed from the other two categories by not possessing a canonical pip motif in their extreme C-terminal tails. Therefore, this study was designed to decipher the underlying mechanism of PCNA interaction by this new set of Polηs belonging to category III and to propose possible modes of PCNA interaction that exist in these three categories of Polηs.

Although the multiple sequence alignment suggested lack of a pip motif in category III Polηs, by mutational analyses we first ensured the absence of any such pip motif in CaPolη, a member from category III. Further, by carrying out extensive genetic and biochemical analyses of various CaPolη mutants, we revealed that the ubz domain is necessary and sufficient for both physical and functional interaction with PCNA. Additionally, although Polη from *S. pombe* partially complemented ScPolη, analyses of its ubz mutant further supported the significance of this domain in TLS function. Thus, we proposed that where pip motif is naturally absent, the ubz domain of Polηs could play a critical role in PCNA interaction. In contrast to category III Polηs, the representative studies on Polηs from categories I and II suggested the indispensable nature of the pip motif in PCNA interaction and for their TLS activities, thereby the existence of two distinct modes of PCNA interactions among Polηs mediated either by pip or ubz domains is possible (8, 17, 18, 24). While ubz is present in category I, our multiple sequence alignment of plant Polηs suggested an absence of ubz domain in category II Polηs. However, an earlier report predicted the presence of ubm-like motifs in AtPolη, although these motifs hardly show any similarity with the other identified ubms in Polκ, Polι, and Rev1 (12, 24). Ubms that do not coordinate zinc are usually absent in Polηs; therefore, whether so-called ubm-like motifs in AtPolη can even bind to ubiquitin requires further investigation (Fig. S1*B*, ii). Moreover, Polηs from protists such as *Trypanosoma*, *Leishmania*, *Caenorhabditis elegans* of category II also lack ubz completely, but they have pip-like motifs at the C-terminal domain. Since the mutations in the C_2_H_2_ motif of ScPolη and HsPolη exhibited either no or weak phenotypes when compared with their pip mutants and ubz is absent in category II Polηs, the role of ubz becomes dispensable for the TLS function in category I and II Polηs (8, 17, 18). This discrepancy of the role of ubz among Polηs posits two pertinent issues: (1) why does the ubz domain of category I Polη fail to bind to the IDCL of PCNA, and (2) how does ubz domain of category III Polη interact with the IDCL of PCNA? An earlier study reported that the binding affinity of pip peptide sequences from ScPolη and HsPolη with their respective PCNAs ranges from 1.6 to 11 μM (36), which is somewhat similar to the binding affinity between ubz peptide and PCNA (∼15 μM) but about ten-fold more than that between ubiquitin and ubz of Polη (∼80 μM) (32, 37, 38). Such a high affinity between pip of DNA polymerase and PCNA is ideal and needed for efficient and processive DNA synthesis (4). Therefore, a pip motif of Polη may be the preferred site of interaction over the indirect ubz binding to PCNA *via* ubiquitin for a stable binding and efficient DNA synthesis. Even though the binding affinity of the pip and UBZ peptide sequences to the IDCL region of PCNA is fairly similar, it could be possible that in the context of a full-length Polη protein, the binding affinity of the pip to PCNA might be greater than that of the UBZ domain. In fact, we find the binding affinity of full-length CaPolη toward PCNA is at least two-fold higher than that of the ubz peptide sequence; it also signifies the importance of the neighboring residues surrounding these motifs in PCNA binding as well. Moreover, our analyses of hybrid Polη constructs derived from the fusion of various C-terminal regions of Sc- and Ca-Polη yet again suggested the importance of neighboring residues close to pip or ubz domain to have a decisive role in their PCNA interaction. Thus, it could be possible that in category I Polηs, the pip motif preferably binds to the IDCL with high affinity, which in turn leads to a conformational change at the C-terminal domain to preclude binding of ubz/ubm to the IDCL of PCNA; however, the binding of ubm/ubz to the ubiquitin moiety of ub-PCNA still can occur, although such interaction is not a prerequisite and nonessential for TLS activity. However, in category III Polη such as in Ca- and Sp-Polηs since the pip motif is absent, the ubz domain could directly access the IDCL of PCNA. This is also supported by the fact that the fusion of the C-terminal domain of CaPolη to the catalytic core of ScPolη led to a reduced UV sensitivity of the *S. cerevisiae rad30Δ* strain (Fig. 4*B*, sector xiv). Thus, even in *S. cerevisiae* Polη, the ubz domain can bind to PCNA provided the pip is completely absent in the C-terminal domain. Unlike the pip motif that forms a flexible 3_10_ helix and gets stabilized into the hydrophobic pocket of the IDCL in PCNA trimer, ubz is structurally more organized, consisting of two antiparallel β-strands and an α helix (4, 32). Since the D626A CaPolη mutant did not bind to PCNA and failed to suppress the UV sensitivity of *S. cerevisiae rad30Δ* strain, the α-helix of CaPolη may be the site of PCNA interaction. However, the precise mode of binding of the α-helix of ubz of CaPolη to the IDCL of PCNA requires further structural investigation. Nevertheless, several cellular proteins such as ubiquitin, PD1P38, Rad18, and WRNIP1/Mgs1 were found to be binding to the ubz of Polη (32, 39, 40, 41). Thus, ubz is undoubtedly a protein–protein interaction module, and our mutational analysis of Ca-and Sp-Polη evidently supported the certain species-specific direct role of the ubz domain of Polη in PCNA interaction.

Additionally, this study finds the critical role of the ubz domain of CaPolη in other cellular activities. *C. albicans* is a human pathobiont that exists in several morphological forms and causes superficial to invasive systemic fungal infections in immune-suppressed individuals. Switching its morphology from oval-shaped to pseudohyphal to hyphal structures in response to environmental niche is considered to be one of the virulence determinants of *C. albicans*. In our earlier reports, we found the role of catalytic activity of CaPolη in genome stability, filamentation, and antifungal drugs resistance in *C. albicans* (25, 26, 27, 28). Herein, we found the ubz domain of CaPolη playing a similarly important role as the catalytic domain of CaPolη in TLS, LOH, and germ tube formation. Since ubz peptide-PCNA mixture failed to show any interaction with Polη, blocking of PCNA-CaPolη interaction either by ubz peptide or any other similar small molecule targeting IDCL of CaPCNA can be explored further for translational implications.

This is the first report to provide conclusive evidence to suggest the essential and direct role of the ubz domain of Polη in PCNA interaction, in TLS, as well as in other cellular activities. Most probably, it is the position of the ubz domain that determines whether it will interact directly or *via* ubiquitin to PCNA. Nevertheless, both will make a topological link of Polη to the chromosomal DNA. This study not only has settled a long-standing controversy over the function of the ubz domain of Polη but also revealed two modes of PCNA interaction by the various groups of Polη. We conclude that the ubz domain is not a mere protein–protein interaction domain; rather, it is an essential regulatory domain at least in *C. albicans* Polη and possibly in other category III Polηs, which can be targeted to develop therapeutics against pathogenic fungi.

## Experimental procedures

### Oligonucleotides, peptides, strains, and growth conditions

The oligonucleotides used in this study were procured from Integrated DNA Technologies (IDT). Ubz and its mutant peptides were synthesized with 98% purity and procured from China peptides Co Ltd. The wild-type strains of *S. cerevisiae* EMY74.7 and *C. albicans* SC5314 and BWP17 and their derivatives used for the study are given in Table 3. *C. albicans* and *S. cerevisiae* strains were grown in YPD media with or without DNA damaging agents and on various synthetic dropout media as required.

**Table: 3:** List of *S. cerevisiae* and *C. albicans* strains used in the study

Strain	Genotype	Source/Reference
EMY74.7 (*S. cerevisiae*)	MATa his3-Δ1 leu2–3 leu2–112 trp1Δ ura3–52 (derived from DBY747: his3-Δ1 leu2–3 leu2–112 ura3–52 a)	(17)
YR30.2	EMY74.7 *rad30Δ*	(17)
YR30.13	EMY74.7 *rad30Δ rev3Δ*	(35)
SC5314	Wild-type *C. albicans*	(26)
BWP17 (*C. albicans*)	*ura3::imm434/ura3::imm434,iro1/iro1::imm434,his1::hisG/his1::hisG. arg4/arg4*	(35)
CNA11	BWP17 *rad30ΔΔ*	This study
CNA26	BWP17 *rad30ΔΔ*, *MET3p-URA3*	This study
CNA27	BWP17 *rad30ΔΔ*, *MET3p-CaRad30-URA3*	This study
CNA28	BWP17 *rad30ΔΔ*, *MET3p-CaRad30 1–601 aa-URA3*	This study
CNA35	BWP17 *rad30ΔΔ*, *MET3p-CaRad30 H624A,H628A-URA3*	This study

### Generation of Rad30 constructs

The cloning strategy for CaRad30 and ScRad30 has already been described (25, 26). A similar Pfx DNA polymerase-based PCR approach was used for the cloning of other fungal Rad30s. A 30-cycle PCR reaction was carried out using primers NAP349 (5′-CCG GGG ATC CAC ATA TGC CGC TCT CCC CAG AAC C-3′) and NAP350 (5′-GGC CGG ATC CTC ATC CAA ACG TAA GTC G-3′) for AnRad30; and NAP343 (5′-CCG GGG ATC CAC ATA TGG AAT TAG GCA AAA GC-3′) and NAP344 (5′-GGC CGG ATC CTC AAC TTT CAT AAA CAG CAT ATC G-3′) for SpRad30. As *N. crassa* Rad30 contains a single intron within the orf, using its cDNA as a template and NAP438 (5′-CCG GGG ATC CAC ATA TGT CGT CAC CGC CC-3′) and NAP347 (5′-CCG GGG ATC CTC AAC CGA ACT TGA GTT TGC -3′) primers NcPolη orf was amplified. To generate truncated Rad30 orfs, NAP03 and NAP08 (5′-CCG GGG TAC CGG ATC CTC ATT ACA AAA GGG TAA ATC TGT C-3′) for CaRad30 (1–512); and NAP343 and NAP345 (5′- CCG GGG ATC CTC ACT TTT TCC AAT TTG GTG TCA GAT TTC C) for SpRad30 (1–628) primers were used for amplification. In the case of CaRad30 (1–601) truncation, an SMURFT linker was ligated into the unique XbaI site of the CaRad30 orf construct. Similarly, by using NAP03 and a set of reverse primers containing either D626A mutation in NAP333 (rp 5′- CCG GGG ATC CTT AAT GAT TAT TTA GTT TGT TTG ATA AGT CCA TCG CTA TGT GGT AGG CGT TGT GCT C-3′) or H624A, H628A mutation in NAP334 (rp 5′-CCG GGG ATC CTT AAT GAT TAT TTA GTT TGT TTG ATA AGT CCA TCG CTA TGG CGTA ATC GTT GGC CTC TAC AGG-3′) ubz mutations in CaRad30 were generated. But inverse PCR reaction was carried out by using primers NAP365 (5′-GCT CTT CAA GAG GCT GCA GAC TAT GCT TTA GCA TTG-3′) and NAP366 (5′-CAA TGC TAA AGC ATA GTC TGC AGC CTC TTG AAG AGC-3′) for generating ScRad30 with H568A, H572A mutations. CaRad30 F485A, L486A mutant was generated by replacing 1 to 1464 bp wild-type fragment with PCR product amplified using primers NAP03 and NAP231 (5′-GTG TTG AAT TCC GCG GCC TCT CTT AAT AAG ACA CAG-3′) by digesting with KpnI and EcoRI. All these amplified PCR products were purified, digested with BamHI, and cloned into a similar site in a 2μ-based vector with *S. cerevisiae ADH1* promoter (29). For protein expression and purification, BamHI fragments of wild-type and mutated Rad30 orfs were cloned as amino-terminal GST fusion into the BglII site of pNA680, which is under the control of the *GAL-PGK* promoter. These constructs were confirmed by DNA sequencing.

### Generation of Rad30 chimeric constructs

Three CaRad30 chimeric constructs were generated that retained the CaPolη sequences with or without ubz mutations but the pip sequence taken from ScRad30 was fused to their last amino acid. For fusing ScRad30 pip sequence, PCR was performed either on wild-type or ubz mutants templates by using primers NAP03 and NAP338 (rp 5′-GGC GGG ATC CTC ATT TTT TTC TTG TAA AAA ATG ATA AGA TGT TTT TGG AAG ATG TAA CTT GTT TCT TTT TTA GAG-3′). Similarly, the ubz sequence from CaRad30 was fused to ScRad30 lacking the complete C-terminal domain including its own ubz and pip motif. PCR was performed with Q5 high-fidelity DNA polymerase using primers NAP286 (fp 5′- GGC CGG ATC CGT ATG TCA AAA TTT ACT TGG-3′) and NAP368 (rp 5′-GGC CGG ATC CTT AAT GAT TAT TTA GTT TGT TTG ATA AGT CCA TCG CTA TGT GGT AAT CGT TGT GCT CTA CAG GAT CGT CTA CAC TAA GTT TGC ATC TCG AAC ATT CCA ACT TCG G-3′) on *S. cerevisiae* Rad30 template. The PCR products were cloned into the BamHI site of a 2μ-based vector with *ADH1* promoter for complementation analysis. The fusions were confirmed by DNA sequencing.

### Generation of CaPCNA mutant constructs

Inverse PCR was carried out using the primer set NAP224 (5′-GTG AAT ATT CTT TAA AAT TAA TGG ATA TTG ATT CTG AAT TTG CAC AAG CTG ATG ATA TGG-3′)-NAP291 (5′-CCA TAT CAT CAG CTT GTG CAA ATT CAG AAT C-3′) on pUC19-CaPCNA to generate CaPCNA-79 (L126, 128AA). CaPCNA-90 (P253A, K254A) was generated by swapping the mutated PCR product amplified using primers NAP31 (5′-GGC CAA GCT TGG ATC CAC ATA TGT TAG AAG GTA AAT TTG AAG-3′) and NAP225 (5′-G GCC GAA TTC GGA TCC CTA CTC ATC ATC ATC GAA TGC TGC TGC CAA G-3′) in pUC19-CaPCNA template. The PCNA mutated orfs were further subcloned to generate bacterial expression systems either with amino-terminal GST or carboxyl-terminal His-tagged CaPCNA mutants, similar to CaPCNA expression constructs (29, 34).

### Generation of BWP17 homozygous *rad30ΔΔ* strain and expression of various mutants of CaPolη

The BWP17 *C. albicans* strain is an auxotrophic isogenic derivative of SC5314. Deletion of the *RAD30* gene in BWP17 was carried out as in the SC5314 strain of *C. albicans* (26). Briefly, two *RAD30* deletion cassettes with a nourseothricin marker (pNA1389 and pNA1451) were generated, containing different lengths of upstream sequences but the same downstream gene sequence to facilitate an efficient homozygous knockout. Two successive rounds of transformation, followed by curing of the nourseothricin marker by maltose, resulted in the homozygous deletion of *C. albicans* strain CNA11. To express wild-type CaRad30, CaRad30 (1–601), and CaRad30 H624A, H628A mutants under repressible *MET3* promoter, various constructs were integrated into the RP10 locus of CNA11 strain, and the transformants were selected by *URA3* marker, and their expression was induced in the absence of methionine in synthetic dropout media.

### Bioinformatics analysis

Seventy-seven Polη amino acid sequences from various organisms were taken from the NCBI database and aligned using the t-coffee multiple alignment tool. Further, they were segregated into three groups based on homology and presence or absence of critical pip/ubz motifs and again aligned to get final alignments. The amino acid sequence of *C. albicans* Polη was aligned with that of ScPolη by using pairwise sequence alignment tool, EMBOSS Needle (http://www.ebi.ac.uk/Tools/psa/emboss_needle/) to map the pip motif. Ubz model structures for CaRad30 and ScRad30 were generated by using FFAS (http://ffas.burnham.org/ffas-cgi/cgi/ffas.pl) online modeling database. The ubz domain of HsPolη with PDB ID 3WUP was used as a template for modeling. Secondary structure information was derived by observing the generated model.

### Purification of recombinant proteins

PCNA protein expression was carried out in *E. coli* BL21-DE3 strain as described earlier (33, 34), whereas wild-type and truncated CaPolη was purified in YRP654 protease deficient *S. cerevisiae* strain (42).

### Physical interaction by ITC

All the purified proteins and purchased peptides were dialyzed overnight against 1 l of a buffer containing 20 mM HEPES (pH 7.4) and 150 mM NaCl at 4 °C to ensure complete removal of DTT and glycerol from the protein storage buffer, which could affect the heat exchange while they interact. ITC assays were performed using a Malvern MicroCal PEAQ-ITC calorimeter. Before the experiment, the cell and the syringe were thoroughly washed with water, followed by cell rinsing with a buffer. A control run was carried out to make sure that the buffer is not participating in heat change where the cell was filled with 300 μl of a buffer and concentrated CaPCNA or ScPCNA protein (200 μM) in the syringe. ITC was performed using various Polηs or UBZ peptide or UBZ mutant peptide in the sample cell and CaPCNA or ScPCNA or a mixture of CaPCNA+ WT UBZ peptide in the syringe. Twenty-five injections of 1.5 μl of protein from the syringe were given at intervals of 120 s with an initial delay of 120 s at 25 °C. The data were analyzed to determine the various kinetic parameters using a single-site binding model provided in the ITC analysis software package. The experiments were repeated twice for the positive interactions, whereas for the negative interactions, those were repeated multiple times at various concentrations of protein or peptide in the cell to confirm that there is no binding.

### GST pull-down assay

GST-fused wild-type or mutant Polη proteins bound to glutathione sepharose beads were mixed with 0.5 μg of His-tagged wild-type or mutant CaPCNA, and a pull-down experiment was carried out using a standardized protocol, described previously (43). Then the beads were thoroughly washed thrice with ten volumes of equilibration buffer (50 mM Tris-HCl, pH 7.5, 150 mM NaCl, 5 mM dithiothreitol, 0.01% NP-40, and 10% glycerol). Finally, the bound proteins were eluted with a 50 μl SDS loading buffer. Various fractions were resolved on a 12% SDS-PAGE, followed by Western blot using an anti-His antibody (Catalog no. H-1029, Sigma-Aldrich) in 1:1000 dilution. After washing thrice, the blot was incubated with an anti-mouse secondary antibody (Catalog no. A-9044, Sigma-Aldrich). Then it was developed after three washes.

### Size-exclusion chromatography

For size-exclusion chromatography, about 10 μg of purified proteins (CaPCNA and CaPolη) was incubated together at room temperature for 60 min, loaded onto a Superdex 200 PC3.2/30 column preequilibrated with a buffer containing 50 mM HEPES, pH 7.5, 150 mM NaCl and 10% glycerol. Chromatography was performed on an AKTA pure M system (GE Healthcare) at a flow rate of 0.05 ml/min at 4 °C, and the absorbance was monitored at 280 nm.

### UV sensitivity and UV mutagenesis

The *rad30Δ* and *rad30Δ rev3Δ* genomic knockout strains of *S. cerevisiae* harboring either wild-type or various mutant Rad30s were grown in dropout medium lacking Uracil (SD-Ura) to maintain selection for the plasmid. Knockout strains with empty vector YEplac195-Sc*ADH1*p (2μ, URA3) were also grown as a control. Cultures were grown up to the mid-logarithmic phase, washed with water, and resuspended to a density of 2 × 10^8^ cells/ml. Desired dilutions of cells were spread onto the surface of SD-Ura plates for viability determination and onto SD-Arg plates containing canavanine for mutagenesis assays (17). UV irradiation was carried out with different doses of UV. After UV irradiation, the plates were incubated in the dark, and colonies were counted after 3 days.

### Filamentation test

Overnight grown culture of the different strains of *C. albicans* cells was inoculated in a synthetic dropout medium lacking methionine but containing 10% FBS and incubated at 37 °C for 1 h. Cells were observed under a Leica microscope with 40× magnification. A total number of 100 cells were counted for each strain to calculate the percentage of germ tubes and one-three chained cells in three independent experiments. Similarly, the measurement of germ tubes (n = 23) length was performed for each strain using ImageJ software. All the graphs were made using the GraphPad Prism 8.0 software.

### Alkaline agarose gel electrophoresis

The total genomic DNA was isolated from different strains of *C. albicans* and resolved by alkali agarose gel electrophoresis as described earlier (44). Briefly, an equal number of cells were exposed to 16 J/m^2^ UV, and further, they were allowed to recover in fresh media. After subsequent recovery at various time points 0, 6, 12, and 24 h, cells were harvested. Cells were lysed by glass beads and genomic DNA was extracted by removing the impurities by phenol: chloroform: isoamyl alcohol treatment. A 0.7% agarose gel was prepared in 50 mM NaCl and 4 mM EDTA, and the gel was presoaked in alkaline electrophoresis buffer (30 mM NaOH and 2 mM EDTA) overnight at room temperature prior to running. An equal volume of DNA was loaded for each sample into the wells and allowed to separate at 0.25 V/cm until the dye migrated to 60% of the length of the gel. Agarose gel was neutralized in 500 ml of 0.5 M Tris-HCl, pH 8.0 at room temperature for 1 h, and finally, the gel was stained with ethidium bromide, and the image was captured in a Chemidoc Imaging system (Bio-Rad).

### 5′ FOA-mediated loss of heterozygosity assay

*C. albicans* cells expressing different forms of Polη were grown in nonselective liquid medium (YPDU) to an OD_600_ of ∼1, and the serial dilutions of their cells (10^5^, 5 × 10^4^, 10^4^, etc.) were spread onto SD+5′- FOA (1 μg/μl) and YPDU plates. After plating, one of the sets of plates was exposed to 16 J/m^2^ UV and wrapped in aluminum foil. All the plates were incubated for 3 to 4 days at 30 °C. FOA-resistant colonies were counted, and some of the resistant colonies were picked up and restreaked on SD-Ura, SD-Arg, and SD-His plates to ascertain the genotypes.

## Data availability

All data presented is contained within this manuscript.

## Supporting information

This article contains supporting information.

## Conflict of interest

The authors have no conflicts of interest to declare regarding the publication of this article.
