# Supplementary material for: The ubiquitin-binding domain of DNA polymerase η directly binds to DNA clamp PCNA and regulates translesion DNA synthesis
Source: J Biol Chem. 2021 Dec 18;298(2):101506. doi: 10.1016/j.jbc.2021.101506 (PMC8784325; doi:10.1016/j.jbc.2021.101506)
Supplement: Supplemental Figures S1–S3 [file mmc1.pdf]

Supporting informations

The ubiquitin-binding domain of DNA polymerase η directly binds to DNA clamp PCNA and regulates translesion DNA synthesis

Kodavati Manohar[1,3,+], Prashant Khandagale[1,4,+], Shraddheya Kumar Patel[1,2,+], Jugal Kishor Sahu[1,2], and Narottam Acharya[1,*]

[1]Laboratory of Genomic Instability and Diseases, Department of Infectious Disease Biology, Institute of Life Sciences, Bhubaneswar-751023, India.

[2]Regional Centre for Biotechnology, Faridabad, India.

[3]Present address: Department of Neurosurgery, Center for Neuroregeneration, Houston Methodist Research Institute, Houston, TX 77030, USA.

[4]Present address: Developmental Therapeutics Branch, Center for Cancer Research, NCI, NIH, Bethesda, Maryland 20892, USA

*Correspondence to:

Narottam Acharya, Phone: 91-674-2304278, Fax: 91-674-2300728

E-mail: narottam_acharya@ils.res.in; narottam74@gmail.com

[+]contributed equally.

Legends for supporting Figures

Fig. S1: Classification of three categories of Rad30 sequences from various organisms across kingdom. A. Category I Rad30s containing both ubz domain and pip motif(s). **B**. Category II Rad30s containing only pip motif. For better alignment Rad30 sequences from protists (i) and plants (ii) have been separated. **C.** Category III Rad30s containing only ubz domain. Critical motifs in the catalytic domains are shown in purple (Motif I to V), pip motifs in green and ubz domains in brown colours.

Fig. S2 A control experiment where buffer was injected to PCNA in the cell and binding was analysed by ITC.

Fig. S3: (A) GST pull-down of His-PCNA mutants by CaPolη. Beads of GST-CaPol was mixed with CaPCNA (lanes 1-3) or CaPCNA-79 mutant (lanes 4-6) in equilibration buffer and after incubation, the beads were washed and the bound PCNA was eluted with protein loading dye. The different fractions were resolved in 12 % SDS PAGE, blotted to the membrane and developed by the anti-His antibody. Lanes 1 and 4 are 10 % of the load; Lanes 2 and 5 are 10 % of the third washings; Lanes 3 and 6 are the total eluates. **(B)** Cells of genomic *rad30Δrev3Δ* yeast strains harboring vector alone (YEP-*ADH1*p) or ScPolη or ScPolη pip (F627A,F628A) mutant or ScPolη H568A,H572A or CaPolη 1-512 aa or CaPolη 1-601aa or CaPolη D626A or CaPolη H624A,H628A or CaPolη or CaPolη~ScRad30pip or CaPolη H624A,H628A~ScRad3 pip or CaPolη D627A~ScRad30pip or CaPolη UBZΔ~ScRad30pip or ScPolη CTDΔ~CaRad30ubz plasmids from an overnight SD-Ura culture were serially diluted and spotted onto SD-Ura plates. The culture plates were irradiated with the indicated doses of UV radiation, covered with aluminum foil, incubated at 30°C for 3 days and then photographed.

Fig. S1A: Category I DNA polymerase eta: DNA polymerase eta from fungi, animals and plant containing both UBZ and PCNA interaction motifs at the carboxyl terminal domain

```
CLUSTAL W (1.83) multiple sequence alignment

ScRad30       -----------------------------------------------------------------------------------------MSKFTWKELIQ
KlRad30       -----------------------------------------------------------------------------------------MSKYRWRDLID
CgRad30       ---------------------------------------------------------------------------------------MGHSKFTWKDLID
CcRad30(1)    ---------M-----------------------------------------------------ATSASIWKGKGKAATSD---SDFTDLNPTITYRHLLS
CnRad30       ------------------------------------------------------------------MSISVGSSRKKEGR----------VVPTYRHLLS
ClRad30       ---------M---------------------DPRSP---------------------------TRPTALWKGKAKATEDS---EDFNDLNPVITYRHAVA
CmRad30       ---------METS------------------LPDSP---------------------------TKTGSLWKGKAKDVTSA--QDEFGDLNPIITYRHLLS
SsRad30(1)    ---------M------------------------------------------------------NSP--WKGKAK--AT---NQDYDDLRPTITYRHLLS
TtRad30       ---------MWFSLAVTRFLWDKVTWERKRFKRNSPSRGLVLTPQRLFLIYKTHSRPRRLSLRLAFMWKGKSAVTDSD---SEFVDLNPTITYRHLLS
PpRad30(1)    ---------MT----------------------ESP---------------------------RKTP--WKGKAKAPPSAIEHDHFDDLNPVITYKHLLS
HsRad30(1)    ---------MATG---------------------------------------------------------------------------------------
GgRad30(1)    ---------MSRG---------------------------------------------------------------------------------------
CdRad30       ---------MANG---------------------------------------------------------------------------------------
AmRad30       ---------MAQG---------------------------------------------------------------------------------------
HmRad30       ----------------------------------------------------------------------------------------------------
JjRad30       ---------MATG---------------------------------------------------------------------------------------
EeRad30       ----------------------------------------------------------------------------------------------------
LhRad30       ---------MDFG---------------------------------------------------------------------------------------
EgRad30(1)    ----------------------------------------------------------------------------------------------------
SvRad30       ---------MSRG---------------------------------------------------------------------------------------
PbRad30       ---------MGWG---------------------------------------------------------------------------------------
PmRad30(1)    ---------MSRG---------------------------------------------------------------------------------------
DmRad30       MSSARSHVSMQNK---------------------------------------------------------------------------------------
RnRad30       ---------MAPG---------------------------------------------------------------------------------------
BtRad30       ---------MTDG---------------------------------------------------------------------------------------
DrRad30       ---------MDFG---------------------------------------------------------------------------------------
MmRad30(1)    ---------MAPG---------------------------------------------------------------------------------------
PtRad30       ---------MATG---------------------------------------------------------------------------------------
GgRad30(1)    ---------MSRG---------------------------------------------------------------------------------------
XlRad30       ---------MESG---------------------------------------------------------------------------------------
SsRad30(1)    ---------MDYG---------------------------------------------------------------------------------------
AoRad30       ---------MEYG---------------------------------------------------------------------------------------
PnRad30       ---------MLEGS--------------------------------------------------------------------------------------

ScRad30       LGSPSKAYESSLACIAHIDMNAFFAQVEQMRCGLSKEDPVVCVQWNS-------------IIAVSYAARKYGISRMDTIQEALKKCSNLIPIHTAVFKKG
KlRad30       LNDKEKSFLSPLACIAHIDVNAFFAQVEQVRCGFSRDDPVVAVQWTS-------------ILAVSYAARKYNVSRMESILDAIKKCDKIIPIHTAVFRKG
CgRad30       LNNKDKAYLSNLACLAHIDVNAFFAQAEQIRCGYSKDDPVVCVQWKS-------------IIAISYAARKHNISRMHTIQEALKKSSDIIPIHTAVFKKG
CcRad30(1)    ---QNLGVRDPLRVVGLCDSDAFYAACEMVRLGVDKDTPLVVLQWDS-------------LIAVNYPARKYGISRMDKKKDALKRCPHLKVVHVATYKEG
CnRad30       L--QALTPANPLRTIAHCDIDAAYAQFEQVRLGLPDDIPLICAQWQS-------------IIAVNYPARKYGIKRFTSIEDAKKMCPHLRIQHVATYRNG
ClRad30       ---QNLGIRDPLRVVALCDSDAFYAACEMVRLGVDKETPLVVLQWEM-------------LIAVNYPARKFGISRMDKLKDAKKRCPHLKVVHVATYKEG
CmRad30       ---QNLGVRDPLRVVALCDSDAFYAACEMVRLGVDKDTPLVVLQWDS-------------IIAVNYPARKYGISRMDKKMDALKRCPHLKIVHVATYREG
SsRad30(1)    ---NNLGVRDPLRVVALCDSDAFYAACERIRLELDPSVPIVVQQWES-------------LIAVSYPAREFGISRMDKIKDAKKKCPNLLAVHVATYKEG
TtRad30       ---HALGVKDPLRVIALCDSDAFYAGCEMVRLGVDPEQPLVVSQWDS-------------IIAVNYPSRKFGITRMDKVKDAQKRCPNLVVVHVATYKEG
PpRad30(1)    ---QNLGVRDPLRVVALCDSDAFYAACEMVRLGVDKETPLVVLQWDS-------------LIAVNYPARKYGVSRMDKKKDALNRCPHLQVVHVATYKEG
HsRad30(1)    ----------QDRVVALVDMDCFFVQVEQRQNPHLRNKPCAVVQYKSWKGGG--------IIAVSYEARAFGVTRSMWADDAKKLCPDLLLAQVRESRG-
GgRad30(1)    ----------RERVVALVDMDCFFMQVEQRFDPRLRGRPCAVVQYNQWQGGG--------IIAVSYEARAFGVSRGMWATEARALCPELLLARVPEARG-
CdRad30       ----------QDRVVALVDMDCFFVQVEQRQNPHLRNKPCAVVQYKSWKGGG--------IVAVSYEARAFGVARNMWADDAKKLCPDLLLAQVRESRG-
AmRad30       ----------QERVVALADMDCFFMQVEQRLEPRLRGRPCAVVQYNRWRGGG--------VIAVSYEARAFGVARGMWAADARKLCPDLLVARVPQARG-
HmRad30       ----------MDRIVLLIDMDCFYVQVEQKLRPETIGLPCAVVQYTSLTAAA--------IIAVSYEARAKGVSRNSNGDMAKKICPDIVLMQVPTKRG-
JjRad30       ----------QERVVALVDMDCFFVQVEQRQNPHLRNKPCAVVQYKTWKGGG-------------YVSLG--------------------------SRG-
EeRad30       -------------------MDCFFVQVEQRQNPHLRNKPCAVVQYKTWKGGG--------IIAVSYEARAFGVTRNMWADDAKKLCPDLLLAQVRE----
LhRad30       ----------KERVVALVDMDCFYVQVEQRLNPELKNKPCVVAQYKTWKGGGPVMCFVSIIAVSYEARAHGVTRNMWADDAKKLCPDLQVARVRESHG-
EgRad30(1)    ----------MDRITLLIDMDCFYVQVEQKVRPETVGLPCAVIQYTSLAAAS--------LIAVSYEAREKGVTRSMNGDAAKNQCPDLLLFQVPMRRG-
SvRad30       ----------RERVVALVDMDCFFMQVEQRLDPQLRGRPCAVVQYSEWQGGG--------VIAVSYEARAFGVSRGMWASEARALCPELALARVPQARG-
PbRad30       ----------QERVVALADMDCFFVQVEQRLDPGLRGKPCVVVQYKTWKGGG--------IIAVSYEARAFGVKRNMWAADAKRLCPDLQLARVPEARG-
PmRad30(1)    ----------RERVVALVDMDCFFMQVEQRLDPQLRGRPCAVVQYTEWQGGG--------IIAVSYEARAFGVSRGMWASEARALCPELALARVPQARG-
DmRad30       ----------YDRVVLLVDMDCFFCQVEEKQHPEYRNRPLAVVQYNPWRGGG--------IIAVNYAARAKGVTRHMRGDEAKDLCPEIVLCQVPNIRE-
RnRad30       ----------QDLVVALVDMDCFFVQVEQRQNPHLRNKPCAVVQYKSWKGGG--------IIAVSYEARAFGVTRNMWADDAKKLCPDLLLAQVRESRG-
BtRad30       ----------RDRVVALVDMDCFFVQVEQRQNPHLRNKPCAVVQYTSWKGGG--------IVAVSYEARAFGVTRNMWADDAKKLCPDLLLAQIRELHG-
DrRad30       ----------KERVVALVDMDCFYVQVEQRLKPELKNKPCVVAQYKTWKGGG--------IIAVSYEARAHGVGRNMFADDARKLCPDLQVARVPEAHG-
MmRad30(1)    ----------QNRVVALVDMDCFFVQVEQRQNPHLRNKPCAVVQYKSWKGGG--------IIAVSYEARAFGVTRNMWADDAKKLCPDLLLAQVRESRG-
PtRad30       ----------QDRVVALVDMDCFFVQVEQRENPHLKNKPCAVVQYKSWKGGG--------IVSVSYEARAFGVARNMWANDAKKLCPDLLLAQVRESRG-
GgRad30(1)    ----------RERVVALVDMDCFFMQVEQRFDPRLRGRPCAVVQYNQWQGGG--------IIAVSYEARAFGVSRGMWATEARALCPELLLARVPEARG-
XlRad30       ----------QERVVALVDMDCFYVQVEQRMNPALKNKPVVVVQYKTWKGGG--------IIAVSYEARAFGVTRNMFADDAKKLCADLQLARVREAHG-
SsRad30(2)    ----------KERVVALVDMDCFYVQVEQRLNPALKNTPCVVAQYKTWKGGG--------IIAVSYEARAHGVTRNMWADDAKK-CPDLQVARVRESHG-
AoRad30       ----------KERVVALVDMDCFYVQVEQRLNPALRNTPCVVAQYKTWKGGS--------IIAVSYEARAHGVTRNMWVDDAKTRCKDLQVARVRESHG-
PnRad30       ----------TTRVIIHLDLDCFYAQVEQRRLQIPDGQPVAVQQWGS-------------LLAVNYEARKFGVKRDTLGENRKPGQPF------DRTHQ-

ScRad30       EDFWQYHDGCGSWVQDPAKQISVEDHKVSLEPYRRESRKALKIFKSAC---DLVERASIDEVFLDLGRICFNMLMFDN-------EYELTGDLKLKDALS
KlRad30       EDYWQYHDGCGSWVEDESKKLSPTNYKVALEPYRRESRKLIKLFQDEY---DLVEKASVDEAFIELGRKLFYELLMDD-------SY---------TDFA
CgRad30       ENFWQYHDGYGSWNEDPAKQLPPEEYKVSLEPYRRESRKILKIFREFC---DHVEKASVDEVFLDLGRLCFRDLMFPN-------VEATEDDDYNIVAEN
CcRad30(1)    EKEPGYWD-----------NVDTNTHKVSLDYYRRESMKIAALFRELLP-GCEVEKASIDEAFIDFTKPVREVLLQRYPY--LAQVPP--------D-AP
CnRad30       ESEAGYWD-----------DVDPRTHKVSLDVYRRESLKILAIFKEKIP-RGEIEKASIDEAFLDLTPMVIERLLAAHPY--LSKVPE--------D-AP
ClRad30       EKEPGYWD-----------NVDTNTHKVSLDYYRRESSKIAGMFKDGLP-GCEVEKASIDEAFIDFTKPAREILLQRYPH--LAQVPP------------
CmRad30       ETEPGYWD-----------EVDTNTHKVSLDYYRRESLKIAAMFREMLP-GCEVEKASIDEAFIDFTKPVREIILQRYPH--LAQPPP--------D-AP
```

```
SsRad30(1)  EKEPGYWT-----------DIDTRTHKVSLDLYRRESNKIIS MFKEALPTGEVEKASIDEAFIDFTRPVREQLLERYPY--IAQVPV-------D-AP
TtRad30     DAEPEYWE-----------NPDTKTHKVSLDFYRRESNKIHNMFKESLPAGAELEKASIDECFIDFTRPVREELLRRYPH--LAQAPP-------G---
PpRad30(1)  EKEPGYWD-----------NVDTQTHKVSLDYYRKESLKIAALFKEQLP-GCEIEKASIDEAFFDFSKIVREVMLQRYPY--LSEVPA-----------
HsRad30(1)  --------------------------KANLTKYREASVEVMEIMSRFA----VIERASIDEAYVDLTSAVQERLQKLQ------GQPI-------S-AD
GgRad30(1)  --------------------------KADLTRYREASAEVMEVLSRFA----AIERASIDEAYLDLTGSARERLRELR------GRPL-------E-AE
CdRad30     ---------------------------KADLTKYREASREVMEIMSHFT---AIERASIDEAFVDLTSAVQERLQKLQ------GQPI-------S-AD
AmRad30     --------------------------KADLTRYREASLEVMEVMSRFA----VIERASIDEAYMDLTNAVQERLRKMR------GQPV-------P-AE
HmRad30     --------------------------KADLTKYREAGAEVIKSISKFT---SKIERASIDEAYIDVTDLLDSVAYDGGICLEKSNLVI-------D-PD
JjRad30     ----------------------------LLELYREASVEVMGVMSRFA----VIERASIDEAYIDLTSAVQERLQKLQ------GQPI-------S-AD
EeRad30     --------------------------KANLTKYREASVEVMKIMSRFA----VIERASIDEAYVDLTSAVRERLQELQ------GQPI-------S-AD
LhRad30     ---------------------------KADLTHYREASVEVIEVMSRFA---VIERASIDEAYMDLTASVQDRLKHMS------VQDI-------T-PH
EgRad30(1)  --------------------------KADLTKYRDASAEVIKSISKFT---SKIERASIDEAYIDATGINLFCVYS------TSNVVI-------D-PD
SvRad30     --------------------------KADLTRYREASAEVMQVLSRFA----AIERASIDEAYLDLTDSARHRLRELR------GRPL-------P-AA
PbRad30     --------------------------KADLSRYREASIEVMEVMSRFA----VIERASIDEAYLDLTQAVQERLQKMK------GQPI-------S-AE
PmRad30(1)  --------------------------KADLTRYREASAEVMQVLSRFA----AIERASIDEAYLDLTGSARHRLRELR------GRPL-------P-AD
DmRad30     --------------------------KADTSKYRDAGKEVANVLQRFT---QLLERASVDEAYLDITETVNHRMQQMQS----GAFAL-------Q-PQ
RnRad30     --------------------------KANLTKYREASVEVMEIMSHFA----VIERASIDEAYIDLTNAVQERLEKLQ------GQPV-------S-AD
BtRad30     --------------------------KANLTKYRDASMEVMEVMSRFA----VMERGSIDEAYLDLTSAVQERLQKLQ------NQPI-------S-AD
DrRad30     --------------------------KADLTHYREASVEVIEVMSRFA----VIERASIDEAYMDLTASVQERLKNTS------VQDI-------T-AQ
MmRad30(1)  --------------------------KANLTKYREASVEVMEIMSYFA----VIERASIDEAYIDLTSAVQERLQKLQ------GQPI-------S-AD
PtRad30     --------------------------KADLTKYREASVEVMGVMSHFS----VIERGSIDEAYIDLTSAVHERLQKLQ------GQPI-------S-AD
GgRad30(2)  --------------------------KADLTRYREASAEVMEVLSRFA----AIERASIDEAYLDLTGSARERLRELR------GRPL-------E-AE
XlRad30     --------------------------KADLTHYREASVEVMEVMSRFA----VVERASIDEAYIDLTDSVQKRLREMG------VAPI-------S-GE
SsRad30(2)  --------------------------KADLTHYREASVQVFEVMSRFA----VLERASIDEAYMDLTAAVQQRLKSMT------DQQV-------D-PQ
AoRad30     --------------------------KADLTLYREASVEVIEVMSRFA----VIERASIDEAYMDLTAAVQQRLKNMS------DEQI-------D-PH
PnRad30     --------------------------KAILRRYRVASREIFAILGSVV---SIIEKASIDEAFMDVTDMAKERLAQTTA---FSSDFC-------Q-DS
```

```
ScRad30     NIREAFIGGNYDI-----------NS-HLPLIPEKIKS-----------LKFEGDV-FNPEGRD-------LITDWDDVILALGSQVCKGIRDSIKDIL
KlRad30     DIRGIFQDGRYDL-----------ND-HLPSLPT-KLS-----------IQFSGEV-FNSQNRP-------LFEDWDDVLMCLASNSTNTIRNQIDDML
CgRad30     PLRELFVNGDYKL-----------DM-PLPPVPEALKK-----------LSYCGLV-YNHEEAP-------VIQDWDDVIFALASKNTQLIRKTIQDNL
CcRad30(1)  NGVDTPLPPPPP-ISW--Y--GTGDL-IPL---T-TGPTEGQD-QPSTS-KQQPAE-EAVHDEGLQDG-EDANTTWHDVALSIAAGMVMKARKGVLEQL
CnRad30     NGLDSPLPPPPP-IDW--S--NAGSV-FPI---D-GKEDGSGTDHQEDK-EEDERS-EDGDEFDGRTS-GSNRDSWEDWALCMGGELMSNVREEVYLRL
ClRad30     NGMDTPLPPPPP-IVW--D--ELGEL-IPI---E-HVVTDNEN-KEDSA-RNTKAP-ST----------SEAPTTWHDVMLSIAAGLMEKAREEVRVKL
CmRad30     FGVETALPPPPP-IVW--D--GKGAV-IPV---N-SPPSEDSS-ITDGQ-ISSSSD-EPGRAEPLD---KDVVSTWHDVALSIGAELMTNARDAVHTTL
SsRad30(1)  NGIDSPLPPPPP-IKW--D--DLGTV-ILP---E-EID-------------SAGEQ-SNIE--------VDGDATWHDVALSIAAELMRKIREDIHSKL
TtRad30     -AADTPLPPPPTTVTW--N----ANL-VSI---R-PKDDVANN-LQESSTSTLGDP-EP----------EDSLITWHDVALSVAVDMMQSARDQVRTKL
PpRad30(1)  SGPDTPLPDPPP-VLW--T--GLGNL-IPV---N-PPPPEPKT-GEAAECVPTDIE-VP----------ADPPTTWHDVALSIAAELMEAAREAVRVQL
HsRad30(1)  LLPSTYIEGLPQGPTT--------AE-ETV---Q-KEG-----------MRKQGLF-QWLDSLQ---I---DNLTSPDLQLTVGAVIVEEMRAAIERET
GgRad30(1)  LLPTTFVQGLPAEPGL-----------QPA---D-KEE-----------LRRRGLQ-EWLASLS---F---DNVNCPDLQLAMGAVIVEEIRVAVEKAT
CdRad30     LLPSTYIEGLPKGPMT--------AE-GTV---Q-KEE-----------LRKQGLL-QWLDSLQ---T---DNTASPDLQLTMGAVIVEEMRAAIERQT
AmRad30     LLPTTFIQGLPDDTDA--A--AAQGG-STD---C-KEE-----------LRQHGLH-QWLSSLP---F---DDSSCPDLQLTVGAVIVEEMRAAVEEVT
HmRad30     SIHIELKFSS-SSPPF--------SL-PLS-----E-------------IEGE--T-KVFDN-L-----------CDGVKLKKALFLAQRIKEQIRLDT
JjRad30     LLPTTYIEGLPRGPTA--------AE-DSV---Q-KEE-----------KRRQGLF-QWLDSLQ---V---DNASSADLQLTVGALIVEEMRAAIERQT
EeRad30     MLPTTYIEGLPQGPTT--------AK-GTV---Q-KEE-----------MRKQGLG-QWLDSLP---L---DNIASPDLQLTVGAVIVEEMRAAIERQT
LhRad30     QLRSTYIQGLPQTSD-------QSEN-ALL---D-KEE-----------RRLRGLQ-QWLECLS---S--SDRSSCAELHLTVGALIVEEMRAAVEEHT
EgRad30(1)  SMKHLYDPGDLPNPPY--------SC-SIN-----E-------------ISGQ--M-AAIEC-L-----------CDGRKLRQALCLAQKIKDQILADT
SvRad30     LLPSTFVQGLPGEPGP-----------DPG---G-KEE-----------LRQRGLD-EWLASLS---F---DNPDCPDLQLTMGAVIVEEIRVAVEAAT
PbRad30     QLGTTYIQGFPNSLEA--E------E-NTD---N-KEE-----------LQQRGVC-QWLKSLP---F---GDPSNPELQLTIGAVIMEEMRAAVESVT
PmRad30(1)  LLPSTFVQGLPGEPGPDPGGKGAHRA-DPG---G-KEE-----------LRQRGLD-EWLASLS---F---DNPDCPDLQLTMGAVIVEEIRVAVEAAT
DmRad30     ELVNTFAVGYPSIGDYV-NKITNRFANPYM---D-DER------------YQMSYDQNDLPAV-----------RQSDIRLLIGASVAGEVRAAVKKET
RnRad30     LLPSTYIEGLPRGPA---------VE-DTV---E-KED-----------IRKQGLL-QWLGSLE---K---DDPTSPDLRLTVGAVIVEEMRAAIERKT
BtRad30     LLPTTCIEGFPQGPTT--------TE-GTV---E-KEE-----------RRKQGLL-QWLDSLQ---T---ESTSSPDLQLTMGAVIVEEMRVAIEKQT
DrRad30     QLKNTHIQGFPT----------QQEN-THL---D-RDA-----------QRAAGLQ-QWLDLLS---S--STESSPADLRLAVGALIVEQMRAAVEEHT
MmRad30(1)  LLPSTYIEGLPRGPT---------VE-ETV---Q-KEA-----------IRKQGLL-QWLDSLQ---S---DDPTSPDLRLTVGAMIVEEMRAAIESKT
PtRad30     LLPTTYIEGLPQGPTT--------AE-GTD---Q-KEE-----------MRKQGLF-QWLDSLQ---T---GNTTSPDLQLTVGAVIVEEMRAAIERET
GgRad30(2)  LLPTTFVQGLPAEPGL-----------QPA---D-KEE-----------LRRRGLQ-EWLASLS---F---DNVNCPDLQLAMGAVIVEEIRVAVEKAT
XlRad30     LLKNTYVQGFPQCGM---------DR-DSL---S-KEE-----------LRRHGLE-QWLESLP---V---GDPHSADVKLAVGAIIVEEMRAAVEEET
SsRad30(2)  LLKTSHVQGYPLAPLD---QEEGAQD-TTT---D-KEE-----------RRSSGLQ-QWLSSVS-------GGSGCAELQLALGAVIVEEMRAAVEQHT
AoRad30     LLKTTYIQGYPQSSAE---LESSAED-PVL---D-KEE-----------QRSRGLL-QWLASLR---GPLSGGQSSADLQLTVGALIVEEMRTAVEKHT
PnRad30     ANHDTKVFGIDS------EDNND---------------------------EDATDKDDSLQAFP---------LTDNERLLCIGAEISREIRHAVYSKL
```

```
ScRad30     GYTTSCGLSSTKN--------------VCKLASNYKKPDAQTIVKNDCLLDFLDCGKFEITSFWTLGGVLGKELIDVLDLPHENSIKHIRETWPDNAGQ
KlRad30     GYTTSCGISMTKN--------------LSKLASNYKKPNAQTIVKNNCIDDFLDCGKFEINSFWTLGGIRGKELIDLMELPKEHSIKFIRDSWPVSSDD
CgRad30     GYTTSCGIARNKI--------------LCKLGSNYKKPDAQTVIRNNDILEFLDQGGFEITSFWTLGGALGRELEGLLNLPEKDTIKHIRETWPANHKQ
CcRad30(1)  GYSTSAGLARNKF--------------LAKLAASYRKPFGQNILRNAAIPNYLIPM--AFQKIRFLGGKLGRALAQEYDA------ATV--------GD
CnRad30     HYTCTAGIAHNKA--------------MAKLCSAWKKPNNQTILRTAAVPAFLNGR--DFTDIRSLGGKLGAAIAQQFGA------KTV--------GD
ClRad30     GYTTSAGIARNKF--------------LAKLSASYKKPNSQSILRNIAIPNYLRPL--KFQKIRFLGGKLGGALAQEYDA------STV--------GD
CmRad30     GYSTSAGIARNKF--------------LAKLTASYRKPNGQSILRNASIPNYLRPM--PFQKIRFLGGKLGRAFAEEYDA------ATV--------GD
SsRad30     GYTTSAGIARNKF--------------LAKLCASYKKPNSQTILRNRAIPDYLHPL--PFQKIRFLGGKLGDALAKEYDV------STV--------GD
TtRad30(1)  GYSTSAGIARNKF--------------LAKLTASYRKPNSQSVLRNAMIPHYLRPF--PFQKIRFLGGKLGNAVAKEYEA------STV--------GD
PpRad30     GYTTSAGIARNKF--------------LAKLTASYRKPQNQSILRNVAIPNYLKPL--EFQKIRFLGGKLGDALAKEFNA------TTV--------ED
HsRad30(1)  GFQCSAGISHNKV--------------LAKLACGLNKPNRQTLVSHGSVPQLFSQM--PIRKIRSLGGKLGASVIEILGI------EYM--------GE
GgRad30(1)  GFRCSAGISHNKM--------------LAKLACGLNKPNRQTLVSSRSVPQLFSQM--PVSSIRNLGGKLGVAITDILGV------EYI--------GE
CdRad30     GLQCSAGISHNKVKANRFQRETWISVVLAKLACGLNKPNRQTLVSHGSVPQLFSQM--PINKIRKLGGKLGAAVIEILGV------QYM--------GE
AmRad30     GYRCSAGISHNKV--------------LAKLACGLHKPNRQTLIPLGRVPQLFSQM--PINSIRNLGGKLGASVSEILGV------EYM--------GQ
HmRad30     GFQCSVGVAANRM--------------LAKLACSLNKPDKITVVPLEATQHLMNFT--KVKKVPGLGGKLGNDLIQRYGL------EYM--------GG
JjRad30     GFQCSAGISHNKV--------------LAKLACGLNKPNRQTLVSHGAVPQLFSQM--PIRKIRSLGGKLGASVIEILGV------EYM--------GE
EeRad30     SFQCSAGISHNKV--------------LAKLACGLNKPNRQTLVSHGSVPQLFSQM--PIGKIRSLGGKLGASVTEILGV------EYM--------GE
LhRad30     GFRCSAGVSHNKV--------------LAKLACGLNKPNRQTVLPLGFVPELFSTL--PIGKIRNLGGKLGSSITETLGV------ENM--------GD
EgRad30(1)  GFRCSIG-----M--------------LSKIACSLNKPDKITIVPLESVQHLMNFT--RVKKVPGLGGKLGKDLALRFNL------EFI--------GS
SvRad30     GFRCSAGISHNKT--------------LAKLACGLNKPNRQTLVSARFVPQLFSQL--PVSSIRNLGGKLGTAITDILGV------EYI--------GE
```

```
PbRad30      GFRCSVGISHNKV--------------LAKLACGLNKPNRQTLVSQGAVPQLFSKM--PISNIRNLGGKLGASIIELLGV------QYM--------GQ
PmRad30(1)   GFRCSAGISHNKT--------------LAKLACGLNKPNRQTLVSARFVPQLFSQL--PVSSIRNLGGKLGTAITDILGV------EYI--------GE
DmRad30      GYECSAGIAHNKI--------------LAKLAAGMNKPNKQTILPLTETASLFDSL--PVGKIKGLGGKFGEVVCETLGI------KFM--------GQ
RnRad30      GFQCSAGISHNKV--------------LAKLACGLNKPNRQTLVSHGSVPQLFSQM--PIRKIRSLGGKLGASVIDVLGV------EYM--------GD
BtRad30      GFQCSAGISHNKV--------------LAKLACGLNKPNRQTLVSHGSVPQLFNQV--PISKIRNLGGKLGASVIEILGV------EYM--------GE
DrRad30      GFRCSAGISHNKV--------------LAKLACGLNKPNRQTVLPLSSVPQLFSTL--PISKIRNLGGKLGSSITETLSV------ENM--------GD
MmRad30(1)   GFQCSAGISHNKV--------------LAKLACGLNKPNRQTLVSHGSVPQLFSQM--PIRKIRSLGGKLGASVIEVLGI------EYM--------GD
PtRad30      GFQCSAGISHNKV--------------LAKLACGLNKPNRQTLVSHGSVPQLFSQM--PIYKIRNLGGKLGASVIEILGV------EYM--------GE
GgRad30(2)   GFRCSAGISHNKM--------------LAKLACGLNKPNRQTLVSSRSVPQLFSQM--PVSSIRNLGGKLGVAITDILGV------EYI--------GE
XlRad30      TFQCSAGIAHNKV--------------LAKLACGLNKPNRQTILCQGSVPGLFSEL--PIGKIRHLGGKLGTSIKEILDV------EYI--------GQ
SsRad30(2)   GYRCSAGISHNKV--------------LAKLACGLNKPNRQTLLPLGSVPELFNSL--PIGKIRNLGGKLGASITETLEV------ENM--------GE
AoRad30      GFRCSAGISHNKV--------------LAKLACGLNKPNRQTILPLDSVAELFNSL--PISKIRNLGGKLGASITETLGV------QNM--------GD
PnRad30      GYTCSTGIAGNKL--------------LAKLASPLNKPNGQVVVASRFVTDLMKIL--PMRKIRGLGGKLGKQLESIYSSLDSG-VSEQ--------GE

ScRad30      --------------LKEFLDAKVKQSDYDRS-TSNIDPLKTADLAEKLFKLSRGRYG-LPLSSRP--V-VKSMMSNKNLRGK---SCNSIVDCISWLEV
KlRad30      --------------IRKFMLKKIICRDIQRSREYNINEADVAKISAKIYQLVRGQFR-LPVEPRP--L-PKSMMSKKNLRND---DCASVIDCIEWLEI
CgRad30      --------------LRDYIETELDEAENKKK-YPVVAGSKLEVLSDKLFSMVRGTFS-TPITPKP--L-IQSMMSNKNLRGK---SCNSLVDCISWLEV
CcRad30(1)   --------------LLSISLDEMQNKFGE--------------DSIWVYEILRGIDR-SEVKDKGTTL-TKSMLASKNLP-K---PITAASEGYHWIRV
CnRad30      --------------MLTVSLDEMQRKFGE--------------ESIWVYNILRGIDH-SEVTDRV--A-TKSMLASKSIR-P---AVTSPQQGHQWLSI
ClRad30      --------------LLAVGLEELQRKFGE--------------ESLWIYEVLRGVDR-TEVKDKGSTL-NKSMLASKNLP-K---PITKASEGHHWIRV
CmRad30      --------------LLSITLDEMQTKFGE--------------SSIWVYEILRGLDR-SEVKDKGSTL-NKSMLASKNLP-K---PITNVQEGYRWIRV
SsRad30      --------------LLTVSLDEIQSKFGE--------------NALWVYEFLRGIDR-TEVKEK-SAL-FKSMLASKNLP-K---PIIQPSDGHQWIRI
TtRad30      --------------LLAVSLDEFQRTFGE--------------DSIWIWEIVRGIDR-SEVKEK-PPN-SKSMGASKNLP-T---PVTMSCEGPHWIRV
PpRad30(1)   --------------LLPVSLDELQTKFGE--------------ESIWIYEVLRGIDR-NEVKDKGSVL-NKSMLASKNLP-K---PITQWSEGQHWIRV
HsRad30(1)   --------------LTQFTESQLQSHFGEK-------------NGSWLYAMCRGIEH-DPVKPRQ--L-PKTIGCSKNFPGK--TALATREQVQWWLLQ
GgRad30(1)   --------------VTKFSEMELQTHFGDK-------------TGSWLYDLCRGIDD-EPVKNRH--L-PQSIGCSKNFPGK--TALATQKEVQHWLLQ
CdRad30      --------------LTQFTESQLQSHFGEK-------------NGSWLYAMCRGIEH-DPVKPRQ--I-LKTIGCGKNFPGK--TALVTREQVQWWLLQ
AmRad30      --------------LTQFSMLQLQTHFGNK-------------TGSWLYDMCRGIEH-EPVRPRY--L-PQSIGCSKNFPGK--TALATQQQVQHWILQ
HmRad30      --------------LVNKPLSRLIEDYGDK-------------TGNWLYELCRGRDY-QSVSVRT--L-VKSIGCSKYFIGK--AALKTDEEIRHWLTS
JjRad30      --------------LTQFTEAQLQSHFGEK-------------NGSWLYAMCRGIEH-DPVKPRQ--L-PKTIGCSKNFPGK--TALATREQVQWWLLQ
EeRad30      --------------LTQFTESQLQSHFGEK-------------NGSWLYAMCRGIEH-DPVKPRQ--L-PKTIGCSKNFPGK--TALATPDQVQWWLLQ
LhRad30      --------------LTQFSKVQLEQHFGEK-------------TGPWLYDLCRGIEF-EPVKPRQ--L-PKSIGCSKNFPGK--TSLATKQQVQHWLHQ
EgRad30(1)   --------------LTAVPLPRLIEEYGEK-------------TGTWLYELCRGRDY-QAVSTRT--L-VKSIACSKNFLGK--SSLKKDAEILHWLTN
SvRad30      --------------LTQFSETELQTHFGDK-------------TGSWLYDLCRGIEE-EPVKNRY--L-PQSIGCSKNFPGK--LALATQKAVQHWLLQ
PbRad30      --------------LIQFSESQLQTHFGEK-------------TGSWLYELCRGIDY-EPVKARQ--L-PKSIGCSKNFFGK--TSLVTQKQVQYWLLQ
PmRad30(1)   --------------LTQFSETELQTHFGDK-------------TGSWLYDLCRGIEE-EPVKNRY--L-PQSIGCSKNFPGK--SALATQKAVQHWLLQ
DmRad30      --------------VVKFSEVDLQRKFDEK-------------NGTWLFNISRGIDL-EAVTPRF--Y-SKSIGCCKKFPGR--NNITGLKTLQHWLGE
RnRad30      --------------LTQFTEAQLQSHFGEK-------------NGSWLYAMCRGIEH-EPVKPRQ--L-PKTIGCSKNFPGK--TALATREQVQWWLLQ
BtRad30      --------------LTQFSESQLQSHFGER-------------NGSWLYAMCRGIEH-DPVKPRP--I-TKTLGCGKNFPGK--TALSTRDQVQWWLLQ
DrRad30      --------------LTRFSRAQLEQHFGDK-------------TGPWLYDLCRGIEF-EPVKPRQ--L-PKSIGCSKNFAGK--TCLRTKQQVQYWLHQ
MmRad30(1)   --------------LTQFTESQLQSHFGEK-------------NGSWLYAMCRGIEH-DPVKPRQ--L-PKTIGCSKNFPGK--TALATREQVQWWLLQ
PtRad30      --------------LTQFTESQLQSHFGEK-------------NGSWLYAMCRGVEH-DPVRPRK--I-PKTVGCGKNFPGK--TALATREQVQWWLLQ
GgRad30(2)   --------------VTKFSEMELQTHFGDK-------------TGSWLYDLCRGIDD-EPVKNRH--L-PQSIGCSKNFPGK--TALATQKEVQHWLLQ
XlRad30      --------------LTQFTVQHLQNHFGDK-------------TGSWLYSLCRGIED-EPVKPRQ--L-PKSIGCSKNFPGK--TSLSTREQVQYWLLQ
SsRad30(2)   --------------LTRFSQAQLGQHFGDK-------------TGQWLFDLCRGIDL-EAVKPRQ--L-PKSIGCSKNFPGK--TSLTTREQVQHWLYQ
AoRad30      --------------LIQFSQAQLGQHFGDK-------------TGQWLYDLCRGIEF-EAVKPRQ--L-PKSIGCSKNFPGR--TSLATKEKVQYWLHQ
PnRad30      GAEETFKKLTAHTFLQHCGLAELTKHVGQE-------------TAAYVHRICQGNDDNEPVEEKK--VQLKMFSCVKQFDQRSGSALVRVEQLEYWVRL

ScRad30      FCAELTSRIQDLEQEYNKIV-IPRTVSISLK-TKSYEVYRK-SG-PVAYKGINFQSHELLKVGIKFVTD----------LD---------IK---G-K-
KlRad30      FCSELNYRVHDLEQEYEKVI-MPRTIVIMIK-GKAGMKYTQ-TR-RITTASSSITSRELFINATKLINE----------ID---------KQ-H-G-K-
CgRad30      FNGELTARIVDLEQDYNKIV-VPRTVSLNLR-SYTGDVRRK-SG-PLVINNSKYLSRDLLKTCVKLMQE----------IH---------DKFAAK-D-
CcRad30(1)  LAAELALRLNEARQ-ISPNLWPKNIVLHARK--GYE--SSR-SKQAVFPFTREVTVDTVAAAGDKLWKE----------LV---------GN-------
CnRad30      LAGELNVRLRQSRE-IMPGLWPKTLVLSYRQ--GIE--PTR-SRQIPFPFTRNLSTDYIMKYAKKLWDE----ATQPMLKG---------NM-------
ClRad30      LAAELALRLNDARD-VSPNLWPKTIVLHARK--GYE--SGR-SKQAPFPFTREVTVDVIASAGDKLWKE----------LV---------GN-------
CmRad30      LAAELALRLNDARN-MSPNLWPKTLVLHARK--GYE--SSR-SKQAPFPYTREVTVDIIAAAGDKLWKE----------LV---------GT-------
SsRad30(1)   LAAELALRLNDARV-SMPTLWPKSIVLHARK--GYE--TSR-SKQATFPFVRNVTVDVIATAADKLWKD----------LT---------DD-------
TtRad30      MAAELTLRLNDARK-ISPGLWPRTIALSTRQ--GWS--ATQ-RKQAPFPFLRNVTIDAVAAAGDKLWKE----------LV---------GP---L-NS
PpRad30(1)   LAAELALRLNDART-SSPSLWPKTIVLHARK--GYE--AGR-SKQSPFPFHREVTVDIIAAAGDKLWKE----------LV---------GK-------
HsRad30(1)   LAQELEERLTKDRN-DNDRV-ATQLVVSIRV-QGDK--RLS-SL-RRCCALTRYDAHKMSHDAFTVIKN----C------N---------TS---G-I-
GgRad30(1)   LALELESRLIKDRS-QNHRV-AKQLMVVIRM-QGDT--R----L-SRFCAVTRYDAQKIFNDAFALIQN----C------N---------MA---GAH-
CdRad30      LAQELLERLTKDRN-DNDRV-ATQLAVSIRV-QGDK--RLS-SL-RRCCALARYDAHKMSQDAFAVIRN----C------N---------TS---G-T-
AmRad30      LASELESRLGKDRS-QNGRI-AKQLMVSIRM-QGDL--KPH-GL-TRCCALARYDAHKISTDAFALIRN----C------N---------MA---GAQ-
HmRad30      LSGELVERIAVDRA-NHNRI-PSSLSVGVRAEQMNF--RST--------SLQPSILTSILPRIEESEEASAAEL------MIAKKIADIAFG---VVR-
JjRad30      LAQELEERLTKDQN-DNDRV-ATQLVVSIRV-QGDK--RLS-SQ-RRCCALTHYDAHRMSQDAFATIRN----Y------N---------TS---G-I-
EeRad30      LAQELEERLTKDRN-DNDRV-STQLVVSIRF-QGDK--RLS-SL-RRCCSLTRYDAHRMSHDAFAAIKN----C------N---------TS---G-I-
LhRad30      LALELQERLNKDKD-MNGRV-AKQLTVGVRQ-AGGQ------SF-SRCCALVRYDAVKITSDSLAIIKS----L------N---------TA---GNH-
EgRad30(1)   LAGEIVERVAIDRA-NHNRL-PTALSLFLRSDQSDT--RSR--------ALSPNILASILPRSQQMEEE-ETEA------QIARRIAEMAYG---SVR-
SvRad30      LSLELESRLNKDRS-QNHRV-ARQLMVVIRQ-QGDT--R----V-SRLCALSRYDAQKMCNDAFTLIQN----C------N---------VA---GAH-
PbRad30      LALELEERLNKDRD-QNNRV-AKQLSIGIHM-QGSK--HAS-GL-SRCCALSQYDAKKMSRDAFALIQN----C------N---------QA---AGQ-
PmRad30(1)   LALELESRLNKDRS-QNHRV-ARQLMVVIRQ-QGDT--R----L-SRLCALSRYDAQKMCNDAFALIQN----C------N---------VA---GAH-
DmRad30      LSSEINDRLEKDFI-ENNRR-AKHMVVQYVQ-DIDG--EEV-AS-SRSTALRDYDQESIVRLSLDLIKAN----------T---------KTFLRPGS-
RnRad30      LALELEERLTKDRT-DNGRV-ATQLVVSIRV-EGDK--RLS-SL-RRCCALTRYDAHKMSQDAFATIRN----C------N---------TS---G-V-
BtRad30      LAQELEERLIKDRN-ANDRV-ATQLVVSMRI-QGDR--RLS-SL-RRCCALTHYDAHKMSHDAFATIRN----C------N---------TS---G-T-
DrRad30      LALELEERLNKDRD-VNGRV-ARQLTVGVRQ-AGGQ--RSG-SF-SRCCALVRYDAMKMTNDSLTIIKS----L------N---------TA---GAH-
MmRad30(1)   LALELEERLTKDRN-DNDRV-ATQLVVSIRF-QGDR--RLS-SL-RRCCALPRYDAHKMSQDAFAAIRN----C------N---------TS---G-I-
PtRad30      LAQELEERLTKDRN-DNDRV-ATQLAVSIRV-QGDR--RLS-SL-RRCCALTHYDAHKMSRDAFAVIRN----C------N---------TS---G-I-
GgRad30(2)   LALELESRLIKDRS-QNHRV-AKQLMVVIRM-QGDT--R----L-SRFCAVTRYDAQKIFNDAFALIQN----C------N---------MA---GAH-
XlRad30      LSLELEGRLQKDRD-ANNRV-AKLLTVGLNQ-MGKR--LYG-SM-SRCCALTRYDAQKISSDAFVLLKS----F------N---------AA---GMH-
SsRad30(2)   LALELEERLTKDRE-LNGRV-AKQLTVGVRQ-LGDK--RPS-SF-SRCCSLARYDATKISSDSFAILKS----L------N---------TA---GNH-
AoRad30      LALELEERLTKDRD-ANGRV-AKLLTVGVRQ-LGDK--RPS-SF-SRCCALARYEATKISGDSFAIIKS----L------N---------TA---GNH-
PnRad30      LCEEVVVRCEDERI-ENKRF-PSQLTIQFIR-AKPG--EKSRT--YKLGIAQDTTVDELYTAAMNVMR-----------------------LHLDS---
```

```
ScRad30     NKS-YYPLTKLSMTITNFDIIDLQK--TVVDMFGN----------------------------------------------------------------
KlRad30     LAG-VYPLRELSLTLSNFDIIDKGK--TVLDMFGN----------------------------------------------------------------
CgRad30     PSK-FYPLINLNVIISNFDILDHQK--TVLDMFGN----------------------------------------------------------------
CcRad30(1)  --ATTMKISSVQLSFTGIDVSEPGQ-QSIEGFFK-----------------------------------------------------------------
CnRad30     --KLNV----IALSFTGLEKLEEGQ-QGIEGFFS-----------------------------------------------------------------
ClRad30     --SLTMNVSSVQLAFTGIDVAEPGQ-RTIEGFLK-----------------------------------------------------------------
CmRad30     --STQMNVTNVQLSFTGIDFAEAGQ-KTIEGFFK-----------------------------------------------------------------
SsRad30(1)  --AKTVNITSVQLAFTGLEKTEMGQ-KSIEGFFT-----------------------------------------------------------------
TtRad30     TKPLSTKITHVSLSFNGVEAGEVNQ-QGIEGFLS-----------------------------------------------------------------
PpRad30(1)  --NTNLKISSIQLAFTGIDHAEQGQ-QKIEGFLK-----------------------------------------------------------------
HsRad30(1)  QTEWSPPLTMLFLCATKFSASAPSSSTDITSFLSSDPSS-----L----------PKVPV-T-S--SE-------------------------------
GgRad30(1)  QAAWSPPLISVHLAASKFSAPTF-LSAGIASFLTSDTSS-----D---GT-------DAG-S-TEATS-------------------------------
CdRad30     QTEWSPPLTMLYLCATKFSASAPSPGTDITIFLSNDPSS-----L----------PKVPM-TTS--SE-------------------------------
AmRad30     QAAWSPPLTMLLMSASKFSEAPTLLPAGIATFLTSEAPT-----A---G----------------ATA-------------------------------
HmRad30     NIAGSNPINNITLAAGKFKSDHVFLCGNVKKLLSEQQAK-----Q---QI-------DE----Q-----------------------------------
JjRad30     QTEWSPPLTMLFLSATKFSASAHSACRDITVFLSSDASS-----V----------PKVPI-T-S--SE-------------------------------
EeRad30     QTEWSPPLTMLFLCATKFSASAPSSCTDITNFLSSDSNS-----L----------LKLPV-T-N--SE-------------------------------
LhRad30     QEAWSPALTCLHLSASKFSDVPSSSSGGIAGFLSSDAPS-----A---QS-------LLA-S-T--QT-------------------------------
EgRad30(1)  SLIGTAAITNISLSVGKFKSDHSAACGDVKKFLTEPPK-------------------------------------------------------------
SvRad30     QAAWSPPLISVQLSASKFSEPVT-LSTGITTFLTGDTQP-----D---GT-------ATT-S-QTTTS-------------------------------
PbRad30     QATWSPPVTVLQLSASKFSKVSTVLSVDITSFLTNHSQH-----T---QD--------------ITTT-------------------------------
PmRad30(1)  QAAWSPPLISVQLSASKFSEPVT-LSTGIATFLTGDTQP-----D---GT-------ATT-S-QNPTS-------------------------------
DmRad30     ESALNNAIKFLGISVGKFETVSSGQ-NKLQEMFANQAAKKRRVSGDEPGQ-------LPKVE-MEKKQKQTDEFKMKSFFANYLQGAKKEDAKADGISA
RnRad30     QTEWSPPLTMLFLCATKFSASAPPACTDITVFLSSDSGC-----Q----------PKVPA-A-S--SG-------------------------------
BtRad30     QTEWAPPLIMLFLCATKFCSSTPSPGPDITTFLSSDPSS-----L----------PKVPI-T-S--SE-------------------------------
DrRad30     QEAWSPALTLLHLSASKFSDAPSSSSAGITGFLSSDAAS-----S---PS-------QSS-S-S--QR-------------------------------
MmRad30(1)  QTEWSPPLTMLFLCATKFSAAAPPACTDITAFLSSDSSC-----Q----------PKVPI-A-S--SE-------------------------------
PtRad30     QTCWSPPLTMLFLCATKFSASAPSSCTDITTFLSSDPSS-----L----------PKVPA-T-S--SE-------------------------------
GgRad30(2)  QAAWSPPLISVHLAASKFSAPTF-LSAGIASFLTSDTSS-----D---GT-------DAG-S-TEATS-------------------------------
XlRad30     QAAWSPPLTLLQLSASKFSSTELPSS-GITSFLSKEASS-----S---QKPPSPTPRRSS-A-T--ET-------------------------------
SsRad30(2)  QAAWTPPLTLLHISASKFNDSPSAGAGGITSFLSSDVTS-----T---QA-------S-----------------------------------------
AoRad30     QAAWTPPLTLLHISASKFSDAPTAG--GIAGFLSGEVTS-----T---QT-------LFS-N-T--QT-------------------------------
PnRad30     ----MFPMSTLIMNAKNFHDLDSQAVTTISSFFTRTAEQA------------------TQR--------------MEEEIKQFAAK-------------

ScRad30     -----------------------------------------------QVHTFKSS-------------------------------A------------
KlRad30     -----------------------------------------------RAISKSSS--------------------------------------------
CgRad30     -----------------------------------------------QKQVFNTK-------------------------------T------------
CcRad30(1)  -----------------------------------------------SKPPSK-------------------------------------------RQ-
CnRad30     -----------------------------------------------NTKPKAAD-----------EST-IGPSSST-T--M-PRVISKLNNISK-RT-
ClRad30     -----------------------------------------------TGYTNK-------------------------------------------RV-
CmRad30     -----------------------------------------------PTSK---------------------------------------------RQ-
SsRad30(1)  -----------------------------------------------TRDVQQ-------------------------------------------TS-
TtRad30     -----------------------------------------------KAPSSP-------------------------------------------RK-
PpRad30(1)  -----------------------------------------------PSSTK--------------------------------------------RT-
HsRad30(1)  -----------AKTQ-----------GSGPAVTATKKATTSLESFFQKAAERQKV-K---------EAS-LSSLTAP-T--QAPMSNSP-----S-KP-
GgRad30(1)  ------STRPRVKFF-----------RS-PSKEHRQKPANAIESFFQKVAERQQS-Q---------AVP-SG-LPAA-T--H-AHSPAA-----S-SM-
CdRad30     -----------AKTQ-----------GSGPAVTPTKKAATSLESFFQKAAEKQKV-K---------EAS-LSSLTAT-T--QAPISSSP-----S-KP-
AmRad30     ------PISPNAKSL-----------GS-PRKEPGKKPVNAIDSYFQRAAEKQQA-C---------AAA-GPCVPVV-I--G-AGSLLP-----C-PD-
HmRad30     -----------EQTE-----------V--EAERLDKNKEVKRESFFRKFIEKSEE-P---------Q--------------------------------
JjRad30     -----------TKTK-----------GSSPAVPATKKATPSLQSFFQKAAEKQKI-K---------ETS-LASFSTA-T------SNSP-----S-KP-
EeRad30     -----------ASTQ-----------GSGPSVTTTKKATSSLESFFRKAAEKQKV-K---------EAS-LPSRTPT-T--QNPTNNSP-----S-KL-
LhRad30     ------PK Q-PSPK----------------------SPGAIRSLFEKAAEKKK--Q---------QVE-ESSKDEH-V--SSSLPQKA-----T-GI-
EgRad30(1)  --------------------------------------LVKRESFFRQFFEIGNQ-A---------EPS-------------------T-----PKK--
SvRad30     ------CGRARVKFF-----------RS-PSKEFRQKPASAIESFFQKAAERQLS-Q---------AAT-ATSLPTA-A--T-AESAVP-----S-SS-
PbRad30     ------TDGMNAKFL-----------GS-PKKETSKKAVNAIQMLFQRAAKRKQA-Q---------AIT-GSDMIVP-P--A-TQS-IS-----C-HI-
PmRad30(1)  ------CGRAGVKFF-----------RS-PSKELRQKPASAIESFFQK------------------AAA-AASLPST-A--T-AEPAVP-----S-SP-
DmRad30     NPLAAAAGAPNKNFVEEYKHKLHAAVRT-EGTVLTSTPAEFKESFFSQYLKQQKK--T--------GQQGSVTS-RE-DSLD--VQELA-----E-ELD
RnRad30     -----------TRTQ-----------EPGPAAPTSKEAATSLESFFQKAAKRQRM-R---------ETS-FVPPKVA-V--E----KSS-----S-KP-
BtRad30     -----------AKTQ-----------GSGPAVTTPKKATTSLESFFQKAAEKQKV-K---------EAS-LSSPTAT-T--QAAMSPS------S-KP-
DrRad30     ------SPMERPAQK-----------RKRQQDQRQRENTTSISAFFQRKSLEKQ--EIQNSLCPGDQST-SASGEHA-C--GDTAAQKT-----T-AI-
MmRad30(1)  -----------TRTQ-----------GSGPAVPTSKEAATSLASFFQKAAKKQRM-K---------ETS-FVPLNTA-T--E----KLS-----S-KP-
PtRad30     -----------AKTQ-----------ECGLAGTATKKVATSLESFFQKAAEKQKV-K---------EAS-VSSLTDT-T--QAPTSNSP-----S-KP-
GgRad30(2)  ------STRPRVKFF-----------RS-PSKEHRQKPANAIESFFQKVAERQQS-Q---------AVP-SG-LPAA-T--H-AHSPAA-----S-SM-
XlRad30     ------SSPKTPKSQ-----------RK----DTESRTPSSIQLLFQRAAEKREIRE---------EKV-PPTNMPG-S--PSGSPTEL-----N-TT-
SsRad30(2)  -------TQSCSGSK-----------K-----DSTPKRPGAIQSFFQRSAEKQR--K---------EGE-EEENDGG-Y--GTFLPPSS-----F-PV-
AoRad30     ------SIPPSSEPK-----------N-----ESPRKQPGTIQSFFKKAAEKQK--Q---------EDT-KDVDEGG-D--D--LDSTG-----F-LT-
PnRad30     --------R----------------DRD-RSPT-RKSNKQKISAFFGPSSSICNG--D--------DTE------ATII-------------------T

ScRad30     -------------------------------------------------------------------------------------GK----E--SSK--
KlRad30     -------------------------------------T-----------------------------------------------DN----N--VVKIE
CgRad30     -------------------------------------------------------------------------------------IS----P-KLDPVT
CcRad30(1)  -------------------------RED------------------------------------D-------------HGGT----ST-L-----P---
CnRad30     -------------------------RSPSSD--------------------------------SS----------------S----TIPTAVEVLP---
ClRad30     -------------------------RDGSNA--------------------------------ED-------------VNEQ----HS-G-----S---
CmRad30     -------------------------RDS---------------------------------------------------AEL----TD-F-----N---
SsRad30(1)  -------------------------LKR-----------------------------------------------------------------------
TtRad30     -------------------------RKRQAN--------------------------------SD-------------DEEIV---ET-L-----P---
PpRad30(1)  -------------------------RAASDA--------------------------------EN-------------SHQAMLDDHL-D-----T---
HsRad30(1)  -------------------------SLPFQ-----------------------------TSQSTG-------------T------EP----FFKQKSLL
GgRad30(1)  -------------------------EHPEGDS---------------------------A----G-------------L------AS------EQRELE
```

```
CdRad30       ------------------------SLPFQ------------------------------TSCTTG-------------T------EP----FFKQKSLL
AmRad30       ------------------------RHQDLED----------------------------P--ILN-------------T--------------VQ-SPE
HmRad30       ----------------------------------------------------------------K-------------R------ES----FFHRYI--
JjRad30       ------------------------LLIFQ------------------------------TSQTTG-------------T------EP----FFKQKSLL
EeRad30       ------------------------SAFFQ------------------------------KSQTKG-------------I------EP----FFKQKCLE
LhRad30       ------------------------SSFFQ-RKTLEKKLQLADSLSDDG---------------------------------------------------
EgRad30(1)    ---------------------------------------------------------------VA-------------F------EG----SLHQAT--
SvRad30       ------------------------EHQGRAG----------------------------IDLPVG-------------L------AS------MQCDLE
PbRad30       ------------------------SSQIPED--------------------------------------------------------------------
PmRad30(1)    ------------------------EHQERAA----------------------------VGLPVG-------------L------AS------VQCDLE
DmRad30       AIEADNSKDFEEDTEEETELTSDTHMSKPEG----------------------------QSSDAGQEQDPNTLNDSTGN------DL------YVETGI
RnRad30       ------------------------SLLFQ------------------------------TSGSPG-------------T------KS----FFKQKSLL
BtRad30       ------------------------SLPFQ------------------------------SSHTMG-------------T------EP----FFKQKSLL
DrRad30       ------------------------STFLQ-RESLEKQEIQNVSLDDEDQSVCAPEENTCREKPAG-------------I------ST----SLQMKSLE
MmRad30(1)    ------------------------SLVFQ------------------------------SSQTTG-------------S------QS----FFKQKSLL
PtRad30       ------------------------SLPFQ------------------------------TSHATG-------------T------EP----FFKQKSLL
GgRad30(2)    -----------------------EHPEGDS----------------------------A----G-------------L------AS------EQRELE
XlRad30       ------------------------SNMA-----------------------------------SG-------------C------QS----FLKQQTQE
SsRad30(2)    ------------------------SFSAV-ERSVSEKEKTTVSSTSCHSS---SVVSSSASPHTG-------------I------TS----FFQRKTLG
AoRad30       ------------------------SSSPH-KTPAADS--QLEKDTNCPAS-------TKTGSHSG-------------I------SS----FFHKKSLE
PnRad30       PSV-------SATAHSDSGI-------------------------------------------------------------------------------

ScRad30       ---------------------------------------A-----------------------------------------------------------
KlRad30       GEDI-----------------------------------HS--------------------DSKGGPYDAS---------------V--SE----TLEG
CgRad30       EVED-----------------------------------K-----------------------------------------------------------
CcRad30(1)    -------------------------------------------------------------G----------------------------------GDE
CnRad30       -------------------------------------------------------------SK--------KVRPM----------NQPLSKRKKGLDA
ClRad30       -------------------------------------------------------------A----------------------------------GAV
CmRad30       -------------------------------------------------------------T----------------------------------QAE
SsRad30(1)    ------------------------------------------------------------------------------------------------SRE
TtRad30       -------------------------------------------------------------A-----------------------------------SS
PpRad30(1)    -------------------------------------------------------------S----------------------------------GAS
HsRad30(1)    LKQ------------K-----------------------Q-----------------L-NNSSVSSPQQNPWSNCK----------ALPNS----LPTE
GgRad30(1)    ASV------------K-----------------------QS--------------------PRDGSPASPCKRPPC----------EELLS----DATQ
CdRad30       LKQ------------K-----------------------Q-----------------L-NNSSVSFSPQKQQSSPK----------ESPNY----LPTE
AmRad30       AIV------------K-----------------------QS--------------------PKGGNSPS-SKPLPC----------EES-S----GTAG
HmRad30       ---------------------------------------------------------------------------------------------------
JjRad30       LKQ------------K-----------------------Q-----------------L-NKSS-TYVPQNLQSNSE----------WLPNS----FPEG
EeRad30       ALK------------K-----------------------QA----------------L-HNPSVSCLTPN-----------------------------
LhRad30       ----------------------------------------------------------RSTLTEDQSDS------------------------------
EgRad30(1)    ---------------------------------------------------------------------------------------------------
SvRad30       SPV------------K-----------------------QT--------------------HSDASPTSPYKTLPQ----------EKLPS----DTTQ
PbRad30       ----------------------------------------S--------------------MEPSNPNLMQKQLLM----------EQSLQ----EATL
PmRad30(1)    SPV------------K-----------------------QS--------------------PSDASPRSPCKSLPR----------EKLPS----DATL
DmRad30       VPPTLTEDELKPSTSKRKFDEIESSVSNYKECYVEFAVPNLRTDIL-------------PTIKCDQCGANIPDEVKSLQTHRDHHFAQELS----RTLR
RnRad30       LQH------------T-----------------------E-----------------L-SNAAAPCPPQA---SSA----------VQPSR----LPTD
BtRad30       LKQ------------K-----------------------Q-----------------L-TNPSVFFPSQKPQSSPK----------ELPNC----FPTE
DrRad30       KHQ------------I-----QNSLCVG-----------DQ--SVRAP---------GENPPREQTSDESPAGISSFFQMKSLEKHRIQNS----PCTG
MmRad30(1)    LQH------------T-----------------------Q-----------------L-SNSAAPDPPQA---SPA----------AQPSC----LPAE
PtRad30       LKQ------------K-----------------------Q-----------------L-SNPSVVFPPQNPQSSPK----------KLTNS----FPTE
GgRad30(2)    ASV------------K-----------------------QS--------------------PRDGSPASPCKRPPC----------EELLS----DATQ
XlRad30       LQE------------A------YNF--------------C--------------------SRTDSEPNG------------------------------
SsRad30(2)    RNQ------------Q-----PVTAASH-----------SD--SLATGRTRCSTVGYDTIGSVQLLTDTVGCEIAGSYTAPGAETCDEQTA----NVKG
AoRad30       RSS------------Q------VSVSTI-----------PD--P-----------------ETEQMPESVKNEDSG-----------DTVA----CMSS
PnRad30       ---------------------------------------------------------------------------------------------------

ScRad30       ------------------------------------------------------------------------------------DEKTPKLECCKYQVQ
KlRad30       EPPSASSP----------------------------------------------------------------------------PTMAENLTCKQCGET
CgRad30       ------------------------------------------------------------------------------------DEDAKKFSCEKCKVH
CcRad30(1)    NEHS--------P-----------------------------------------------SSR---------------------GTPGTSFTCPRCNKT
CnRad30       FLIKKPSDVTSSSS------HSNISTP----SS-----ASVSDLEITPIEPPQ--SSRISSHT---------------------KTDMDSWTCPKCAQT
ClRad30       TGDT--------GT-------------------------------------QT--IREGEDQS---------------------MTGIPSYNCPRCSRT
CmRad30       VGQP--------ES-------------------------------------SA--GDAKEDSA---------------------TDIIVSHTCTRCKKT
SsRad30(1)    DDEP--------LV-------------------------------------SP--EKTLPTSS---------------------YVEAVSFKCTRCRKT
TtRad30       SGE---------------------------------------------------------SGP---------------------KPDIDYFICDRCKRR
PpRad30(1)    SLKR--------QP-------------------------------------DN--ETSNKNQP---------------------TGSAPCYTCNRCSKT
HsRad30(1)    YPGCVPVCEGVSKL--------------E-E-S-----S---KATPAEMDLAHNSQSM-HASSASKS---VLEVTQKATPNPSLLAAEDQVPCEKCGSL
GgRad30(1)    TPSTPPSSRILPKL------K-----P---------------ALEGNEQNLPP-PEQA-LLPHVSP---------------------GDQQCCEKCGQY
CdRad30       YPDCAPVCEKVLHL--------------E---S-----S---KGTPAEMDLAQNSLSR-LASLTSKS---ALEVAQKAATTPSLLAAEDQVPCEKCGSL
AmRad30       TSSAALVPRTTLKAEPEAASE-----Q---------------AAEWKEGSLAPSSGCG-QAPHTSP---------------------GDQLLCEKCGQQ
HmRad30       ----EPVQEGVPST--------------------------------S-----K-SPNY-FVQH---------------------DSTMDTILCEECGLQ
JjRad30       SSDGVPVCAIALKP--------------E---S-----S---KTSPEEMDLAQNSPNM--PAFSAPK---SLEVAQKATTTPSLTTTEDQVSCEKCGSL
EeRad30       -----PDYEDVLKP--------------E---S-----S---ETVCAEMGLAQSSPSR-LAPFTSKS---AVDVAPKAATTPSLLDAEDQVSCDKCSSL
LhRad30       A-----PCETASEA----------------------------VVS--------------LIAPSDVN-----------------TSSEDLHACERCGLK
EgRad30(1)    ----EPKAQEETKA--------------------------------S-----K-ELVY-FQRH---------------------NSTAEKVLCEECGSY
SvRad30       TPSTPPSSRTLLKL------Q-----P---------------AMEGNEQNVPPSPELT-LLPPASP---------------------GDWQPCEKCGQL
PbRad30       TGEGCVSS-SEIKM------F-----PDS-FKE---------KKVQPAPGLSQGGAMS-LAVPSSM---------------------DDCLLCEKCNQK
PmRad30(1)    TPSTPPSSRTLLKL------Q-----P---------------AMEGNEQNLPPSPELT-LLPPASP---------------------GDQQRCEKCGQL
DmRad30       STEREERTQSRQKI------S-----LKPTP------------PKKSKKTAGSGSSSY-STAPPSNSITKFFRAKPTQEQA---PSDPQMNQCPECKAF
RnRad30       RADSRASCEEVCKP--------------V---S-----S---KAVSTEVNVAGNS-S-----LAYN----SQEMTQR--------AAEDQVLCEKCDSL
BtRad30       NPDCAPDCEKMLKL--------------G---S-----P---KTTPTEMDLAQNSLSS-LASLTSTS---ALEVAQKATMTSSFLAAEDQVPCEKCGSL
```

```
DrRad30       DPNIRAPAENTSRD----------------------------QTSERSTSGMSGCFQQ-AVAPAGER---VCS-----------PAEEDLQVCERCGMK
MmRad30(1)    CVDS-GPDDGAVKP--------------V---S-----S---KAS-TEMNVAGDSPNV-LDSPAYN----SQEVTQR--------ATEDQVLCEKCDSL
PtRad30       YPDCTPVCQAVLKV--------------G---S-----S---KTTPAGMDLAQNSPSS-LASVISKS---APEVAQKSTTTPSPVAAEDQVPCEKCGSL
GgRad30(2)    TPSTPPSSRILPKL------K-----P---------------ALEGNEQNLPP-PEQA-LLPHVSP---------------------GDQQCCEKCGQY
XlRad30       --PVDGAAENASGP----------------------------VLTATGEHESCCTTSA-LQEASLAG-----------------HSEEDIMCCEKCGLK
SsRad30(2)    KHTKHTQLTSSNQA--------------E-DQSLGSGEG---TCTLSEEKQSEGSEMP-YQPPSDPP---QCHAI---------VAKEDLLTCECCGQD
AoRad30       LQSNHASEFTSHQP----------------------------LCEELKDEVDIDPELN-HLPPS--------------------VAREDLLKCERCGQE
PnRad30       ----------------------------------------------------------------EAT-----STCTE-------TASTSDHFCDEC-KR

ScRad30       FTD--------------------QKALQEHADYHLALKLSE-----------------------GLNGAEESSKNL---------------S------F
KlRad30       LQD--------------------KKLFQEHVDYHLSVQLSE-----------------------QINGVSENSTML---------------T------H
CgRad30       FDN--------------------LKEYDEHKDYHAALKLSE-----------------------SLNGVESTSKNL---------------S------I
CcRad30(1)    LSLNPEI---QALIDDGEREAALASLKLEHDDWHFARDLAQ-----------------------EDNRTAQAQTQ------------------------
CnRad30       FTVPDEL------DLGEEQVGLLRSMKQEHEDWHFAKSLQD-----------------------GGDGNGGTSAP------------------------
ClRad30       FRLPES-----TPEIAIVQEETLAAIRMEHDDFHFAQDLAR-----------------------SSQPNKVLTV-------------------------
CmRad30       LSLNLEL---SRFKDSEEQKTAIAALIMEHDDWHFAQDLVR-----------------------EEIPIRPIQP-------------------------
SsRad30(1)    IALREDQ---ATGG-PNEVAQRLATLRSEHGDFHFAQDLAH-----------------------STDG-------------------------------
TtRad30       IALPEELTMFASELDEDTKKDALGSLRAEHQDFHFAQDLSK-----------------------MPSDDERPEKP------------------------
PpRad30(1)    LHLPN------LALAEDEVQAHLAKLQMEHEDFHFAQDLSR-----------------------EGAATFKVAS-------------------------
HsRad30(1)    VP---------------------VWDMPEHMDYHFALELQKSFLQPHSSNPQVVS--------------------------------------------
GgRad30(1)    VL---------------------AWELPEHMDYHFAVELQR-----------------------SFQEPSSP---------------PALAEVPIAKAA
CdRad30       VP---------------------VWEMPEHTDYHFALELQE-----------------------TFLQSHSSTPQV-VP--------------------
AmRad30       VL---------------------VWDLPEHMDYHFAVELQN-----------------------SFTELSFPHTSPV---------RTSPCAMPTSPCA
HmRad30       VP---------------------VHVMPEHSDFHFARKLQA-----------------------EWTKEISQQAP-------------------PNR--
JjRad30       VP---------------------VWDMPEHIDYHFALELQK-----------------------SFLQPHVSKPPA-VS--------------------
EeRad30       VP---------------------VWEMPEHMDYHFALELQK-----------------------SFSHPQSPSPQV-VP--------------------
LhRad30       VL---------------------VWEMPEHMDYHFALDLQK-----------------------SFSSSSASGNGPV------------------TAGA
EgRad30(1)    VL---------------------VHLMPEHRDFHFARRVQQ-----------------------EWNREVNRTLQ-------------------P----
SvRad30       VL---------------------VWEFPEHMDYHFALELQS-----------------------SFLEPSPP---------------MAPEAAPNAKAV
PbRad30       VV---------------------VWEFTEHLDYHFAVELQN-----------------------SFSG-----------------------SSSFRSVD
PmRad30(1)    VL---------------------VWEFPEHMDYHFALELQS-----------------------SFLEPSPP---------------MAPEAAPNAKPV
DmRad30       IK---------------------CVDMPEHLDYHVARNLQR-----------------------ELNQQDLRTRT-------------------AALNK
RnRad30       VP---------------------VWDMPEHLDYHFALELQK-----------------------SFLQPCTSKPQA-VP--------------------
BtRad30       VP---------------------VWDMPEHSDYHFALELQN-----------------------SFLQPHSSNPQV-VP--------------------
DrRad30       VL---------------------VWEFPEHADYHFALDLQK-----------------------SFSSGSPS---------------------------
MmRad30(1)    VP---------------------VWDMPEHTDYHFALELQK-----------------------SFLQPCTSKPQA-IP--------------------
PtRad30       VP---------------------VWEMPEHTDYHFALELQKSFTQSHSSDSQVADYHFALELQKSFMQSHSSNSQV-VP--------------------
GgRad30(2)    VL---------------------AWELPEHMDYHFAVELQR-----------------------SFQEPSSP---------------PALAEVPIAKAA
XlRad30       LL---------------------VWEIPEHMDYHFAQELQD-----------------------SFSAPSPSRAPPV------------------CAPA
SsRad30(2)    VL---------------------AWEMPEHNDYHFALDLQN-----------------------SFSSASTTTNASLHSSSLSTASRP----PLRAGAL
AoRad30       VL---------------------VWEMPEHNDYHFALDLQN-----------------------SLSSPTGPANIPSSSSAVSSSSSSASLNAPRVGAA
PnRad30       VV---------------------SEPRAEHADFHFALKLNQ-----------------------TQRNE------------------------------

ScRad30       GEKRL-LFSRKRPNSQHTATPQKKQVT--SSKNILSFFTRK-K
SpRad30       GEKRL-LFSQKRSSSQHSTAPRKKQAT--SSKNILSFFTRE-K
KlRad30       AERIL-LFGKKAKKNAASVQKPKK-----KDGGIMKFFK---K
CgRad30       GEKRL-LFSKEKRVKKKPISKN--KVN--SSGNIYNFFNK--K
SbRad30       GEKRL-LFSRKRPNSQHTATPQKKQVT--SSKNILSFFTRK-K
KmRad30       AERLL-LFG-KRTKSRKGTRKKS------PEGGILKFFK---K
CcRad30(1)    -------FSSTSSTAKKSLQNKKRR-K--VAVGIEKFFDRK-S
CnRad30       -------G--QRRSNTTPSLGQKEK-R--NVEGIRAFFTPKPQ
ClRad30       -----------KTPEQKPRPTKKRR-K--DSEGIEKFFSR--K
CmRad30       ---------SASTSHKKTPKTKKRR-K--DPVGIERFFDR--K
SsRad30(1)    -----------SVPVPRGSSSKRKK-V--ESEGIAKFFIK--K
TtRad30       -------NPKWTVQPRKKGKHKDGG-T--NGEGIERFFTK--R
PpRad30(1)    -----------TAQKPAAKQTKKRK-V--EPTGIEKFFRK---
HsRad30(1)    --AVS-HQGKRNPKSPLACTNKRPRPE--GMQTLESFFKPLTH
GgRad30(1)    ASGSP-AKVRNKAKAPAGPNAKRPRES--VARTLDFFFKRLPP
CdRad30       --ASS-PQSKRNPKSPLASNNKRPKLE--GMKTLKSFFKPLTQ
AmRad30       MPLSP-ANGKSKPKTPTGSSMKRRRQG--VTRTLDYFFKRLPP
HmRad30       -SLSMNGKSDRSSKKKGRGGKSKSDI---RNAKIDAFFVKR-N
JjRad30       --ATS-PQGKRSHKSPLASSNKRPRPG--GMQTLESFFKPLTH
EeRad30       --VVS-PQGKRNPKSPLASSNKRPRPA--GMLTLESFFKPLTH
LhRad30       --PQP-SRGKSRTKIQSGPQAKRARVQ-GNSGTLDSFFKK--T
EgRad30       --LQVKKETRTIRETKRGRGRGKRDL---GLAKIDTFFLKR-D
SvRad30       ASRSP-AKAKNKLKTPAGSSAKRPKEG--VTKTLDFFFKPLPP
PbRad30       SLDSP-VKDKNKSREQRTTSAKRPKQD--ANRTIDFFFKPLPP
PmRad30       ASRSP-AKAKNKLKTPAGSSAKRPKEG--VTRTLDFFFKPL-P
DmRad30       EKISP-VQPKKQSQKKLNSTISASSSG---TKTIAQFFSQS-N
RnRad30       ---VS-PQGKRNPKSPLASSNKRLRPH--GMQTLESFFKPLTH
BtRad30       --ASS-PQSKRNPKSPLASNNKRPRPE--GMQTLESFFKPLTH
DrRad30       -----------QMKTPSGSQAKRARVQ-EPRGTLHSFFKK--T
MmRad30(1)    --AVS-PQGKRNPKSPSASSSKRLRPH--GMQTLESFFKPLTH
PtRad30       --ASS-PQGKRNPKSPSASSNKRPRPE--GMQTLESFFKPLTQ
GgRad30(2)    ASGSP-AKVRNKAKAPAGPNAKRPRES--VARTLDFFFKRLPP
XlRad30       -LTPL-ARSKNKAKKSLASNAKRPRTD--GNRTLDAFFKS--P
SsRad30(2)    ---QL-SRGKTRARGQSTPQPKRPRSQGGATGTLDSFFKR--S
AoRad30       GTAQS-SRGKTKTKGQSGPPTKRHRSQGGSTGTLDSFFKR--N
PnRad30       ------------AMASAGASRK--------KGPLDAFLR---R
```

Fig. S1B: <u>Category II DNA polymerase eta:</u> DNA polymerase eta from protists and plants containing only PCNA interaction motifs at the carboxyl terminal domain.

```
(I)
CLUSTAL W (1.83) multiple sequence alignment

TbRad30     -----------------------MRCIVHIDMDCFYAQVEAVRLGVDCRTEPYVLSQWG------NLIAVNYPARKF-G--IGRFDTVTDALE----KC
BsRad30     ---------------------MCAHVICHIDLDCFYAQVEALRLGIDFRNEPYVLVQWG------NLIAVNYPARKA-G--IGRFETIRDAIA----KC
LmRad30     MALLPT---------S-LPGADPMRCIAHMDMDCFYAQVEAVRLGVDCRVTPLVLSQWG------SLIAVNYPARAR-G--VRRFSNVSEAQA----LC
FsRad30     MLESSFFTETSMLSASHNNKQRQQRVILLLDLDCFYAQCECVRLGFDVTTTPLALLQFN------SALAVTYPARTLFSQPIERGDGWEAVRDKSGGKC
CeRad30     ----------------------MKRVISLIDMDCFYAQVEQ-RDNPSLWGQPVIVVQHSRQGIEGGILAVSYEARPF---GVKRGMTVAEAKL----KC
GsRad30     MASDEYTNF------A-HSDLEQFPVIIHLDLDCFYAQVESVRLGLD-PSTPLCVQQWD------GVIAVNYAAREY-G--ISRHERIEKVKE----KC
PpRad30(2)  -------------------MAENCRVIVHLDLDAFYAQVEAVRRDIP-ADVPLAVLQWE------SLIAVSYAAQRH-G--VRRFGSLEEAIR----AC

TbRad30     PHVKISHVATYAAG---EV---------------EY-R--YHEN--P--------------------S--KQTHKVALEPYREASRKIFRILDSF----
BsRad30     PEIKYSGVPTFEVG---ST---------------EY-Q--YHSN--P--------------------N--KGTHKVSLDPYRRASKKIFDVFGSF----
LmRad30     PGLIVALSPSYRMG---EA---------------VS-Q--YHPH--P--------------------V--QDSYKLSLEPYRHASRQIFSILAAT----
FsRad30     YAVHVPLLP---VQ---GDTTNATTTSLAETLQEEY-DSLYKLS--KEQQEEAR-----KRDLWVRRF--AAEGKASIECYRIASAKIFETVHEFLETE
CeRad30     PQISICHVP---IG---EY---------------V--------------------------------------DKADIQKYRDASAEVFRVLNNY----
GsRad30     PNCVLVHVETVGFP---DQ---------------SSSR-ENVSN--PDL---------------L--NHGKSETKVSLDRYREASAKIFQLLFSY----
PpRad30(2)  PDMLLVHVDTVEQAPSGGD---------------AN-E--HIHTRTYAPV---RAEHWHTQLRHKLSN—PGKMKVSLKMYRESSMKVMDFLQAQFH---

TbRad30     --------DGVEVEKGSVDEAFLDVTKAAHMKQGEMGLLSSQGELR-LEDVADPTTIVIPSRQAEIAAWLKEHGREFSD-VFDVTLHPQPT-----
BsRad30     --------PGVQVEKGGVDEAYLDVTEAAHSYVESFYQEHGHH-I--TQQDIDAYTRMCPNRDEDLHQFLQLQEAGGVV-PHIETFAPAPT-----
LmRad30     --------PGVQVEKGSVDEAYVDVTEAARRELAEVRAAAAGASLDPLEDVMEPSTRLIEDRRAEMEAWLSARGTSLAA-VFDEPMRALVRGECGA
FsRad30     KSQMDDTNVKIMLERASIDEFYLDVTDAVNKNDDYWNVRQD--------LLE----------------------ENSILQ-TTHLVRP--------
CeERad30    -------DSQIIIEKASVDEAFLDLSAYTNQKLQELRENEGL------EEFLQAAITYLPTTHLATGEDVKENEHLREDV-LLEYIEN--------
GsRad30     --------SE-LCEKASIDEAYLDVSEQVQDILVATRNQRSS------------IG--------SIFEQLSMTEKERAYY-FETIFQPFGKR----
PpRad30(2)  ------PSDRVVFERASIDEVFLDVTHLARTRLSALLASGKD KNS----TLL------------ELYANIHLSHLGGSH---VPRLADGQE----

TbRad30     -----------------AENMSLLAAASRVVWTIRQKIYDELRYDCSAGIAHNKLLAKSISARHKPNQQTLLFPDCVASVMWDLPFKSIRGFGGKFGEV
BsRad30     -----------------PEYHRLLLAGCAVVKTIRDKVRTELGYDCSAGVSHNRMLSKCISALFKPNQQTLLYPEGTLGFLFDFKFSKLRMFGGKLGQT
LmRad30     ELEGSRAFCVGADDAAYAERCLLLCAASRVVHRLRQRIYAELHYDCSAGIAHNRVLAKCISATHKPNQQTLLLPDRSASALFELPLSGLRGFGGKLGAA
FsRad30     ----------EGE-ESVPWNGDVFRRACWLAHQLRQYVRNTLSFTMSCGISVNKTLAKLSAGYGKPNGQALLLPEYVEYMLEQTQIRKCRNLGGKVGAA
CeERad30    ------------A-RNC-TENLLLLIAAVTVEQIRQQIHEETQFFCSAGVGNNKMMAKLVCARHKPRQQTLIPWFYVREILRLTPIGDVRGFGGKMGNR
GsRad30     ---------------EL-PLSQILSIGCAIAAKIRYAIYSQFNYTSSAGIAENKLLAKLGSSLNKPNRQTLISPKAVPFLLENLPLKKLRGLGGKLGTR
PpRad30(2)  -----------------SEQDKLLLVGAMISDELRRAVKRELRFAMSGGISSNKALAKLASPMNKPDAQTLVLHAHVPELMVRSKVKKIRNLGGKLGST

TbRad30     VRLACGG-K-ETCR-EAWLHSLCAMSKFFE-S-----V-------------------------------------GDAEYAYRRLRGYDE-GKIRERSI
BsRad30     IEQGLHG---TTCG-DLWQYSEEAIAAVVG-D-----K-------------------------------------ETGAYIYRRVRGFDA-DTVAARTM
LmRad30     VSAVCGG-V-TECR-EAWLVPLAQLRKLDG-TCDVGDGEDAEGDREGRKRRPIARKRGRAASASLERDLQGLVAHTTSLYVFYRLRGLAE-DTILNRPL
FsRad30     VQSLLPQDVPTTVASIAKYLSMPQLVEGFHGD-----E-------------------------------------ATARWVYDVARGIDP-QPVVAKNT
CeRad30     IQEMLN---ITLMG-ELLEVDISQLIETFP-N-----Q---------------------------------------HEYLRSVAEGHCD-EPVRPRKE
GsRad30     IEERTNA---KTAK-EAQNVTLEKWNEIVG-R-------------------------------------------ENAEWIYNLVRGIDY-SPVNARGI
PpRad30(2)  LTA--AG-F-ETAA-DAQALSAPELAEILG-S----DM-------------------------------------STARVVYNLVRGIDPSECMPKDEK

TbRad30     S---KSLMASKAF-S-PP----S-STANGVQKWVTVLSGELSARYEDFCNTYGVKGHSFNVKLGNRGLDQPSSV---------AN--KTFPL--P-E-L
BsRad30     A---KSLLAQKVF-S-PL----T-DDVSVLRKWFVVLAQELVERLQEFGDMFGVCGRNMNVKLGGSGLQDSSDV---------GN--KSFPL--P-T-P
LmRad30     S---KTIIASKNF-G-RI----T-TSVEMVRRWIIVLTSELCSRYEEFTALYQIRGRSFNIKLGNDGFRSTGGL---------SS--HTVAL--P-E-A
FsRad30     GALTKTVTTFKSF-T-A-------CPVGDTTSWIELLARDIVTRIEKDAHRNNRYPRVCTIQYHPTVVIHANADRFDKRKLLYQN--KSFRTSFPPQ-R
CeERad30    S---SSIAVSKNF-PGKL----SIRSVLELKKWLDGLTKELAKRLATDQAENKRTAENLVYSLLTEDGKPQKT---------LKI--TSYH---PDT-L
GsRad30     T---KSILAAKSF------K-AE-CSWEGMEKWIKILAYELCERLRKDETMNSRRPINFIVHYSSVG----SVS---------SSKSIPFPN--G-K--
PpRad30(2)  P---KSMLAAKNLPG-AA----R-ERVEDVAVWLHVLVAEMAERVAGDAQRNNRMPKTLKVSFRSNR-----VT---------TT--RQVSW--P-GDA

TbRad30     VTPQTLVSAAMQCVTAIMANR--PG-VVVNAVMLTIGSFKKQESDESG-----------------------VRSQQTTLRDFFKLKNPGKRERNHT---
BsRad30     TTAEALTGVAVRYASIVINNA--PGRVQINSVSMSISDFRKMQQNEDVLAA---------------------- NQQTLDRFFKKRPRDDSATSKK---
LmRad30     MQPDILAAVAMREVQAVFRNK--PG-AAADSVTLTIGGFVSDGTGATGSVAPLAATPGNRTSAGQRLAARGHLRQQTLLASFLAASAADGGAAGAMHQR
FsRad30     LERDAKVRHLVHDIQSKLLQHCKESTSNIDRLGIAASDFITI---------------------------------ASCLDLFVQRGV------------
CeERad30    FEQIWAAMKGLNKTNATKNEDSGPWTPPILNISLSATRFQPGIPAQ----------------------------NRSIHEWLYEKKTRKSRRKAAIYDE
GsRad30     DRVSSLSRNAMKILKTAN--N--FC-FPCNRLSLSVTKFLKTSSD------------------------------LGSIAAFLKP---Q----ENH---
PpRad30(2)  LSHDALFQTAVNILRDSS--Q--LA-LPCGFIGLTACNFVDRARG------------------------------VQKMELFLSDTATEKKTQPAGCAG

TbRad30     ---------------------------------------DEAM--VIT--LS-S---C-----------------------------------------
BsRad30     ---------------------------------------EYDT--TPR--TTET---P-----------------------------------------
LmRad30     SGGNVAEVL-SSDGSGLDDS-----DSNGSVH-----IEDDAV--ILS--LS-S---HSMSSNSGDMSTVVLIDRTPPLPERDVVPETRCVAKVRALDV
FsRad30     -------------------------------------------------------S-GQD--GENLSKKRKHVDLNL---DCTVIGET-----------
CeERad30    NDGREDVIF-VEPPEKLKKP-----NPTPKST-----GTDG-D--FIV--LG-SDSDDDVVQPKTDDNWIFVDGKK-----------F-----------
GsRad30     -QKLVEELVQESSSIKSSETTAEET--------------------------------------------------------------------------
PpRad30(2)  IAKERDTDTEEV-DSQNRTAAPLVGAARLSRRRDSEI---RSRSVCSVPLSEERYPTNDESVGVCDKPEAGHETGAAA----VIAGAVH---VEGQEGE

TbRad30     --------------------PSSPVSRES--LKSTPGTR-V---------------V-------FDVEDDGDDEER-----------------------
BsRad30     ----------------E----KPSRRGEKENEQDEDGDD-V---------------V-------IIEPALS-----------------EGLKTPI--PP
LmRad30     CKSSPCEAAGAASPPQTPHLCSPLPAGKAATEESQGESR-D---------------A-------TEYHEEHQRHLGGHHHIVPQP-PRSALALPFLTAP
FsRad30     ----------------------ITPKQKDED---A--DR-ILAEKLQADFDRENRLLQAS-------ERLQ----------------------------
CeERad30    ----------------------SKMAWRHLP---PD-IKKHYQ----------HRIALEEARALKSKTGGSRKRAGAH--------------------T
GsRad30     ----------ASNLQ-Q----PPEKCKVVEVEQREEEDY-ILAQRMQVEEWLKLR-S-------EGD--YNCDDR-----------------------R
PpRad30(2)  RHDMGMTAEQEALLRQEVEWKDDTDATLARKLYAEEERRMRIV-----------RTQ-RKLL--R-KRSRG-E—G-D---GKSRPPP-----VISGTKT

TbRad30     ---------------------ES--GEKDV--HII----D
```

```
BsRad30      ATAGRK--------------KQP--RPHDAEVIEL--D
LmRad30      PSSPAPANTVEVVEDNG---GVE--GDDGVTII----D
FsRad30      -TLGK---------------KKNKTKRIESFFVKK--K
CeERad30     DN-AQ---------------KPKKPKSLESFFKKKK-P
GsRad30      ES-RK---------------KKRRVQTLDHFFH----K
PpRad30(2)   KSHSAGCQSGDVWEQLARRGIKR-----SAKEM--ETM
```

II)

```
AtRad30      ----------------------------------------MPVARPEASDARVIAHVDMDCFYVQVEQRKQPELRGLPSAVVQYNEWQGGGLIAVSYEA
BdRad30      ----------------------------------------MPVARPEPQEPRVIAHLDLDCFYVQVEQRRNPALRGQPTAVVQYNDWKGGGLIAVSYEA
HsRad30(2)   ----------------------------------------MPVAKPESSDSRVIAHVDMDCFYVQVEQRKKPQLRGLPTAVVQYNEWKGGALIAVSYEA
SlRad30      ----------------------------------------MPVARPELSDCRVIAHVDMDCFYVQVEQRKQPSLRGQPTAVVQYNSWQGGGLIAVSYEA
CaRad30(1)   ----------------------------------------MPVSRPELSDSRVIAHVDMDCFYVQVEQRKQQSLRGQPTAVVQYNSWQGGGLIAVSYEA
CsRad30(1)   ----------------------------------------MPVARPESSDSRVIAHVDMDCFYVQVEQRKQPHLRGLPTAVVQYNEWQGGALIAVSYEA
OsRad30      ----------------------------------------MPVARPEPQEPRVIAHVDMDCFYVQVEQRRNPELRGQPTAVVQYNDWKGGGLIAVSYEA
ZjRad30      ----------------------------------------MPVAKPESSDSRIIAHIDMDCFYVQVEQRKHPELRGLPTAVVQYNEWKGGSLIAVGYEA
CsRad30(2)   ----------------------------------------MPVARPESSDSRVIAHVDMDCFYVQVEQRKQPHLRGLPTAVVQYNEWQGGALIAVSYEA
AhRad30      MRLSKMGAMADDSRRGGNWELGTEIQLAARADRGSSEKRAMPVAKRETSDGRVIAHVDMDCFYVQVEQRKQPNLRGLPTAVIQYNSFKGGALIAVSYEA
BnRad30      ----------------------------------------MPVARPETSDARVIAHVDMDCFYVQVEQRKQPELRGLPTAVVQYNEWQGGALIAVSYEA
RsRad30      ----------------------------------------MPVARPETSDARVIAHVDMDCFYVQVEQRKQPELRGLPTAVVQYNEWQGGGLIAVSYEA
CcRad30(2)   ----------------------------------------MPVAKRETCDGRVIAHVDMDCFYVQVEQRKQPNLRGLPTAVIQYNSYKGGALIAVSYEA
PmRad30(2)   M--------------------------------------EEFLSSYCPMIAICGLVSMPSLLYFIVEQRKQPDLRGLPTAVVQYNAWKGGALIAVSYEA
EgRad30      MLWKELL------------EGKQRSERKKEEEEEEEEGEEMPVAKPVSSDGRVIAHIDMDCFYVQVEQRKHPELRGLPTAVVQYNSWKGGALIAVGYEA
AoRad30      ----------------------------------------MPIARPEPLDARVIAHVDMDCFYVQVEQRRQPELRGLPTAVVQYNSWKGGGLIAVSYEA

AtRad30      RKCGVKRSMRGDEAKAACPQIQLVQVPVARGKADLNLYRSAGSEVVSILAKSGKCERASIDEVYLDLTDAAESMLADAPPESLELIDEEVLKSHILGMN
BdRad30      RKFGVKRSMRGDEAKHVCPGINLVQVPVAREKADLNIYRSAGSEVVAILSTKGKCERASIDEVYLDLTDAAKERLLESPPESSEIIFEEATKSNILDL-
HsRad30(2)   RKFGVKRSMRGAEAKEVCPEIQLVQVPVNRGKADLNGYRNAGSEVVSILSRSGRCERASIDEVYLDLTDAAETMLAENPPQSLETIDEEALKSHILGLN
SlRad30      RKFGVKRSMRGDEAKQVCPEIHLVQVPVARGKADLNAYRNAGSEVVSILSRRGRCERASIDEVYLDLTIAAEAMLADNPSECLETISEEVVQSHVLGLE
CaRad30(1)   RKFGVKRSMRGDEAKKVCPEIHLVQVPVARGKADLNSYRNAGSEVVTILSRRGRCERASIDEVYLDLTIAAEAMLVDNPPECLETISEEVVKSHVLGLE
CsRad30(1)   RTYGVKRSMRGHEAKQVCPEIELVQVPVARGKADLNTYRNAGSEVVSILARTGRCERASIDEVYLDLTDAAEKMLAEAPPEHFELVDEETLKSHILGLT
OsRad30      RKFGVKRSMRGDEAKMVCPSINLVQVPVARDKADLNVYRSAGSEVVTILSTKGKCERASIDEVYLDLTDAAKEMLLESPPELLELIFEEATKSNILGL-
ZjRad30      RKYGVKRSMRGDEAKRVCQEIQLVRVPVARGKADLNTYRNAGSEVVSILARKGRCERASIDEVYLDLTEAAETLLAQAPPENMEVVDEEALKSHVLGLD
CsRad30(2)   RTYGVKRSMRGHEAKQVCPEIELVQVPVARGKADLNTYRNAGSEVVSILARTGRCERASIDEVYLDLTDAAEKMLAEAPPEHFELVDEETLKSHILGLT
AhRad30      RNFGVKRSMRGDEAKDVCPQIHLVQVPVARGKADLNSYRNAGSEVVSILARTGRCERASIDEVYLDLTDAAEKMLAEAPPEHFELVDEETLKSHILGLE
BnRad30      RSCGVKRSMRGEEAKAACPEIQLVQVPVARGKADLNTYRSAGSEVVSILSKKGRCERASIDEVYLDLTDAAETMLLEMPPEAREDIEEEVLKSHVLGLS
RsRad30      RRCGVKRSMRGEEAKAACPDIQLVQVPVARGKADLNTYRTAGSEVVSILAQSGKCERASIDEVYLDLTDAAESMLADAPPESLESIDEEALKSHILGMN
CcRad30(2)   RRCGVKRSMRGDEAKEACPQIQLVQVPVARGKADLNSYRSAGSEVVSILAQSGKCERASIDEVYLDLTDAAESMLADAPPESLESIDEEALKSHILGIE
PmRad30(2)   RKCGVKRSMRGAESKQVCPQIQLVQVPVARGKADLSTYRNAGSEVVSILSRKGRCERASIDEVYLDLTDAAETMLAEAPPEILEVIDEEVLKSHVLGLN
EgRad30      RALGIKRSMRGEEAKAVCPQIHLVQVPVARGKADLNSYREAGSEVVSILARMGRCERASIDEVYLDLTDAAETMLSETPPESLEIIDDEALKSHILGLK
AoRad30      RKFGVKRSMRGDEAKEVCPDINLVQVPVARGKADLNLYRNAGSEVVSILSSKGRCERASIDEVYLDLTEAAESMLLETPPEVLEEIDEEVLKSHVLGVT

AtRad30      REDGDDFKESVRNWICREDADRRDKLLSCGIIIVAELRKQVLKETEFTCSAGIAHNKMLAKLASGMNKPAQQTVVPYAAVQELLSSLPIKKMKQLGGKL
BdRad30      SSDVSNREENVRAWLCRADADYQDKLLACGAIIVAQLRIKVLEETQFTCSAGIAHNKMLAKLVSGMHKPAQQTVVPSSSVQDFLASLPVKKMKQLGGKL
HsRad30(2)   YEDGSDVKENVRKWINRHNTDHRDKLLACGILIVAELRMQVFKETEFTCSAGIAHNKMLAKLASGMNKPAQQTVVPSASVNGLLNTLPIRKMKHLGGKL
SlRad30      -EVGSDARENVRHWFTRSDASRRDKLLACGAFIVAELRLQVLEETEFTCSAGIAHNKMLAKLASGMNKPAQQTVVPFSSVSKLLRTLPIKKMKQLGGKL
CaRad30(1)   -EDGSNAREKVGHWLTRSDASRRDKLLACGAFIVAELRLQVLEETEFTCSAGIAHNKMLAKLASGMNKPAQQTVVPFSSICKLLGMLPIKKMKQLGGKL
CsRad30(1)   NEREIDDKENVREWLCRKDADRRDKLLACGAIIVAELRMNVLKETEFTCSAGIAHNKILAKLASGMNKPAQQTVVSLSSVKGLLDSLPIKKMKQLGGKL
OsRad30      PSDVSNREDSVRAWLCRADADYQDKLLSCGAIIVAQLRVKVLEETQFTCSAGIAHNKMLAKLVSGMHKPAQQTVVPSSAVQDFLVSLPIKKMKQLGGKL
ZjRad30      DESGGDTKGNVRKWLCSSDSDRRDKLLACGALIVAELRMQVLKETEFTCSAGIAHNKMLAKLASGMNKPAQQTVVPLSSVEGVLDSLPIKKMKQLGGKL
CsRad30(2)   NEREIDDKENVREWLCRKDADRRDKLLACGAIIVAELRMNVLKETEFTCSAGIAHNKILAKLASGMNKPAQQTVVSLSSVKGLLDSLPIKKMKQLGGKL
AhRad30      IKEGSDAKEEVRKWIRRGDATYQDKLLACGAFIVANLRMQVLKDTEFTCSAGIAHNKMLAKLASGMNKPAQQTVVPQSSVGGLLESLPIKKMKQLGGKL
BnRad30      REDGDDFKESVRDWICRKDADRRDKLLGCGIIIVAELRKQVLKETEFSCSAGIAHNKMLAKLASGMNKPAQQTVVPYAAVQELLSSLPIKKMKQLGGKL
RsRad30      REDGDDFKESVRDWICRKDADRRDKLLGCGIIIVAELRKQVLKETEFSCSAGVAHNKMLAKLASGMNKPAQQTVVPYAAVQELLTSLPIKKMKQLGGKL
CcRad30(2)   IQDGSDAKDEVRKWLCRSDASYQDKLLACGALIVNDLRMQVLKETEFTCSAGIAHNKMLAKLASAMNKPAQQTIVPHSSVEGLLASLPIKKMKHLGGKL
PmRad30(2)   SEDGSDAKESVRKWLCRNDADRRDKLLACGAFIIAELRLQVLKETEFTCSAGIAHNKILAKLASGMNKPAQQTVVPFSSVKGLLGSLPIKKMKQLGGKL
EgRad30      HEDGSDMKENVKGWLRRSDADHREKLLACGVLIVADLRMQVLQETQFTCSAGISHNKMLSKLASAMNKPAQQTVVPCSSVQGLLESLPIKKMKQLGGKL
AoRad30      ADE-DDRATKVREWFYQSDADHRDKLLACGAIIVAHLRMKVLEETKFTCSAGIAHNKMLAKLASAMNKPAQQTVVPSSSVKDLLATLPVKKMKQLGGKL

AtRad30      GTSLQTDLGVDTVGDLLQFSETKLQEHYGVNTGTWLWNIARGISGEEVQGRLLPKSHGSGKTFPGPRALKSLSTVQHWLNQLSEELSERLGSDLEQNKR
BdRad30      GSSLQDDLGVKTVGDLLTFTEEKLQDYYGVNTGTWLWKTARGISGEEVEDRILPKSHGCGKTFPGSKALKNNASVKSWLDKLCEELSERIQSDLNSNKR
HsRad30(2)   GISLQNDMGVNTVGDLLQFPEEKLQERYGINTGTWLWNIARGISGEEVEGRLLPKSHGSGKTFPGPRALKTVPAVQHWLNQLCEELSERICSDLDQNKR
SlRad30      GTSLQIDLGVNTVGDMLQFSEEKLQGYYGVNTGSWLWNTARGINGEEVKGRLLPNSHGSGKTFPGPRALKTVASVGKWLNELCDELSERLQSDLEQNKR
CaRAD3O(1)   GTSLQIDLGVKTVGDLLQFSEEKLQEYYGVNTGSWLWNTARGISGEEVKERLLPNSHGSGKTFPGPRALKTVPSVEKWLSELCEELSERLQFDLEQNKR
CsRad30(1)   GSSLQSDLGVKTVGDLLQFPEEKLQELYGVNTGTWLWNIARGVSGEEVQGRLLPKSHGSGKSFPGPRALKTFASVEHWLNELCEELNERICSDLNQNRR
OsRad30      GSSLQDDLGVNTVGDLLSFTEDKLQEYYGVNTGTWLWKIARGISGEEVEDRLLPKSHGCGKTFPGPKALKNNASVKTWLDRLCEELSERIQSDLNQNKR
ZjRad30      GSSLQTDLGVFTVGDLLQFSEEKLQERYGFNTGTWLWNIARGINGEEVQGRLLTKSHGSGKTFPGPRALKTIASVQHWLNELCSELSERLYSDLDQNKR
CsRad30(2)   GSSLQSDLGVKTVGDLLQFPEEKLQELYGVNTGTWLWNIARGVSGEEVQGRLLPKSHGSGKSFPGPRALKTFASVEHWLNELCEELNERICSDLNQNRR
AhRad30      GSSLQSDLGCKTVGDLLQFSEEKLQQCYGINTGTWLWNIARGINGEEVEGRLLPKSHGSGKTFPGPQALKTLASVQHWLNELCEELSERLQSDLNQNKR
BnRad30      GTSLQTDLGIDTVGDLLQFSETKLQEHYGINTGTWLWNIARGISGEEVQGRLLPKSHGSGKTFPGPRALRSLSNVQHWLNQLSEELYERLSSDLEQNKR
RsRad30      GTSLQTDLGIVTVGDLLQFSETKLQEHYGINTGTWLWNIARGISGEEVQGRLLPKSHGSGKTFPGPRALRSLSNVQHWLNQLSEELYERLSSDLEQNKR
CcRad30(2)   GSSVQIDLGVNTVGDLLQFSEEKLQQLYGINTGTWLWNIARGISGEEVEGRLLPKSHGSGKTFPGPQALKTIDSVQRWLNELCEELNERLHSDLDLNKR
PmRad30(2)   GDSLQSDLGVNTVGDLLQFSEEKLQERYGVNTGTWLWNIARGISGDEVEGRLLPKSHGSGKTFPGPQALKTIASVQRWLHELCEELSERLQLDLDQNKR
EgRad30      GSSLQSDLEVNFVGDLLQFSEQKLQECYGINTGTWLWNIARGISGEEVEGRLLTKSHGSGKTFPGPRALKTLHSVQHWLNQLCEELSERLQTDLDQNRR
AoRad30      GSSLQSDLGVNTVGDLLHFSEEKLQELYGINTGTWLWTIARGISGEEVEGRVLPKSHACGKTFRGPQALKTTASVEHWLNQLSEELSERIQSDFDHHKR

AtRad30      IASTLTLHASAFR------SKDSDSHKKFPSKSCPMRYGVTKIQEDAFNLFQAALREYMGSFGIKPQGNKLETWRITGLSVSASKIVDIPSGTSSIMRY
BdRad30      VAQTLTLHARACK------ENECNLMKKFPSKSCPLRYGTGKIQEDAMKLFESALHDFWG--------SRNTGWSITSLSVTASKIFDDTSGTNSILRY
HsRad30(2)   MAHTLTLHARAYK------SSDSDSQKKFPSKSCPLRYGTAKIQEDAFNLFQAGLREYVGFYGVKTQGNHHSGWGITSLSVSASKIVPIPSGTCSIAKY
SlRad30      IAHTLTLHAHAYKVGFSCQSNDGDSFRKFPSKSCPLRYGTSKIKEDALSLFQAGLREYLGLFNVKISGNQNNGWGITGLSVTASKIVAIPSGTRSILNY
```

```
CaRad30(1)  IAHTLTLHAHAYKS------NDIDSFKKFPSKSCPLRYGTSKIKEDALILFQAGLREYLGLYNFKISGNQNSGWGITGLSVSASKIIAIPSGTRSIMNY
CsRad30(1)  IARTLTLHATAYK------SSDSDALKNFPSKSCPLRYGAAKMQDDAMNLFQAGLREYLGCYSTRVLGTQNNGWRITGLSVSASKIVEIPSGTSSIMKY
OsRad30     IAQTLTLYARACK------KNKSDSIKKFPSKSCPLRYGTVKIQEDAMKLFESGLHDFLG--------SQNTKWSITSLSVSASKIFDIPIGTSSILRY
ZjRad30     IARTLTLYATAYK------SSDSDSQKNFPSKSCPLRYGCAKIQEDALNLFQAALREYSGPQRAKTQGSRNHGWGITGLSISASKIVSIPAGTCSIMKY
CsRad30(2)  IARTLTLHATAYK------SSDSDALKNFPSKSCPLRYGAAKMQDDAMNLFQAGLREYLGCYSTRVLGTQNNGWRITGLSVSASKIVEIPSGTSSIMKY
AhRad30     IAHTLTLHARAYK------KGESDSLRKFPSKSCPLRYGTTKIQEDALTLFQAALREFLGFCNSKTHGNENNNWGVTSLSVSASKIVPIPSGTHSIVKY
BnRad30     IASTLTLHASAFR------SKDSDSHKKFPSKSCPLRYGVAKIQEDAFNLFQAALREYMGPFGPKPQGNKKETWRITGLSVSASKIVDIPSGTSSIMRY
RsRad30     IASTLTLHATAFR------SKDSDSHKKFPSKSCPLRYGVTKVQEDAFNLFQAALREYMGPFGTKPQGNKKETWRITGLSVSASKIVDIPSGTSSIMRY
CcRad30(2)  IAQTLTLHARAYK------TGDSDSHRKFPSKSCPLRYGTRKIQEDAIILFQAGLRDFLGFC-SKAHGSENNYWGVTSLSVSASKIVSIPSGTHSIVKY
PmRad30(2)  IAHTLVLHATAYK------VSDSDSHKKFPSKSCPLRYGTAKIQEDALSLFQAALREYLGSYTAKIQGSQNNHWGITSLSVSASKIVPIPSGTASITKY
EgRad30     IAHTLTLHARAYM------SSDSDSHKKFPSKSCPLRYGTTKMQEDAMNLFQAGLREYLGPFEVKMQGNKKIGWGITGLSVSAGKISDIPSGTSSIMRY
AoRad30     VAHTLTLHASAYK------ANDMESQRKFPSKSCPLRYGIVKIQEDAKKLFDSALKDFL--------GSQNSRWGITSLSIAASKILDTPSGTCSILKF

AtRad30     FQSQPTVPSRS---ADGCVQGNVAMTASAS-----EGCSEQRSTETQAAMP-EVDTGVTYTLPNFENQDKDIDLV-SEKDV---------------V--
BdRad30     ISP-SSSASSAVPGSSSTPELTPFLD---K------KLPMTPIHEEHCEPS-SKNEDCGN-----------SN--------------------------
HsRad30(2)  FHGQSTSHFSSMQPLDNFNAEATVSLPPGS-----ECYPEVNSPKAEVDFP-KEESWIADTVPDLDLQEQKDL-------P---------------C--
SlRad30     FHNQEEAFPQVKLSSEQLIQDAPLLSPSEEDSLESGGHLTLQTIEPWIACR-EENEETKYSMSLDR-QEEDKNTCKEKKIQVMEVGILTCRYHARLCYI
CaRad30(1)  FHNQQETCPQVKLSSEKLIQDAPLLSPSE-NCIENGGHMTLQSIEPWISCS-EEDEETKHSLPLDR-QL-DKEMCKEK---------------------
CsRad30(1)  FHGESPSSMLSKQSHDGFVEDADQPSHSGT-----ESSSEINLIQQQINFP-GEDTITKGKMPCLDQQDHKKDIQ-KEQIP---------------CHL
OsRad30     IKGPNSTVSPANLDCSSLPE-DPSLG---N------KLYIAPNHEEHCEPSLSEKEDYGNN----------SN--------------------------
ZjRad30     FHGQDPPGSSSKQSQNDHIEEAAPLSPSGS-----GSYSEMNSAEKLIEFT-GEDVKLEYEMPYLDQQEHRKDFS-EDQNS---------------CCL
CsRad30(2)  FHGESPSSMLSKQSHDGFVEDADQPSHSGT-----ESSSEINLIQQQINFP-GEDTITKGKMPCLDQQDHKKDIQ-KEQIP---------------CHL
AhRad30     FGGQVPSSSSFNQPLGNAFDEAVPSSPSGS-----ENCSGLISYELQQNYP-EGDTEIKNSEACLHEQDPLCN--------------------------
BnRad30     FQSQTTIPSCS---ASGFPQEHVAVTPSAS-----ESCSEQKSAETLPAMP-EEDITVTYTSPDLDNSYRDIDIV-LEKFP---------------CQD
RsRad30     FQSQSTIPSCS---TSGCLQEHVAVTPSAS-----ESCSEQKSAETEAAMPPDKDMSITDTSPDLDNSYGNMDMV-PEKFT---------------CQD
CcRad30(2)  FGGQFPSSSTSNQSQDVVIDGAAPS---GS-----ENCSGLVPYESQLEYP-E-DTGMKHSKSSLYQQDPLCN--------------------------
PmRad30(2)  FHG-HPSCSSTKQSQNNLVEEGTPVPPSGN-----ESYSEVNVTKPQIEFP-GEETMIKYAETSSDQLEDKIDLL-NDQNP---------------CCS
EgRad30     FQGHDRLCSPSKLAKEKFELEDPCSF-SEN-----ESNSTLDI-IEHTKLP-EEEATVNNESSDLESMEHKTSTN-SLEVP---------------FYS
AoRad30     FPGKSVSSTPLNGSDVFVQDEFSPI------------ALGIKSYTTESNDD-E--QQIRFD----------CE--------------------------

AtRad30     -S-------------------------CPSNEATDVSTQSESNKGTQTKKIGRKMNNSKEKNR-GMPSIVDIFKNYNATPP-SKQETQEDS-TVSSASK
BdRad30     -LAKQCCGI------------------------------------------EEKGVPNKSSKVKGTGSILKFLSQSQSALPEKRKIDGLTCGHQGQESS
HsRad30(2)  SLSKMQDGFTQDISPSLLSG---CLKQNQIKQQRDITKDEGRFRS-----EYKGRKGKRLKDK-GTASILELFKSYNPNPF-DSSLSQEFKKTAEGSDT
SlRad30     QLPDKETSICSSPDAEVYRGWE----KDQNESSRDYLTVK-----IRDSLESEDGKRKSNKEK-GMSTISRYFQSQLSGSL-LKAEHANTSRLSESSSL
CaRad30(1)  -LPDKETSICSSPDAEVYRGWE----KDQTESNGDYLTEK-----IRDSLESEEGKRKLNKEK-GMSTISRYFQSQNSGDL-LKVEHASTSKLSESPSL
CsRad30(1)  PLERGQDDVTQEACL-SSVGTESCSGINQSEENKVFHGDEPSGQCGVSSLNRPEQKRKALKEKPGTHSILRFFKKIDQSCPTVK-EHDDDLQNVELAST
OsRad30     -LAKQCQIK------------------------------------------EEKKVSKKLTEVKGTCSILKFLSQSP-VLSEKRKIDSLICSHPGPESS
ZjRad30     STKQARDDLTQGTFP--SSGTECFSEVNQSQRQRVIPVKGSGISSAVSSPKKLKQKRNKLKDK-GGYSILRFFKNNDPSCSSQKREHVENIE-VKAASS
CsRad30(2)  PLERGQDDVTQEACL-SVGTESCSGINQSEENKVFHGDEPSGQCGVSSLNRPEQKRKALKEKP-GTHSILRFFKKIDQSCPTVKEHDDDLQNVELASTG
AhRad30     -LSRKVDSSLKESSLESPAGSEHR--TTKSEPYRDLPAKDHRPLSSTPSLKAVEKKKTAGNNLKGNYSIMDFFNNYQNSQSSLEHKNVTNAHDVKTASS
BnRad30     AS-------------------------CQSNEAKEFPTQS----GTQTKTIGRKINNSKEKNR-GMPSIVDIFKNYNASPQ-AKEETQEDS-TVSLTSN
RsRad30     VS-------------------------CQSNEATEFPTQSGSNKGTQTKTSGQKI---KEKSR-GMHSVVDIFKNYSAAPR-SKQETQEHS-TVSLTSN
CcRad30(2)  -LSSKADSLTEESLLMSPLGSEDR--MIHDESRRDLPAKDHRLVSNISGLKAVGKKKRAGKQLQGNSSITNFFSNYHNSQSSSEQKNVTNAEGVKISSS
PmRad30(2)  SKNQARDEFTQETVPVSTSGSEHCSGMNQNQPMKNYSGEEPYVKLSMPSLNRQEQKRKAVKDK-GTCSILRFFKNQDPSCAPQKLEHVENIEDVKAPPL
EgRad30     SMSSLKKADFSQETPLSFSGRESHLEPKLTELQSKVSSKESTFEPGRPA-SEQQVKRNALRDK-GTPSLLRFFRRVDPSC---SIQSQVLAQSSDDTNI
AoRad30     -VTSKTNCTIN----------QD----------RTLPTSD-R--------KAKDK---------GSCSILRYFQG-TNSHSTDQAYNAFTQDHAPPSS-

AtRad30     RAKLS--------------SS-SH-NSQVNQ--------------------EVEESRE-TDWGYKTDEIDQSVFDELPVEIQRELRSFLRTNKQ--FNT
BdRad30     SGANKAEQH-------GRSAEAVD-RSNINT--------------------AGVHSAG-NKWMFNVEDIDPAVVGELPLEIQREIQGWTRPSKQ—AS--
HsRad30(2)  RLTIGFEPKGHSLAVGKEKPMNSS-GSSYSR--------------------DEGDGRR-EAWSYKIDEIDQSVVDELPPEIQDEIKAWVHPHKWRPN--
SlRad30     SDRQSELPQ--------ENSPARG-ESSVDTHL---C--------------SQIKLKR-PSWSYDIDKIDQDVLNELPKQIQEEVQAWLRPQK---PN-
CaRad30(1)  SDHQSEPPQ--------ENSPARG-ESSVDARL---C--------------SHIKLKR-PAWSYDIDEIDQDVLNELPKQIQEEVQAWLRPQKR--PH-
CsRad30(1)  GVGLTERP---------LEVSDTN-NGQCTL--------------------T-QFERR-EAWSYKIDEIDPSVVDELPPEIQDEVRSWLRPLKR--S--
OsRad30     SEPNKAEEH--------KAAQYVD-RNKFNT--------------------AGSNSASSSTWMFNVEDIDPAVVEELPPEIQREIHGWIRPPKQ--SS-
ZjRad30     -AGS--------------DCLKLN-EAELPKEN---P--------------V-EEQRR-EAWSYNVDEIDPSVIDELPPEIQDEVRALLRPQKR--PNN
CsRad30(2)  VGLTERP----------LEVSDTN-NGQCTL--------------------T-QFERR-EAWSYKIDEIDPSVVDELPPEIQDEVRSWLRPLKR--S--
AhRad30     FGLPSTMDS---------YSTCNQ-IEQPAERC---REEI-DS-NVGECSVPNMSQRR-QAWDYNIDEIDHSVIEELPPEIQQEFQAWLRPHKR--PNV
BnRad30     RGNLS--------------SSTNH-SSEVNQ--------------------EVEDRRD-TEWGYKVGEIDQSVFDELPYEIQRELRSFLRPNKR--PNS
RsRad30     RGSLS--------------SSTSH-SSEMNK--------------------EVGDRRE-TEWGYKVGEIDQSVFDELPYEIQREFRSFLRPNKR--PNA
CcRad30(2)  HGSQSADDS--------YSTPNR-VGQPAEHP---HEEI-DA-NKSGCSVGNIPQGRR-QAWSYNIDEIDPSIIDELPPEIQQEFRTWLRPHKR--SNV
PmRad30(2)  SPGIQSRS---------NNCLDHN-RSELPKER---PSED-AGNSNVCGSFQIEQRRG-GAWSYNIDEIDPSVIDELPPEIQQEVRAWIRPHKR--H--
EgRad30     RSSL---E----------KNNSIH-GDSLSE--------------------DVQ--RR-EMWRYNVDEIDPSVIDELPSEIQEEVRAWLRPQKRQ-A--
AoRad30     -GSESCSML---------NQ VHK-GGTLAEG-----KNV-HQ-----------NFQRSNTWALNVEDIDPSVIDELPLEIQKEVRGWLRPSKR--SNT

AtRad30     GKSKG-DGS--TSSIAHYFPPLN---------------------R
BdRad30     ------TKPR-GHTISSYFPPARS---------------------
HsRad30(2)  -----NTVER-GCKISHYFSPTKK--------------------S
SlRad30     ------TVKK-DLGITRYFLPAKD--------------------K
CaRAD3O(1)  ------TVKR-DSGIARYFLPAKN--------------------K
CsRad30(1)  -----NIAKQ-GSSIAHYFLPTRK--------------------T
OsRad30     ------SKTR-GSTISSYFQPAKSGQGLMEKELRSSKGGRPHNSG
ZjRad30     -----NIVKR-GSSICHYFLPARN--------------------A
CsRad30(2)  -----NIAKQ-GSSIAHYFLPTRK--------------------T
AhRad30     -----PNSKR-GSSITHYFLPDKS--------------------R
BnRad30     GKSKSGDGSA-SSSIAHYFQPLK---------------------R
RsRad30     GKSKG-DGSA-SSGIAHYFPPLK---------------------R
CcRad30(2)  ------VK-R-GSSITHYFLPDK---------------------S
PmRad30(2)  -----NTVKR-GSSIAHYFLPTKN--------------------T
EgRad30     -----NSARR-SVGIADYFSRNKK--------------------L
AoRad30     ---------R-GSTIAHYFSSSSKN-------------------S
```

Fig. S1 C: Category III DNA polymerase eta:

```
CaRad30(2)  --MSVKQETRP----------------------------------------L-------------------------------TIPLVTNPE-VATTLS
CaRad30(3)  --MVVNYL-DFNE----ADTH-----------------------------LK-------------------------------LENEDTGTLPEKK-NS
NcRad30     --MSSPPH-MQ----------------------------------------S-------------------------------FRDLSPAGAPGMK-RS
DhRad30(1)  --MSVKRDEFE----------------------------------------N-------------------------------KS-LTTFPNHAS----
UmRad30     --MSSPTK-TA-CKFDSLAPTGVEAFASSSGASSDMSAKFETTTHKEHLLPNLPTEEVHSSLTDRAPSLKAPEPTQQSVPSHTNLETAVHSSGPPP-WP
YpRad30     -------------------------------------------------------------------------------------------------MS
BmRad30     ---------MS----------------------------------------L-------------------------------PQP---------T-TS
StRad30     --MSVKQEFSP----------------------------------------G-------------------------------AC-LATTAAYSTS---
AnRad30     MPLSP-EPPGS----------------------------------------P-------------------------------PNSSND---SLKR-HS
GcRad30     -----------------------------------------------------------------------------------------------M-PS
OoRad30     ---------MD----------------------------------------D-------------------------------YTDWPSPTAAQRP-KS
FoRad30     --MSSSPL-NR----------------------------------------P-------------------------------LGASSPLLEGGRR-RS
PgRad30     ---------MS----------------------------------------E-------------------------------PTESSD---HLK--VS
CsRad30(3)  --M------------------------------------------------S-------------------------------DDSITFPHDPTSA-ES
NgRad30     --M------ES----------------------------------------D-------------------------------PAVSYDEDSHSRS-SS
McRad30     --M------AS----------------------------------------D-------------------------------PASPYDE--YCRP-SS
MmRad30(2)  --MS--SP-QY----------------------------------------T-------------------------------SSPPASNPLEGRR-RS
MrRad30     --MSTERS-G---------------------------------------------------------------EKHRFRYHSDKIEADYENSEHVP-CP
DhRad30(2)  --MSSSPP-D-------------------------------------------------------------------------FRGSSPAGAPGRA-RS
BgRad30     --MSSPSS-SG----------------------------------------F-------------------------------NSP--PASGTRHQ-KS
AfRad30     --MSSSQP-FL----------------------------------------A-------------------------------SSPISPATAGRRR-KS
SpRad30     --M------------------------------------------------------------------------------------------ELG-KS

CaRad30(2)  KF---------------------TFQNLD-D-LND-P--KKAYLSPLATIALIDLNAFFAQVEQIRLNLTN-QDPVVCAQWNSVIAVSYASRKFGITRM
CaRad30(3)  KLGINDYFTTNNITIQSKEYVDILLEDIIDEFVNQ-Y--K--PNMVPRIIALIDMDAFFTQVQHKICDIPK-DKPLVVLQRNTVIAVNYPGQARGIKRG
NcRad30     RF---------------------TYRHIQ-Q-LAA-S--S--TSCPLRVIAHLDLDCFYAQVEMVRLGIPD-DKPVAVQQWQGLIAVNYPARSFGIGRH
DhRad30(1)  TF---------------------TYKDLK-L-LEN-P--IVAYQSPLAVVAHVDLNAFFAQVEQVRLNLTA-DDPVVCAQWQSLIAVSYAARKFGIGRM
UmRad30     TI---------------------TYKHLL-S-AGT-L--N--PTNPLRVVAHCDVDAAYAQFEASRLGLDATVVPIAVQQWQGLIAVNYPARDAGVSRF
YpRad30     RF---------------------TYRNLY-N-LKT-P--EKSFDSPMACIVHLDMDAFFAQVEQVRLGLPP-GTPVACRQWDGLIAVGYAARASGITRH
BmRad30     RF---------------------THRHLT-L-LRA-S--S--PRTPLRIIAHIDLDAFYAQCEMVRLNTPR-TQPLAVQQWESLIAVNYAARAFNITRM
StRad30     QF---------------------TFKDLR-D-LND-P--SLSYLSPLSVISLIDLNAFFAQVEQLRLGLPV-ETPVVCVQWSTLIAVSYAARKFGIGRL
AnRad30     RY---------------------TYRHFQ-L-LRQ-A--S--PASPLRVIAHIDLDAFYAQCEMVRLGTPR-ERPLAVQQWDSLIAINYPARKFGITRM
GcRad30     EF---------------------TQTHLA-A-LKD-KHQS--HLSPLAVIAHVDLDAFYAQCEQVRLNLSK-TDPVVCRQWNGLIAVSYAARQYGVTRN
OoRad30     KF---------------------SYKDLL-Q-MAK-Y--N--VNSPLRVIAHIDLDAFYAQCEMIRLGVPE-DQPLAVQQWDALIAVNYPARSFGITRM
FoRad30     QF---------------------TYRQFS-Q-LAS-S--N--TSNPLRVIAHIDLDAFYAQCETVRLGIPE-DKPLAVQQWQGLIAVNYPAREFGIGRH
PgRad30     NF---------------------TYRQLQ-G-LRQ-G--S--TATPLRVIAHIDLDAFYAQCEMVRLGTPR-DVPLAVRQWDSLIAINYAARPFGISRL
CsRad30(3)  HF---------------------NQHDLQ-L-LAQ-S--S--PESPLRVVALVDYDSFYAQYESVRLGLDP-SKPLAVRQWNAIIALNYPAKDRGLKRT
NgRad30     RF---------------------TYRHFQ-L-LRS-F--S--TRTPLRVIAHIDLDAFYAQCEMIRLGTPR-DQPLAVQQWQSLIAVNYAARPFGVSRM
McRad30     QF---------------------TYRHLQ-L-LRS-F--S--TRTPLRVIAHIDLDAFYAQCEMVRLNTPR-DQPLAVQQWQSLIAVNYPARPFGVSRM
MmRad30(2)  KF---------------------TFKHLN-Q-LST-Y--S--TTTPLRTIAHVDLDAFYAQCEMVRLGVAR-DQPLAVQQWGALIAINYPSRKFGLTRH
MrRad30     RV---------------------TYRHLL-S-T-S-Y--E--PENPLRVIAHCDVDAAYAQFEASRLGIDSRSNPLVVLQWKQIIAVNYVARKFGVSRF
DhRad30(2)  QF---------------------SYRHLN-S-LAS-Y--S--TSSPLRVIAHVDLDAFYAQCEMRRLEIPD-DQPLAVQQWQGLIAVNYPARDRGVGRF
BgRad30     RY---------------------TYRHLG-Q-LAL-F--T--PNCPLRVIAHVDLDAFYAQCEMVRLNVPE-NQPLAVQQWQGLIAINYPARKFGLNRH
AfRad30     RF---------------------TYKHLA-Q-LAL-S--S--TTCPLRVIAHIDLDAFYAQCEGVRLGIPE-EQPLAVQQWQGLIAINYPARKFGLNRH
SpRad30     KF---------------------SWKDLQ-Y-CDKAG--T--QNSPLRVVAHIDQDAFYAQVESVRLGLDH-SVPLAVQQWQGLIAVNYAARAANISRH

CaRad30(2)  -DTIASCKSKCPNVIIAHAAVYK-K-GE-SHWAYVEG-LPAINKHKVSLDPYRRESRKILRVIGKSFD-------------------------------
CaRad30(3)  -MKLQEALSIYPDLAIPKTTYYD-DKIK-DRNSYN--------IKKISLEIYREASREIFDNIANYIPK------------------------------
NcRad30     -CN-SEAKKLCPDLIHQHVATWR-E-GD-AKWDYHPDAAANMATHKVSLDPYRLESRKILAIIKEELP-------------------------------
DhRad30(1)  -DTIS-ARQKCPNIIIAHAAVFK-K-GD-SYWSYIRG-LPDRSVHKVSLDPYRRESRKIMNIFREYCD-------------------------------
UmRad30     -ESIPEALKKCPDLHLVHVATYA-H-GS-NKPDYHPD--PKPETHKVSLDHYRRESTKILTIFKRTCPH------------------------------
YpRad30     -MRAPEAQKLCPNLVMAHAASFK---GE-DMWEYHAN--PSADDYKISLDPYRQAGKKVLNIVKQYSS-------------------------------
BmRad30     -LTAAEARKRCPQLVTQHVATFR-E-GEGAQWKYRDDAAECVATDKVSLEPYRVESRRILEGIRAALAAWAEGVMRDVGGGGGGGEGRGGGGTKNELQL
StRad30     -DTLQSAKQKCPNLVCAHAAVFK-K-GE-SHWAYTKG-LPNQATHKVSLDPYRRESRKIVKIFQQHCD-------------------------------
AnRad30     -ISAKEAKAKCADIVLQHVATFR-E-GEGGKWAYREDAWKNIGTDKVCLDPYRAESRKILGTIKRELSKCYSELNGDLSGLGLSS-------------Q
GcRad30     -EPAMAARAKCPDIVFAHVATFL-K-GD-TAWAYHEN--PRKATHKVSLDPYRRESRKIFGIFKEHCR-------------------------------
OoRad30     -RNVADAKRKCPTLMAVHVATWRVQ-NDTAVMGYHDMTNASISTDKVSLDHYRRESKKILAIFREGCP-------------------------------
FoRad30     -CNVEEAKKLCPELIVQHVATWR-E-GD-DKWAYREDAAAHIATDKVSLDHYRLQSRKILACIKEALP-------------------------------
PgRad30     -ITVAEARKLCPDLVLQHVATFR-E-GEGGRWAYRDDSFRNISTDKVSLDPYRAQSRKILQTMKEALAAWSADN----TEVESQN-------------T
CsRad30(3)  -ISVEEARRLCPDIVLQHVPTWR-E-GDD-CWRYRADVLDNLTTDK-ALDPYRDMSRRTMKLVRRILPA------------------------------
NgRad30     -ITAEEARKLCPQLLTPHVATFR-E-GEGENWAYRDGD-YSVQKDKVSLDPYRAESRKILATMKATLLTWAEGVYEGCRDQFSEP--------------
McRad30     -ITAEEAKKLCPQLLTPHVATFR-E-GEGENWAYREGG-YSVQKDKVSLDPYRAESRKILAVMKATLLSCAEGIYEGCRGQFSEP--------------
MmRad30(2)  -ITAAEAKKLCPNIVCQHVATWR-E-GD-DTWAYHDDAFKNMGTHKVSLDPYRRESKKILATIKESLPP------------------------------
MrRad30     NCTLEEAKQRCPDLRLVHVASYG-P-GD-KSPKYYED--PDPSSHKISLDMYRRESKKIMDIFQRQLCHDHVPYGHANYELESITTEGWSPSVLHMKGQ
DhRad30(2)  -TDIAQAKELCDNLICQHVATWR-E-GQ-EHWAYRDD--HDVSTDKVSLDPYRLQSRKILALIKESLP-------------------------------
BgRad30     -ITITEAKKLCPDLITQHVATWK-E-GE-ESWAYHDEAFKNMATHKVSLDPYRLESRRIVACIKESLP-------------------------------
AfRad30     -VTT-EARKLCPDLIMQHVATWK-E-GD-EKWAYHRDAFAHIATHKVSLDPYRLESRRILACIKDVLPA------------------------------
SpRad30     -ETVTEAKKKCPELCTAHVKTWK-A-GE-SEAKYHE--NPNPNYYKTCLDPYRHESVKILNIIKKHAP-------------------------------

CaRad30(2)  ------LTEKASVDECYIDLGREIYKRLIDLFPQLS------RGSRDN-P---EN-------SYANLPLI-PPA---LPLNLKWEG--EI-I-N-----
CaRad30(3)  -----CKIQISSIDEAYIDLSGNI-KLILEYIVLVL------KGE-RSDY---KGIEDLLCLTDEEILNY-KLNIVKNHKEMNF---MKC-LMD-----
NcRad30     --PHLQKVEKASIDEVFMDLSAQVHTILLERFPELA------NPP-P--Y---DD-------PTEMLPMP-SIT------ALDWQA--DA-L-------
DhRad30(1)  ------LVEKASVDECFLDFGRMIYYTLLQLFPDLG----------DE-V---EN-------INSNLPSI-PST---LPDSLYWVG--EI-I-K-----
UmRad30     -----GAVEKASIDESYFDLTIEVRKLLIQRFPHLA------HPP-P--D---DPVT-GAKGMDQPLPPP-PRLG-----LKQWKQ-LGY-VVPKTGDV
YpRad30     ------VVEKASVDESYLDLGPRIFNEIMIAFPQLQ------L----L-D---ND-------LDNFLPAP-PRAHELKIRGYNWDG--LGVL-E-----
BmRad30     QLQQLVRVEKAGIDEVFVDLSALVYETLLDRHPMLR------EGE-V--G---RD-------MSERLPRP-PTT------VLEWGK-EDM-L-------
StRad30     ------LVEKASVDESYLDLGRLVYKKLLSLFPLLA----------EG-Q---D---------SDYMPSL-PDT---LPEELQFRG--VV-I-K-----
```

```
AnRad30       IKLQEALVEKASVDEVFIDLSPLVFGVLLQRYPEMR------EKP-H--G---DD-------RVALLPCP-PTT------ALEWDT-EDC-L-------
GcRad30       ------VVEKASIDETYLDLGEVVYKRLLELFPELR------EDK--------LK-------PGAPLPEP-PTLAQLWEKGVEWMG--TI-Y-------
OoRad30       ------KVEKASVDESFLDLSGLVFGKLLERFEEELGVVDGEIVP-P--Y---GD-------TTERLRMP-EVA------GVEWGA--SH-L-------
FoRad30       --LDLQKVEKASIDEVFLDLSSQTHLILLERFPELS------NPP-P--Y---DD-------PTENLPLP-SIA------ALDWQT--DA-L-------
PgRad30       GQEASAVLEKASIDEVFIDLSPLIYRALLHRYPELR------MGT-Q--D---EN-------RDTELPIP-PTT------ALQWDT--DC-L-------
CsRad30(3)    --TPAPIVERAGVDEFYVDLSAQVYQILTERFPALG--------T-L--S---HN-------PEKTLPLP-PMDT-----SMHWKS--NK-V-------
NgRad30       --SDMVRLEKAGIDEVFVDLSALVFGTLLQRYEVLR------LAG-T--F---EGSK---DSPNRLLPRP-ETT------ALLWGE-DDE-L-------
McRad30       --SDMVRLERAGIDEVFVDLSALVFGTLLQRYEVLR------SAE-A--V---ECST---DSLGGFLPRP-EAT------ALVWGE-DDE-L-------
MmRad30(2)    --PPLQRVEKAGIDEVFIDMSAQIHSILLERYPELA------RLP-P--Y---DD-------LTENLPWP-SQS------TINWEA--DA-L-------
MrRad30       SKDHDIIFEKASIDESFFDLSRYVRKQILSRFPSLD------IRK-E--VNGFDADT-RAARLDAELPPI-PMHVRDEMSMRAWLA-LGT-WLP-----
DhRad30(2)    --QDLQKVEKASIDEVFLDLSAQVHAILLDRFPDLR------IPP----Y---DD-------PTERLPMP-PVT------ALDWKA--DA-L-------
BgRad30       --EEFQRIERASIDEVFLDLSALVHIKLLEQYPKLR------EPP-P--Y---DD-------PTEFLPHP-PRN------ILDWAA--DA-L-------
AfRad30       --PPLQRVEKASVDEVFLDLSAQIHSILLERYPEIS------GPA-P--Y---DD-------PTEFLTRP-PTS------ALDWDT--DA-L-------
SpRad30       ------VVKKASIDECFIELTSDVKRIVLEEYPYLKI------PS--------ED-------SNVALPQA-P--------VLLWPAEFGM-VIE-----

CaRad30(2)    -----------------------------------TEK-------------------------------------------------------------
CaRad30(3)    -----------------------------------LFPELFNEKE--------------------------------------------------RSKI
NcRad30       --V---------------------------D----LPD-------------------------------------------------------------
DhRad30(1)    -----------------------------------SED-------------------------------------------------------------
UmRad30       GKAMGIGRPEKAVAKRALLVDAPIGAGIVSELLPELPSANT--SQAADH----------------------SAQA-AQGQSTGKQPPPSWGSDQDHLTD
YpRad30       -----------------------------------IVT-------------------------------------------------------------
CmRad30       -----------------------------------LFPELFNEKE--------------------------------------------------RSRI
StRad30       -----------------------------------STE-------------------------------------------------------------
AnRad30       --V---------------------------D----LDE-------------------------------------------------------------
GcRad30       --G--------------------------------SYADES--ETRQIGDAVGTELYDFMNNPKKEIKAPVSGDQAPENTNT-TNPGFE-INNEKGLQS
OoRad30       --V---------------------------D----LDD-------------------------------------------------------------
FoRad30       --I---------------------------D----LDE-------------------------------------------------------------
PgRad30       --V---------------------------D----LDQ-------------------------------------------------------------
CsRad30(3)    --M---------------------------D----LPN-------------------------------------------------------------
NgRad30       --I---------------------------D----LDT-------------------------------------------------------------
McRad30       --I---------------------------D----LDT-------------------------------------------------------------
MmRad30(3)    --V---------------------------D----LEA-------------------------------------------------------------
MrRad30       -----------------------------------PSEH----------------------------------------------------REEQSLL-
DhRad30(2)    --I---------------------------D----LDD-------------------------------------------------------------
BgRad30       --V---------------------------D----LDS-------------------------------------------------------------
AfRad30       --V---------------------------D----LDL-------------------------------------------------------------
SpRad30       -----------------------------------EEV-------------------------------------------------------------

CaRad30(2)    ----------------E--KSE----------DNDIVSPPVIE-DWDDICFIIGSQILLEVRKDIFEELGYTTSAGLARTKQVAKLAAGFKKPDAQTII
CaRad30(3)    F--LILFNSYNEKGKFS--LFD------I-----------YGL-KFDEILILLGATIIYRVRNRLLQDLDYTCSAGISINKMLAKLVCSLRKPNGQSVL
NcRad30       ----------------E--NA------EL-----------EDP-DWDDVAILIGSEIVRKVRIAIKDKLGYTCSAGVACNKLLSKLGSAYRKPNQQTVL
DhRad30(1)    ----------------E--VPRNSDND-----QDLKANDPRIR-DWDDVCMLIGSQLLYEVRQQVFEELGYTTSGGLGKNKIIAKIAGGFLKPDNQTII
UmRad30       PSSSQQDDLDSEAALWD--HI------EY-----------GET-TWTDVALALGAELMNRVRQNVLDELGYTTSAGIGSNKTLSKLCSSWRKPNGQTIM
YpRad30       ----------------D--VSRFD--P-----DIRVHDDTMVT-DWDDLVLMFGARISKRMREQVYEELKYTCSAGIARCRSVAKLASAQNKPNNQTVV
CmRad30       F--LILFNSYNEKGKFS--LFD------I-----------YGL-KFDEILILLGATIIYRVRNRLLQDLNYTCSAGISINKMLAKLVCSLRKPNGQSVL
StRad30       ----------------E--ENLIQNNTIMSLSQQFPEGPPTIG-DWDDVCMLIGSMIVLDIRKALYDEMGYTTSAGIASNKQVAKLAGGFKKPDNQCVI
AnRad30       ----------------N--ET------EV-----------DDP-DWDDVAMLIGAEIVRSVRTAVWNSLSYTCSAGIAKNKMMAKLGSATNKPNKQTVV
GcRad30       P--TQLTDLPRETILAQ--NP------AL-----------EIQ-DWDDVCLLLGSVHIRNIRKHVLSSLQYTCSAGVARNRVLAKLGSSRHKPAKQVII
OoRad30       ----------------G--EE------GE-----------EEPIDWDDIIMGIAAEIVADIRGEVRRRLGYTCSAGISRNKMLAKLGSGYKKPNQQTIV
FoRad30       -------------------EQ------ET-----------VDP-DWDDVAILIGSEIVRKVRAEVRQKLGYTCSAGVASNKLLSKLGSAYKKPNKQTVV
PgRad30       ----------------Q--ET------EE-----------DDP-DWDDVVMLIGSEIVRSVRNAVWEKLSYTCSAGLGRNKMIAKLGSACNKPNLQTVV
CsRad30(3)    ----------------T--RD------PE-----------YTL-DWDDVVLDIGASIVRNIRKEIKAELELTTSAGISHNKMIAKVASRMNKPFGQTII
NgRad30       ----------------G--ES------EE-----------DDP-EWDDVVIQVGAEIVKFVRTAVWDQLKYTCSGGIARNKMMAKLGSACNKPNRQTIV
McRad30       ----------------G--KS------EE-----------EDP-EWDDIVMLVGSEIVKSIRAAVWDQLKYTCSGGIARNKMIAKLGSACNKPNRQTIV
MmRad30(3)    ----------------E--EL------ES-----------DDP-DWDDICLSIGAEIIRHVRKGVWDALHYTCSGGVARNKMLAKLGSGFNKPNKQTVI
MrRad30       ------TP----------------------------------L-TWIDVAHAMAAERMISVRWHILNELGYTTSAGIASNKTLAKLCSSFRKPCSQTML
DhRad30(1)    ----------------T--ET------EM-----------DDP-DWNDVALLIGSEIVRDVREAIRAQLKYTCSAGVASNKMLSKLGSGYKKPNGQTVI
BgRad30       ----------------K--ET------EE-----------DYP-DWDDIAVVIGSEIVRNIRANILEKLRYTCSAGIAQNKMLAKLGSAHKKPNQQTII
AfRad30       ----------------S--ET------ED-----------DDP-DWDDIAMLLGSEIVRSVRAAIRERLKYTCSAGIARNKMIAKLGSAHKKPNQQTIV
SpRad30       --------------VDR--TK------ED-----------YER-DWDDVFLFYAAKIVKEIRDDIYLQLKYTCSAGVSFNPMLSKLVSSRNKPNKQTIL

CaRad30(2)    RNSAINSFLTNFELTDVTGMGGKLGESIINKVNVP-PQIN-SISFIR-----E--------NF---------SDASIKEKLGGELGL--KVYNIVRGIN
CaRad30(3)    LSRWINQYMGILPILKLRLLGGKLGKLVSEKLPMVRMSS-------------DL------LQY---------NKSTLIKLFGEK--NGEYLYNTCRGID
NcRad30       RNRSIQHFLSDFKFTKMRNLGGKLGEQISQMFHTD-TVK-------------DL------LSA---------SVEQLKSKLGDD--TGVWVYNTVRGID
DhRad30(1)    RTCSMNNFLKNFQLIDFNGMGGKTGDVIMQRLEVP-PDVN-SISFIR-----N--------NF---------SLEDIQKEFANDPPLAQKTYQMVRGNY
UmRad30       RPCSVANFFSSLPFQKIRFLGGKLGNAMGAEWNSA-TVS-------------DL------WGV---------GLDEMQAKFGE---EARWVYNVLRGID
YpRad30       RAGALNNYLKKKSLTDIGGMGGKLGEEVLEKLGLD-KESKDNIT--------QV------QAL---------SKQQLQQKLQNA-SLTEKVFNLARGNL
CmRad30       LSRWINQYMGILPILKLRLLGGKLGKLVSEKLPMVRMSS-------------DL------LQY---------NKGTLIKLFGEK--NGEYLYNTCRGID
StRad30       RNRSTYSFLNNFELNDVTSMGGKTGDFVLQKLRVP-PD-K-GISYIR-----E--------NF---------TLEAIEEEFNDDIPLAKKIYEIVRGNH
AnRad30       RNRAIQNFLSGFKFTKIRMLGGKLGDQVTAMFGTE-QIS-------------EL------LKV---------SLEQFRAKLNDD--TAVWLYGIIRGED
GcRad30       RRHAVSKFMAEFELTDVGGLGGKLGESIMKKLELP-EHG-------------SI------RHLQEQIAAAGGGKSALLKRLDDPA-LAKRVVAIVEGNE
OoRad30       RNRAVTHFLSSMKFTSIRNLGGKLGSEVATAFGTE-NVS-------------EV------LSV---------PLETFKAKLGDD--TGTWLYKTIRGID
FoRad30       RNRAVSAFMAGFKITKLRNLGGKLGEQIVSTFNTE-SVI-------------EL------LDV---------PLATMKTKLGHD--TGFWIYNTIRGID
PgRad30       RNRAVQNFLGGYKFTQIRMLGGKLGDQITAAFGTE-KVS-------------DL------LNV---------PLEQLRTKLADH--TATWLYGIIRGDD
CsRad30(3)    RRKSIPAIMPTLKVTSLSGLARQLGQRVVKTFGSD-GIR-------------DL------LQV---------SLAEMRSKLGAQ--DGQWVYRAIRGDE
NgRad30       RNRAIQQFLSGYKFTKIRSLGGKLGKKIASEFETD-KIS-------------DL------LNI---------PLDRLKNKLDDD--TGVWLYQIIRGED
McRad30       RNRAIQQFLSGYKFTKIRSLGGKLGKRVASEFETE-KIR-------------DL------LNI---------PLDRLKNKLDDD--TGTWLYQIIRGED
MmRad30(3)    RTRAIQKFLNDFKFTKIRNLGGKLGDEIVAAFGTD-LVK-------------EL------CDV---------PVEQL-QKLGDD--TGVWLYNTLRGID
MrRad30       LPRYTCAFLAPMPYRKIRFLGGKFGADIEGEWSQS-TVR-------------EL------WGV---------SLLDMEKRFGA---DGKWLYHLIRGID
DhRad30(1)    RHRAIRQFLSGFKFTKIRNLGGKLGEQIAQTFNTE-SIG-------------DL------LLV---------SVEQFKSKLGDD--TGVWVHNTIRGID
BgRad30       RRRAVESFLSKFNFTQIRGLGGKLGEKIIQEFATE-KLD-------------HL------LSI---------SHHKLKQALGDE--TGTWVYQIIRGQD
```

```
AfRad30      RNR-VQQFMDAFKFTRIRNLGGKLGDEVVNKFGTD-QVK-------------EL------LAA---------PVEQLKAKLGDD--TGTWVYHIIRGED
SpRad30      TKNAIQDYLVSLKITDIRMLGGKFGEEIINLLGTD-SIK-------------DV------WNM---------SMDFLIDKLGQT--NGPLVWNLCHGID

CaRad30(2)   AIEL---QSTIEVKSMTSTKNF-TSF--VISNLFDAYDWLKVFAGDLHNRLIDLDNENMELSSTELSNKSK--GIM----KRPKTLTLGVL-S-KNG--
CaRad30(3)   LEAVI---ETQHCRSILSSKNFY--S--GLDNLDEIFKWLHIFSSELSERSKNLY---------------K--TLK----IRPTKVGVTIR-N-KL-S-
NcRad30      TSEV---NPRVQIKSMLSAKSFR--P--SITSFEQAVRWLRIFAADIFSRLVEEG--------------VL--ENK----RRPKTINLHHR-H-G--T-
DhRad30(1)   RQEL---SLRTEIKSMMSRKNFLSKN--PVNNIHDAYDWIKVFAGDLYGRLIELDDESLNLSMSQESRRGK--GYI----RRPKTISIQIS-S-TSY--
UmRad30      YSEV---RERVNNQTMLASKSVR--P--AITKPEEATHWLDILSTELAIRLREAR--------------EE--RRN----LWPKTLVLRYI-R-AG-S-
YpRad30      YRGL---KTRIELKSMLSAKNF-ARV--PLKDRKEAELWLVVFAGELAMRIVEHEKE-------------L--GTC----RRPRTVSLQHA-R-FKPM-
CmRad30      LEAVI---ETQHYRSILSSKNFY--S--GLDNLDEIFKWLHIFSSELSERSKNLY---------------K--TLK----IRPTKVGVTIR-N-KL-S-
StRad30      RREL---VNRMDVKSMMSRKNFLAKH--PVETLSDADSWIKVFAGDLYNRMIELDEENLRLSMSQVSNKEK--GVI----KRPKTLSINLG-T-SSG--
AnRad30      KSEV---NPRTQIKSMLSAKSFR--P--SINTLDQADKWLHIFAADIYGRLVEEG--------------VL--EHK----RRPKTIALHHR-Q-GA---
GcRad30      AAPVGNGDATTTTKSMMATKNFA-EA--PVSTAMDARAWLRVFAAELESRLLELS--------------ESTTHIT----LYPKTITMTHK-L-AG-AG
OoRad30      TTEV---LTRTDIKSMLSAKSFS--P--AITRYQQGENWLKVFCADIVSRVNEEADEPVEYSPSHDDNAAL--GSQPVSLRRPRTMTLHHR-S-G--K-
FoRad30      TSEV---NSRTQIKSMLSAKSFR--P--TINSSEQATRWLRIFAADIFARLVEEG--------------SL--ENK----RRPRTMNLHHR-H-E--G-
PgRad30      RSEV---NPRTQIKSMLSAKSFR--P--SIKSLDQAEKWLRIFAADIYGRLVEDG--------------VL--ENR----RRPKVITMHHR-T-GN-A-
CsRad30(3)   TGPV---RPRSEVQSLLAAKTFV--P--KADNLQQASKWLRIFAADLESRLRDLDL-------------DS--EVP----RRPKTIAVQHHIRGRF-G-
NgRad30      DCEV---TPRTEIKSMISAKSFN--P--KLVSLDQGEKWMRIFVAEIYGRLIDEG--------------VL--ENK----RRPKMITVHHY-G-P--N-
McRad30      DSEV---TPRTEIKSMISAKSFN--P--KLNALDQAERWMRIFVTEIYGRLIDEG--------------VL--ENK----RRPKMITVHHH-G-S--N-
MmRad30(3)   SSEV---NSRTQVKSMLSAKSFR--P--SINTFEQGVRWLRIFAADIFSRCVEEG--------------VL--ENK----RKPKTITLHHR-Q-G--G-
MrRad30      TSNV---VQRSANHSMMSAKNFR--P--GISSTAVALSWIAIMSSELSMRLQEER--------------EE--VKM----MYPRTLVLRYL-L-AD-S-
DhRad30(2)   NSEV---NPRVQIKSMLSAKSFR--P--SINSVESAIKWLRIFVSDIFSRLVEEG--------------VV--ENK----RRPRSINLHHR-H-G--G-
BgRad30      YSEV---SSRTQIKSMLSTKSFR--P--SITTYEQAYSWLRIFAADLYSRLVEEG--------------VL--ENR----RRPKTIHLHHK-N-G--A-
AfRad30      SSEV---NARTQIKSMLSAKSFR--P--TINTSEQAVRWLRIFVADIQSRLVEER--------------VL--ENK----RRPKVLSLHHR-Q-G--A-
SpRad30      NTEI---TTQVQIKSMLSAKNFSQ-Q--KVKSEEDAINWFQVFASDLRSRFLE-----------------L--EGM----RRPKTICLTVV-S-R--F-

CaRad30(2)   --T--RQTRQMQIPIHKD--L-D-KMKDIFFENGCVL-LREFLE-F--NTHISLLNNRTPAKEIFI---WDPKKVKIMELINMSLTISKFVSVEDRFT-
CaRad30(3)   --Q--NRTRVQSINYSDH--T-P--NTDIIYETAKFIFLNRFYE-P--NNSNIQKKESDI-NTSY--------IKDIFPCKYLGVSLHNLIKDDSKFD-
NcRad30      --Q--TRSRQGPIPQGRK--L----DEESLLELARNL-LSQIVS-E--GQ---------------------------VPCSNLSLSVGGFEDGVTG-N-
DhRad30(1)   --V--KHSKQMQLTIVRS--L-E-KLKENMEVTGLRL-LMDILD-N--STNVHKLNNGISLKELDKKIDKDYSKINIIPLANMSLVISNFVKNTDSSL-
UmRad30      --V--PRSRQTAFPFTNP--NNQDDLAKVILKKGQKL-WEESLG-D--ALGLGLGS-----SG----------F-GEAKVLTIALGFGSLAEGEAG-Q-
YpRad30      --V--KRSRQQDLPLVHT----N-KLKEELFAAGVKL-LKLIEN-E--PEH------------------------DAYPISMLSLSVG-FVELPAGAS-
CmRad30      --Q--NRTRVQSINYSDH--T-P--NTDIIYKTAKFIFLNRFYE-P--NNSNIQKKESDI-NISY--------IKDIFPCKYLGVSLHNLIKNDSKFD-
StRad30      --I--RRSKQAQLMQYSS--L-D-RLWEHFEQIGYRI-LRDLLE-T--ITETAKLNGGRTLKELEAQS-LDPRKIKVASISHLSLTISNFIKANDSSL-
AnRad30      --Q--VKSRQLPIPGSAT--I----DEDLLFELGKTL-LRQIAA-D--GA--------------------------AWPCANISMSVGGFEDGTSK-N-
GcRad30      AGPTGSQNKQVSFPSQVTPAR----LRDTLYELGCAL-LAMFEHPEGSGN--------------------------AYPCALLALGVSNFVDNSAL-LT
OoRad30      --I--TKSRQAGIPVGKV--F----DEELLLPIARNL-LKQIEQ-E--GR--------------------------CWPCGNLSLSVGGFDDIGRGSN-
FoRad30      --Q--VRSRSGPIPQGRI--I----DEGSLFELAKDL-LLQIIA-E--GR-------------------------GVWPCANLSLSVAGFEDGVKG-N-
PgRad30      --Q--SHSRQIPIPGSNA--I----SENLLYDLANTL-LRQVVA-D--GQ--------------------------AWPCSNLSLSVSSFEDGVIN-N-
CsRad30(3)   --P--TRSKQAPIPPHVE--I----NRETIFNMLHGL-LKDLTD-H--GE--------------------------SWPCLGISVSMSNLESGLPTSD-
NgRad30      --Q--DKSRQTHIPTGGV--I----DQTMLFDLAKNL-LQQVVS---------------------------------WPCSHLSLTVSGFESGITG-N-
McRad30      --Q--DKSRQTHIPPGGV--I----DQTMLFDLAKNL-LQQVIS---------------------------------WPCSHLSLTVSGFETGITG-N-
MmRad30(3)   --T--TKSKQALIPQGGL--I----SEELIFDLAKSL-LAQIVV-D--GR--------------------------AWPCSNLSLSAGGFEDTITG-N-
MrRad30      -----TSMKSHQVPFGKI--ANE-HLDHEIYVRAEKL-WNETLG-R--AM-----------QQ----------P-GRIDVRVLSLSFAGIERKMKD-Q-
DhRad30(2)   --Q--TRSRQGPIPQGKP--I----NKELLLELAKNL-LNQVVL-E--GK--------------------------VWPCANLSLSVGGFDEGITG-N-
BgRad30      --Q--TKSRSGLIPLGRK--I----KENDIFELARSL-LDQMNQ-D--GQ--------------------------AWPCANLSLSVGGFENGVTG-N-
AfRad30      --Q--MRSRQASIPSGRP--I----DEDLLFDIAKHL-LGQIII-D--GR--------------------------AWPCSNLQLSVGGFEDGPTG-N-
SpRad30      --L--RKSRSSQIPMNVD--I----STQFIVEATSKL-LRQLQQ-E--FD--------------------------VYPISNLSISFQNIIEVDRN-S-

CaRad30(2)   -LLKTTD--------------------------------------------------------------------------------------------
CaRad30(3)   -------------------ILQFDISNNK-TN-N-K--LRYDE---------------NI------IEN--------YK-NE-NKNNIQ-EFDNN-S-L
NcRad30      -----------------MGIGAFLLKGEE-AQ-ASKT-GSGTA--------------T-V------DS---E-----TE-IR-PAEKRR-RLDNG-G-I
DhRad30(1)   -IDSYTDNKSD--ISTQENIRRMFDEVNQ-ETRIKRE-KLEDV--------------T-P------KP---E-----AS-KS-KSRSIS-KEEVE-Y-V
UmRad30      -----------------QGIAGFLGTASG-STATAST-FPLPA--------------A-LAEGPEITR---LAAEVDSE-EH-SSPKKKRKRNR----L
YpRad30      -------------------IGHFFKTITT-EAREKAA-VEKTK--------------A-Q------QV---N-----TLWNK--PKEEK-KSSGK-D-I
CmRad30      -------------------ILQFDISNNK-TN-N-K--LRYDE---------------NI------IEN--------YK-NE-NKNNIQ-EFDNN-NSL
StRad30      -IDSYTGNTETDKESAQAKLKQMFDEVNK-SALEKQE-KEPSP-----------------------------------A-PQ--PRIIS-SEDKS-Y-I
AnRad30      -----------------QAIDGFLLRGDQARN-MASS-SSSRV--------------H-V------------------A-DE-VPAEHQ-PIE-KRR-K
GcRad30      KN---------------RSLTSFFKAVPKKKT-PQIE-Q--SG--------------L-S------SG---N-----NSSSK-STD-KA-NLPVTSR-G
OoRad30      -----------------RGIGGWLVKGEE-AK-ALKE-ESLKR--------------R-SL-----AI---E-----EG-RV-ETTKKR-KVGIDTF-F
FoRad30      -----------------MGIGAFLVKGEE-AE-ALRS-S-IPD--------------S-R------QS---S-----TG-PE-PSAKKR-RVEDG-G-I
PgRad30      -----------------KAIEGFLVRGDQAK--ALSH--SSRP--------------R-D------------------A-DN-SPSEQA-STE-KRR-K
CsRad30(3)   PG---------------QRITSFFSSNSSASP-RKR-------------------------------------------------------IREG-D-V
NgRad30      -----------------KSLDNFFIRGAGETP-VSRR-GSNIP--------------N-E------------------S-CQ-SEPPDD-NVG-HQR-K
McRad30      -----------------KSLDSFFIRGGSDSS-VSRR-DGSTP--------------K-I------------------D-CR-AELPDD-NTAYHQR-K
MmRad30(3)   -----------------MGIKGFLVTGGQRPT-KEVV-DSSEA--------------S-D-----------S-----EE-EP-PTAKRQ-KIDVDDG-I
MrRad30      -----------------QPLSNFFSKRKS-DHDAKVA-L------------------------------------------------------------
DhRad30(2)   -----------------MGIGAFLVKGEE-AQ-SLKR-SSREG--------------S-V------EE---P-----PS-KQ-P-EKHR-KFEKE-G-I
BgRad30      -----------------MGIGNFLIKSND-IK-AMHM-DGPNA--------------P-------------D-----DS-IE-RLDKRR-RLDNTTG-I
AfRad30      -----------------RGIGSFLVRAER-AK-AMIP-SIRDA--------------D-GY-----EW---S-----KV-ND-QPDKRR-KLDHG-A-I
SpRad30      -----------------RGIEGFLKKSND-EI-YMSTS-VSPSIEGRAKLLNENMRENNSF-----ELSSEK-----DI-K--SPKRLK-RGKGK-G-I

CaRad30(2)   ---------------------------------------------------------------------------------------TSFNK-------
CaRad30(3)   DILQST---------------------------------------------------------------------------------------------
NcRad30      ERFFAKR---------------------------EL----SH--------------GA------N--P--GL--GSD-DT—AISGQSGQRLGT------
DhRad30(1)   NKLFTDF---------------------------NSEQTLASNTRNPSK--------S--------TR--GPR--DL-K-T-N----NSQED-------
UmRad30      DVMFETQ---------------------------SK----QL----------------------QSQA--SH--SKQ-K-E-GPTKDNRDDDTE-----
YpRad30      TQFFAK----------------------------------------KP---------K--------EK--KDS--DE-T-T-K----KP----------
CmRad30      DILQST------------------------------------------------------------------------------KIINQLET-------
StRad30      KKLFDDF---------------------------QMSNHIEEASKSPKR--------S--------EN--KAQGSSM-K-T-S----SEKHD-------
AnRad30      VEDSG------------------------------L--------------------RR------F--F--NK--PSE-T-DGPGHSFDK-GHMA-----
```

```
GcRad30      SP-AGG-----------------------------M----------------------------------------Q-S-T-ANSTGESDST-------
OoRad30      SRGRAGEEVYDRGIGFDDKEDIEVDDGFFHSDENEK----EE------MGFDLEVEDD------L--Y--GG--PSK-D-S-PLENHHSSDPTTTNYKN
FoRad30      QRFFSKR---------------------------PS----TD--------------HERTSLTDS--H--TH--GEDSKAG-SLPSDPTQKTLSFATK-
PgRad30      LDGDGGK----------------------------I--------------------KR------F--F--GN--PSH-L-EDT----------------
CsRad30(3)   PNRHAD-----------------------------S--------------------KR------V--QVSSD--ATE-D-P--------APV-------
NgRad30      KQRVG---------------------------------------------------ET------H--E--QN--PSK-A-V-GFFSRYRTNTNT-----
McRad30      KQRVG---------------------------------------------------EK------H--Q--SN--SST-A-G-GFFSRYKVRSRE-----
MmRad30(3)   RRFFAH--------------------------------------------------------------PSE-A-A-TSTHNENQAT-------
MrRad30      -----------------------------------------------------------------------K--LPRTQ-S-PPY--------------
DhRad30(2)   QRFFSRS---------------------------AT----TR--------------Y-----------------GDE-DR--AMNPATGWVTRG-----
BgRad30      HRYFHKN---------------------------AY----FQ--------------NL------D--N--GL--GAQ-R-L-SVDKLEHRGS-------
AfRad30      QDYFSGQ-------------------------------------------------ES------Q--E--EQ--HSD-A-A-NVDNADPEAV-------
SpRad30      FDMLQQ-----------------------------------------------------------------------------------T---------

CaRad30(2)   ------------------EEHIAKLFRDYEQAPGTIKYSSPPPPPPPAQKKRSKNGQLD--------I-FESLKK---KSKPG------DF--------
CaRad30(3)   ---------N---------PNI-------------------------------------------------KEDI---IELS------SD---------
NcRad30      ---------LK---EEVMSGE------P-------------------------------AGSEGVG--K--GDEV---YNGK-----S-ES--------
DhRad30(1)   ------------------KKYIRSLFDKFESDSNVMNVPSKSQPERKEEKKYNILKDLK--------A-KSSSHS---VKSSKAVANDQTF--------
UmRad30      ---------Q---------PRVD----I-------------------------------DE--EAG--VKVVEAA---SGPQ-----A-----------
YpRad30      ---------------------------EAS-----------------------DPEVKKPL-S-SEANDYNSSSL---FVFS-----D-DE--------
CmRad30      ---------N---------PNI-------------------------------------------------KEDI---IELS------SDS--------
StRad30      ------------------SKYIKKLFEDYQTQA-SLDLTLKSNSGIQHAKEENTKRKLD--------I-FQSLQK---RQKPQSKSTD-DF--------
AnRad30      ---------DT--------PDIN----V-------------------------------DN--AQL--DDHVA-------SD-----G-EI--------
GcRad30      ------------------SVNED----AGP-----------------------------TT--KNN--NDSDDEQKEEEEEE-----E-ES--------
OoRad30      LDRNAPPPSTIRTSSSVRTRFFS----K-------------------------------EN--TPT--TPITTDP---TKPT-----K-SQ--------
FoRad30      Y--------DC---EARHESDLA----M-------------------------------HESH--A--SPWHESA---FDVQ-----V-DQ--------
PgRad30      -------------------ATDE----P-------------------------------NS--AQE--P--VE-------MD-----A-----------
CsRad30(3)   ------------------RLAT------------------------------------------AS------TSA---WAVP-----G-DP--------
NgRad30      ---------K-----------ET----P-------------------------------AE--PCS--EKAGESR---PDLQ-----T-NP--------
McRad30      ---------QP--------PIKQ----P-------------------------------PE--TCP--EEVAESR---AGLQ-----T-NP--------
MmRad30(3)   ------------------QARFP---------------------------------------------------T------E-----T-----------
MrRad30      ---------------------------------------------------------------------DL---V---TDPT-----------------
DhRad30(2)   ---------ET---SDKHVGNSM----P-------------------------------EWDP--A--D--AVAR---NEAS-----K-DS--------
BgRad30      ------------------SLSLA----A-------------------------------LS--HFQ--TR-EDGI---EAMT-----I-EP--------
AfRad30      ------------------SLSPP----D-------------------------------TS--SAK--RDGDEDD---EVIT-----L-DETAGKSAGS
SpRad30      -------------------------------------------------------------------------------AVS------KPTE-------

CaRad30(2)   ----LEELIKTKKCSRC--K---------------------LSVD-DP-VEHNDYHIAMDLSNKLNN--------------------------------
CaRad30(3)   -------SDKTYNCNICHQIL---------------------K--LSD-KQHDKYHLNSKLQSSKYQDPMKK--QGI----------------------
NcRad30      ----EPGPIITLTCSRCNAS---------------------LDSPEEL-QSHQDWHFAKELQEQERVS---Q--T-F----------------------
DhRad30(1)   ----FDDLVSNNYCSHC--N---------------------LEVK-DV-FEHRDFHVALELSSKINGR-------------------------------
UmRad30      ----DQEDDPHWRCPDCSHLIQAPS-------GSAWFEKPYLVE-KLK-DEHLDWHFAVTLSQAPQVADSAG--A-S----------------------
YpRad30      ----MDEYIDTFTCPKCDKK---------------------YPID-EE-MEHSDWHVAVELSKANRPEPKP----------------------------
CmRad30      ----DKSEDETYNCNICHQIL---------------------K--LSGKQQHDKYHLNFKLQSSKYQDPINK--QR-----------------------
StRad30      ----LNTLLQTMYCPQC--K---------------------EEID-NA-VEHNDFHVAMEISNRING--------------------------------
AnRad30      ----EQVHSKSFRCPRCGRY---------------------INCGE-E-DEHNDWHFAKDLETQERQAARSS--Q-A----------------------
GcRad30      ----DLFVTQLYTCPECQAK---------------------LDP-HKR-SEHADFHFAQRLAAGTTTSWSSPGSR-S----------------------
OoRad30      ----YMEEETAFPCSRCNNK---------------------LIPLDKL-DEHSDWHFAKDLLQEDRIVRPAS--S-N----------------------
FoRad30      ----KQHSLTDVVCSRCKAS---------------------FADPEAL-QNHRDWHMAKDLQDAERVK---P--T-F----------------------
PgRad30      ----ELSGIPRFVCPRCSKS---------------------MFEYE-K-EEHDDWHFAKDLASQDREEAKVS--Q-L----------------------
CsRad30(3)   ----RGEQEQQYLCPKCDES---------------------VLPED-V-LEHLDWHVALEIQNQES---------------------------------
NgRad30      ----ASYYSSQGVCSRCGET---------------------MPDFM-Q-LEHDDWHLAKALASQEQ---QP-----L----------------------
McRad30      ----T--FSPPGICSRCGET---------------------VPDFM-Q-VEHDDWHLAKDLESQEQ---RP-----L----------------------
MmRad30(3)   -----DVLDGIYICKKCSKP----------------------ILQSDA-GEHEDWHFAKNLENELRQE--PR--P-A----------------------
MrRad30      ----SQTEMAQWTCLKCSHVLSVPIFEDVEPHATEPPSYLGILQ-RAC-EEHEHWHMALALAERLE---------------------------------
DhRad30(2)   ----QLQEPSTYLCRRCDTV---------------------CEDAEEL-QCHQDEHLARDFYEEERGS---H--T-F----------------------
BgRad30      ----RHQGESDFLCTRCHMN---------------------LECADTL-QNHLDWHLARDLQDKESQ----K--I-S----------------------
AfRad30      LCQQAPITTYEYFCDACRKR---------------------FPTV-AK-EEHEDWHLARELQAKEKSICRSE--P-G----------------------
SpRad30      -----NSADETYTCEECEQK---------------------ITLS-ER-NEHEDYHIALSISRKERYNNLVP--PSHDKPKQVKPKTYGRKTGSKHYAP

CaRad30(2)   ---------------------------------------------------------------------------------------------------
CaRad30(3)   --------------------------------------------------------------SLL-N--SSLKI-NEKKPHNLR--RKRS---LNIYTS
NcRad30      -------------------------------------------------------------VNQPSA--SSSRAGNQKSTSTMP-----K---RQGRPK
DhRad30(1)   ------------------------------------------------------------------------EI-TPETTNKRSGSPYSEREPKSS---
UmRad30      -------------------------------------------------------------ASQR-P--ATT TA-KATAAGTSK--KKRA---DKFFA
YpRad30      ------------------------------------------------------------------------LK----VVKPKG-----------K---
CmRad30      --------------------------------------------------------------TLL-K--SPLKI-NEKKSHNLR--RKRS---NIYTS-
StRad30      ---------------------------------------------------------------------------------------------------
AnRad30      -------------------------------------------------------------AQLP-N--RAASG-SR--SKPAR--G-G----RGGKSE
GcRad30      -------------------------------------------------------------TNTA-AAVGSPRVAKRKPPSTAT---KKK-KKPA----
OoRad30      -------------------------------------------------------------NASS-S--SSTTT-ASTSNTSKN--GKKD---EKQETS
FoRad30      -------------------------------------------------------------AERQPA--ARN--SAQKTQ-GTT-----S---RRSRGG
PgRad30      -------------------------------------------------------------PPPT-K--NSTRG-MNTRGRGSR--G-GG---SRGKPE
CsRad30(3)   ---------------------------------------------------------------------------------------------------
NgRad30      -------------------------------------------------------------SAAR-V--R-------------------------GAGK
McRad30      -------------------------------------------------------------NAVR-P--R-------------------------GAVK
MmRad30(3)   -------------------------------------------------------------PVHQ-V--ESHAK-EKVKEKTKT--KRTA---QGHQST
MrRad30      ---------------------------------------------------------------------------------------------------
DhRad30(2)   -------------------------------------------------------------AGNSAL--ATT--GNSKGASATT-----K---RPAKRK
```

```
BgRad30        -------------------------------------------------------------LINH-P--VKNSW-ERVS--STH--MPNS---GKGKSK
AfRad30        -------------------------------------------------------------SDRD-P--HGSAW-SSGSVKRST--VPRA---GNSNSN
SpRad30        LSDETNNKRAFLDAFLGNGGNLTPNWKKQTPKAISNSSDNMTQLHLDLANSTVTCSECSMEYNST-S--EEDIL-LHSRFHSRV--LGGV---TVSFQC

CaRad30(2)     ---------------------------------------------------------------------------------------------------
CaRad30(3)     -------------------------------------------------------------------------------EDTLKQT-------------
NcRad30        -------------------------------------------------------------------------------KVERGQS-------------
DhRad30(1)     -------------------------------------------------------------------------------KVDKGQS-------------
UmRad30        ---------------------------------------------------------------------------------------------------
YpRad30        -------------------------------------------------------------------------------RKGDRQA-------------
CmRad30        -------------------------------------------------------------------------------EDTLKQT-------------
StRad30        ---------------------------------------------------------------------------------------------------
AnRad30        ----------------------------------------------------------------------------------KGQT-------------
GcRad30        -------------------------------------------------------------------------------VFDKTQT-------------
OoRad30        SQ---TVVPVK------------------------------------------------RGRGRPPKHAIVHVQGDNGPVLEKGQK-------------
FoRad30        -------------------------------------------------------------------------------KLEQGQS-------------
PgRad30        ----------------------------------------------------------------------------------KGQM-------------
CsRad30(3)     ---------------------------------------------------------------------------------------------------
NgRad30        ----------------------------------------------------------------------------------TRQT-------------
McRad30        ----------------------------------------------------------------------------------TRQT-------------
MmRad30        -------------------------------------------------------------------------------KVEKGQQ-------------
MrRad30(3)     ---------------------------------------------------------------------------------------------------
DhRad30(2)     -------------------------------------------------------------------------------KMEAGQS-------------
BgRad30        ----------------------------------------------------------------------------------KVQS-------------
AfRad30        SN-----------------------------------------------------------------------------SNSTTTT-------------
SpRad30        SPIYRVNYGLSSDCIYSINSESSLIDQRKAEEALSFVNNELSSEPIETIGVDKYTTFLFISDKKCVGL-----------LLAERISSAYIVDELELNNN

CaRad30(2)     -------------------------------------------------H--------------------------------------------
CaRad30(3)     ----------------------------------------------KLNI--------------------------------------------
NcRad30        ----------------------------------------------KLKFG-------------------------------------------
DhRad30(1)     ----------------------------------------------KLPF--------------------------------------------
UmRad30        -------------------------------------------------RR-------------------------------------------
YpRad30        ----------------------------------------------RLAF--------------------------------------------
CmRad30        ----------------------------------------------KLN---------------------------------------------
StRad30        -------------------------------------------------H--------------------------------------------
AnRad30        ----------------------------------------------RLTFG-------------------------------------------
GcRad30        ----------------------------------------------RLNF--------------------------------------------
OoRad30        ----------------------------------------------KLAFGRG-----------------------------------------
FoRad30        ----------------------------------------------RLKFG-------------------------------------------
PgRad30        ----------------------------------------------RLAF--------------------------------------------
CsRad30(3)     ----------------------------------------------------------------------------------------------
NgRad30        ----------------------------------------------KLAFG-------------------------------------------
McRad30        ----------------------------------------------RLAFG-------------------------------------------
MmRad30        ----------------------------------------------RLRFGNG-----------------------------------------
MrRad30(3)     ----------------------------------------------------------------------------------------------
DhRad30(2)     ----------------------------------------------KLNFG-------------------------------------------
BgRad30        ----------------------------------------------RLQFGGQDTNG------------------------------SV-----
AfRad30        ----------------------------------------------QKKMEKMEKGQSR---------------------------LAFGN---
SpRad30        NSTSSAVYIKNENLRKGFVLGISRIWVSASRRKQGIASLLLDNALKKFIYGYVISPAEVAFSQPSESGKQFIISWHRSRNNGSSKSLRYAVYES
```

PCNA – Buffer titration

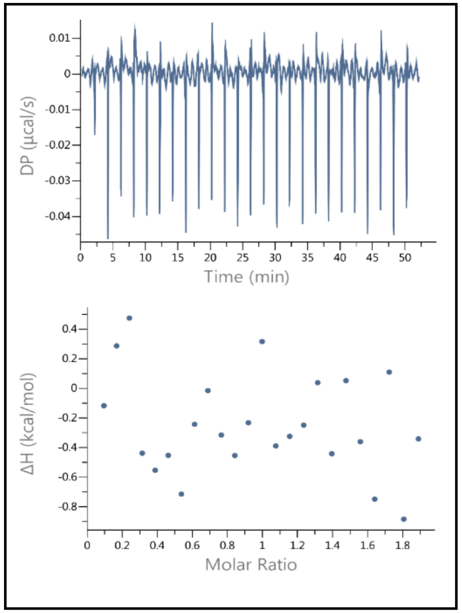

Fig. S2

A.

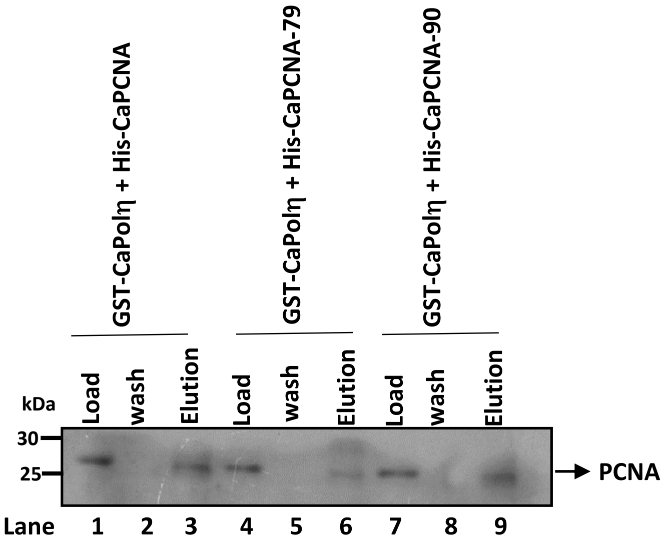
GST-CaPolη + His-CaPCNA
GST-CaPolη + His-CaPCNA-79
GST-CaPolη + His-CaPCNA-90
kDa
Load
wash
Elution
Load
wash
Elution
Load
wash
Elution
30
25
PCNA
Lane 1 2 3 4 5 6 7 8 9

B.

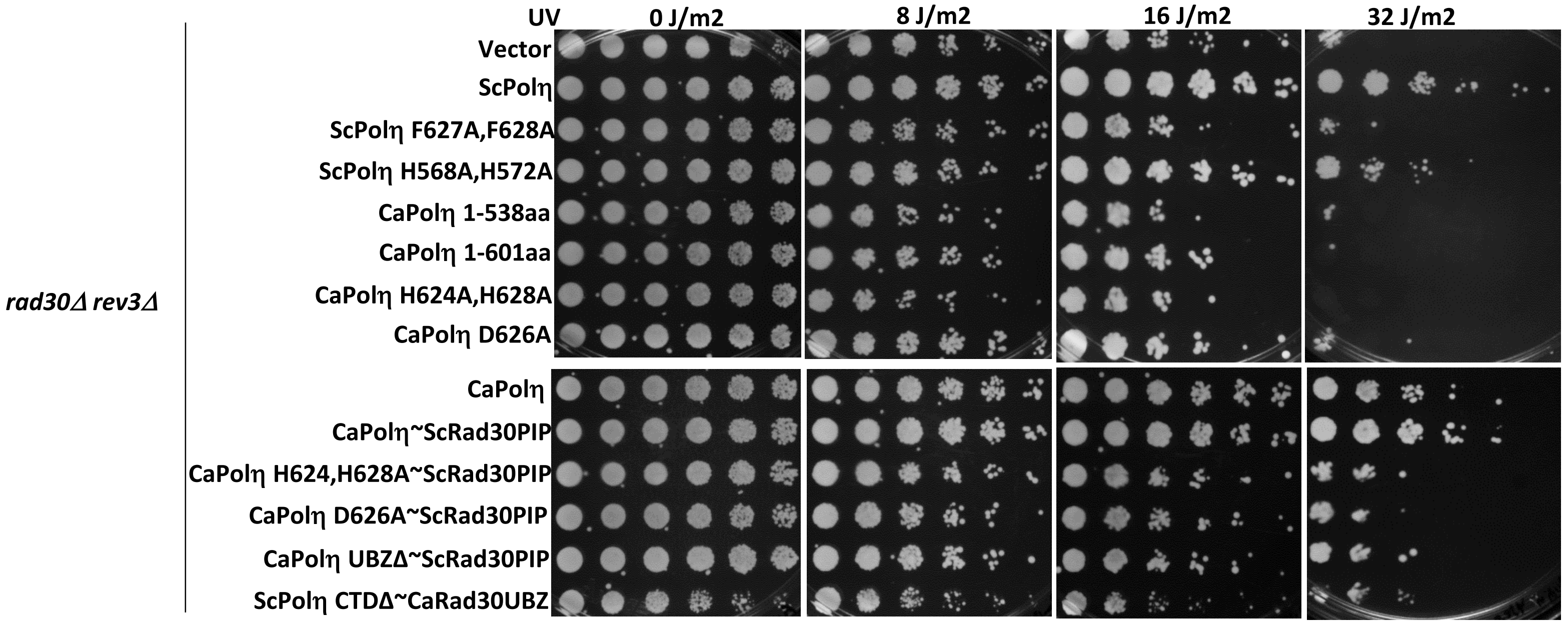
UV
0 J/m2
8 J/m2
16 J/m2
32 J/m2
Vector
ScPolη
ScPolη F627A,F628A
ScPolη H568A,H572A
CaPolη 1-538aa
CaPolη 1-601aa
CaPolη H624A,H628A
CaPolη D626A
rad30Δ rev3Δ
CaPolη
CaPolη~ScRad30PIP
CaPolη H624,H628A~ScRad30PIP
CaPolη D626A~ScRad30PIP
CaPolη UBZΔ~ScRad30PIP
ScPolη CTDΔ~CaRad30UBZ

Figure S3
